# Elucidating the Impact of Red Blood Cell Membrane Components on Melittin-Induced Pore Formation with Molecular Dynamics Simulations

**DOI:** 10.1021/acs.jpcb.5c04289

**Published:** 2025-09-22

**Authors:** Joshua D. Richardson, Reid C. Van Lehn

**Affiliations:** † Department of Chemical and Biological Engineering, University of Wisconsin–Madison, Madison, Wisconsin 53706, United States; ‡ Department of Chemistry, University of Wisconsin–Madison, Madison, Wisconsin 53706, United States

## Abstract

Understanding the membrane-disrupting mode of action of antimicrobial peptides (AMPs) in complex biological membranes is critical for the design of therapeutically viable AMPs that are both active against microbial pathogens and nontoxic to human cells. To assess human toxicity in AMP design studies, melittin (MEL) (a highly charged 26-amino acid AMP sourced from bee venom) is often used as the positive control in experimental human red blood cell (RBC) hemolysis assays. Molecular dynamics (MD) simulations have proven invaluable in elucidating the pore-formation mechanism of MEL in single-lipid zwitterionic membranes. However, modeling pore formation in lipid bilayers containing multiple lipid species, like RBC membranes, has been limited due to the challenges of using atomistic MD simulations to capture long-time-scale membrane restructuring events that depend on lipid heterogeneity, leaflet asymmetry, and cholesterol content. To address these challenges and access larger time scales, in this work, we utilize the coarse-grained MARTINI force field to model four lipid membranes of increasing complexity, ranging from single-lipid POPC membranes to asymmetric RBC-mimetic membranes containing cholesterol. Through the application of a nucleation collective variable (ξ) to create transmembrane pores and a coarse-grained-to-atomistic backmapping strategy, we studied MEL pore-lining affinity and pore nucleation free energies to assess the effect of lipid complexity and cholesterol on MEL pore formation. We find that although cholesterol strongly inhibits MEL-induced pore formation regardless of lipid content, pore nucleation is more favorable in RBC versus single-lipid POPC membranes when cholesterol is absent due to the enrichment of anionic POPS lipids near the pore that permits increased conformational flexibility for MEL. These results provide new physical insight into factors that affect pore formation in compositionally complex membranes and are a step toward understanding how AMPs can be designed to selectively induce pores in membranes with different compositions.

## Graphical abstract

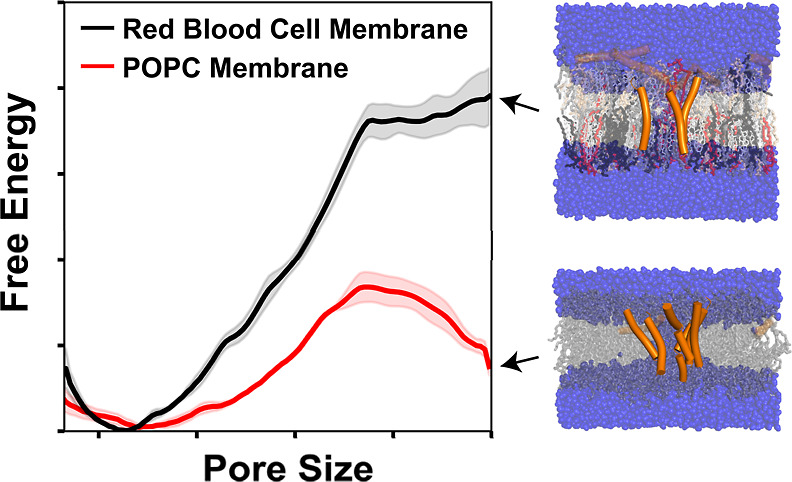

## Introduction

The antimicrobial resistance (AMR) crisis is a major global health threat.^1,2^ AMR arises from genetic changes in microbial populations over time, such as through mutations or natural selection of members that carry resistance genes,^3^ that compromise the efficacy of clinically available drugs. By 2050, it is projected that 10 million deaths globally will be attributed to AMR, a 70% increase from the current AMR mortality rates.^2^ Antimicrobial drugs that target intracellular targets (such as enzymes) are particularly susceptible to the rise of AMR since mutations in genes that encode these targets can lead to structural changes that prevent drug binding.^4–7^ As a result, an emerging drug class that is being studied for the treatment of multidrug-resistant infections is antimicrobial peptides (AMPs), whose mode of action instead involves direct binding (through strong polar and electrostatic interactions with lipid head groups^8,9^ ) and disruption (through either pore formation^9,10^ or permeabilization^11^) of microbial cell membranes. Therefore, AMPs are less susceptible to the development of AMR compared with currently available microbial drugs.

Several AMPs have been discovered that display a high antimicrobial activity against different microbial strains. Some examples include (i) perforin-2, found in mammalian immune systems, which attacks bacterial pathogens via pore formation,^12^ (ii) lectin, sourced from cyanobacteria, which displays high activity against*Cryptococcus* fungi,^13^ and (iii) buforin II, found in toads, which shows broad-spectrum antimicrobial activity.^14^ An ongoing challenge that limits the implementation of AMPs in clinical use, however, is nonselective toxicity toward both human and microbial cells at antimicrobial concentrations.^15,16^ Therefore, the design of novel AMPs necessitates the comparison of both antimicrobial and human cytotoxicity, where the goal is to maximize the ratio of the AMP concentration required for human cytotoxicity to the AMP concentration to prevent microbial cell growth.^15,17,18^ Past studies have thus sought AMPs that are highly selective toward microbes and are good candidates for clinical trials through iterative mutations of naturally sourced template AMP sequences using methods such as experimental rational design^15,19,20^ and machine learning predictive workflows.^17,21,22^

A common experimental metric that is utilized to assess the cytotoxicity of AMPs is their propensity to rupture human red blood cells (RBCs), which is referred to as hemolysis.^19,23,24^ Typically, melittin (MEL)—a highly charged, 26-amino acid AMP sourced from bee venom^25^ with the amino acid sequence GIGAVLKVLTTGLPALISWIKRKRQQ—is used as the positive control in hemolysis assays because it lyses RBCs at low peptide concentrations.^19,25^ Despite this, the exact mode of action of MEL in RBC membranes remains elusive. Neutron scattering and circular dichroism experiments suggest that MEL preferentially forms pores in simple phosphatidylcholine (PC) lipid membranes.^26^ These experiments have been supported by molecular dynamics (MD) simulations, which found that MEL cooperatively inserts into membrane defects to form long-lasting transmembrane pores in single-component DPPC bilayers.^27^ Simulations of MEL in POPC^28^ and DMPC^29^ bilayers have likewise quantified free energy minima with 4–7 pore-lining MEL peptides (i.e., peptides that insert into the pore and interact favorably with the lipids along the pore boundary, as typically depicted in “barrel-stave” or “toroidal” schematics of AMP pore formation^10,26^ ). However, mechanistic studies of MEL in more realistic multilipid, asymmetric membranes such as RBC remain to be explored. RBC and eukaryotic plasma membranes in general are characterized by significant mol % of cholesterol (CHOL) in both the upper and lower leaflets—roughly 50 mol % in each—with an asymmetric distribution of mostly phosphatidylcholine (PC) and sphingomyelin (SM) lipids in the outer (hereafter referred to as “upper”) leaflet and phosphatidylethanolamine (PE) and phosphatidylserine (PS) lipids in the inner (hereafter referred to as “lower”) leaflet.^30,31^ Membrane properties during MEL pore formation such as lipid enrichment around the pore, lipid flip-flop,^32,33^ and CHOL dynamics are all important considerations that can be evaluated when comparing MEL pore formation in RBC versus single-lipid PC membranes.^26–29^

In previous work, we demonstrated a multistep simulation approach for sampling MEL pore lining and energetics in single-lipid DMPC membranes.^29^ First, coarse-grained (CG) simulations using representations of DMPC and MEL parameterized with the MARTINI force field were performed to equilibrate multiple MEL peptides adsorbed to a DMPC membrane. Next, a transmembrane pore was induced in the membrane by biasing a nucleation collective variable (ξ)^34^ to promote MEL pore lining at a range of pore sizes. Nucleation refers to the first stage of pore formation in lipid membranes, during which membrane thinning and deformation of lipid headgroups toward the hydrophobic lipid environment of the membrane induce a thin, water-filled transmembrane defect (ξ = 1.0) in an initially flat, unperturbed membrane (ξ = 0.2). These configurations were then “backmapped” from MARTINI to the all-atom (AA) CHARMM36 force field.^35^ After additional AA simulation of these pore-lined configurations, free energy profiles as a function of ξ were then obtained from umbrella sampling^36^ in conjunction with the weighted histogram analysis method^37^ to provide insight into the energetic favorability and metastability of MEL-lined pores. This CG-to-AA approach allowed us to resolve a free energy barrier for MEL-induced pore nucleation in lipid membranes without predefining MEL positions relative to the transmembrane pore.^29^ This methodology consistently samples pore-lined MEL configurations as a function of increasing transmembrane pore size to resolve pore nucleation free energies with atomistic accuracy, without requiring high peptide-to-lipid ratios (∼1:20) as in prior CG simulations^38,39^ or computationally infeasible microsecond-to-second time scales as in prior experiments.^10^

In this work, we extend our prior workflow to systematically investigate the pore lining and free energy profiles for MEL-induced pore nucleation in multicomponent lipid membranes. We study four membranes of differing CHOL compositions and complexity produced by asymmetric distributions of POPC, POPE, POPS, and POSM lipids in the upper and lower leaflets to mimic experimentally resolved RBC lipid compositions. Given the limitation of the MARTINI model, which requires the peptide secondary structure to be fixed throughout the simulation, we also investigate two conformations of MEL: (i) a fully α-helical conformation and (ii) a “kinked” conformation with a turn at T11 ^38,40^ based on previous studies that suggest MEL may line pores in a “U-shaped” manner.^38,39^ We further introduce a new long-time-scale equilibration step before CG-to-AA backmapping to allow MEL to form reasonable peptide-lined pore structures in more complex membranes, which is particularly needed for membranes with CHOL. In the absence of CHOL, we find that lateral enrichment and flip-flop of anionic POPS lipids in RBC membranes permit favorable pore lining by MEL at smaller pore sizes compared to POPC. In the absence of MEL, POSM lipids are enriched near the fully open pore due to favorable hydrogen bonding interactions between pore waters and the sphingosine backbone of POSM. Conversely, in membranes containing 50 mol % CHOL, the increased membrane thickness inhibits pore lining, and therefore, free energy differences between bare membranes and MEL-containing membranes comparing RBC and POPC are much closer in value. Taken together, these results highlight the importance of membrane CHOL in inhibiting MEL-induced pore nucleation^41^ and provide a framework for future simulation and development of novel AMPs based on results for the highly hemolytic MEL peptide in this study.

## Methods

### Overview of Membranes and Melittin Conformations

As shown in Figure 1a, we built four CG lipid membranes: a single-lipid POPC membrane (POPC-0%CHOL), POPC with 50 mol % CHOL in both leaflets (POPC-50%CHOL), a model RBC membrane with asymmetric distributions of POPC, POPE, POPS, and POSM lipids in the upper and lower leaflets (RBC-0%CHOL), and a model RBC membrane with the same asymmetric lipid distribution but with 50 mol % CHOL in both leaflets (RBC-50%CHOL). POPC, POPE, and POPS lipids contain both a palmitoyl (16:0) and oleoyl (18:1) tail, differing in their head groups (PC = phosphatidylcholine, PE = phosphatidylethanolamine, PS = phosphatidylserine). POSM contains the same headgroup and oleoyl tail as POPC but includes a sphingosine (d18:1) tail in place of the palmitoyl tail of POPC. Figure S1 shows the chemical structures of these species. Lipid distributions in the RBC membranes were chosen based on previous electron microscopy^42^ and mass spectrometry^31^ experiments that quantified mole fractions of lipids in human erythrocyte plasma membranes. To reflect RBC lipid asymmetry following previous CG MD studies of model RBC membranes using the MARTINI force field,^43–47^ we estimated lipid fractions for the RBC-0%CHOL membrane as follows: 45% POPC, 45% POSM, and 10% POPE in the upper leaflet and 20% POPC, 10% POSM, 45% POPE, and 25% POPS in the lower leaflet. Figure 1b shows the lipid fractions used for all four membranes in this study. All bilayers were initialized with the *insane* tool.^48^ The mapping and bead parameterization strategy followed the MARTINI 2.2 CG force field^49^ for all phospholipids and CHOL and is visualized in Figure S1. The total number of lipids in the upper leaflet for the RBC-0%CHOL and RBC-50%CHOL systems was decreased slightly to better match the area per lipid (APL) between the leaflets (Figures S2 and S3), as recommended for MD simulations to minimize the surface area difference (and therefore lateral tension) between the leaflets in asymmetric membranes.^50^

**Figure 1:**
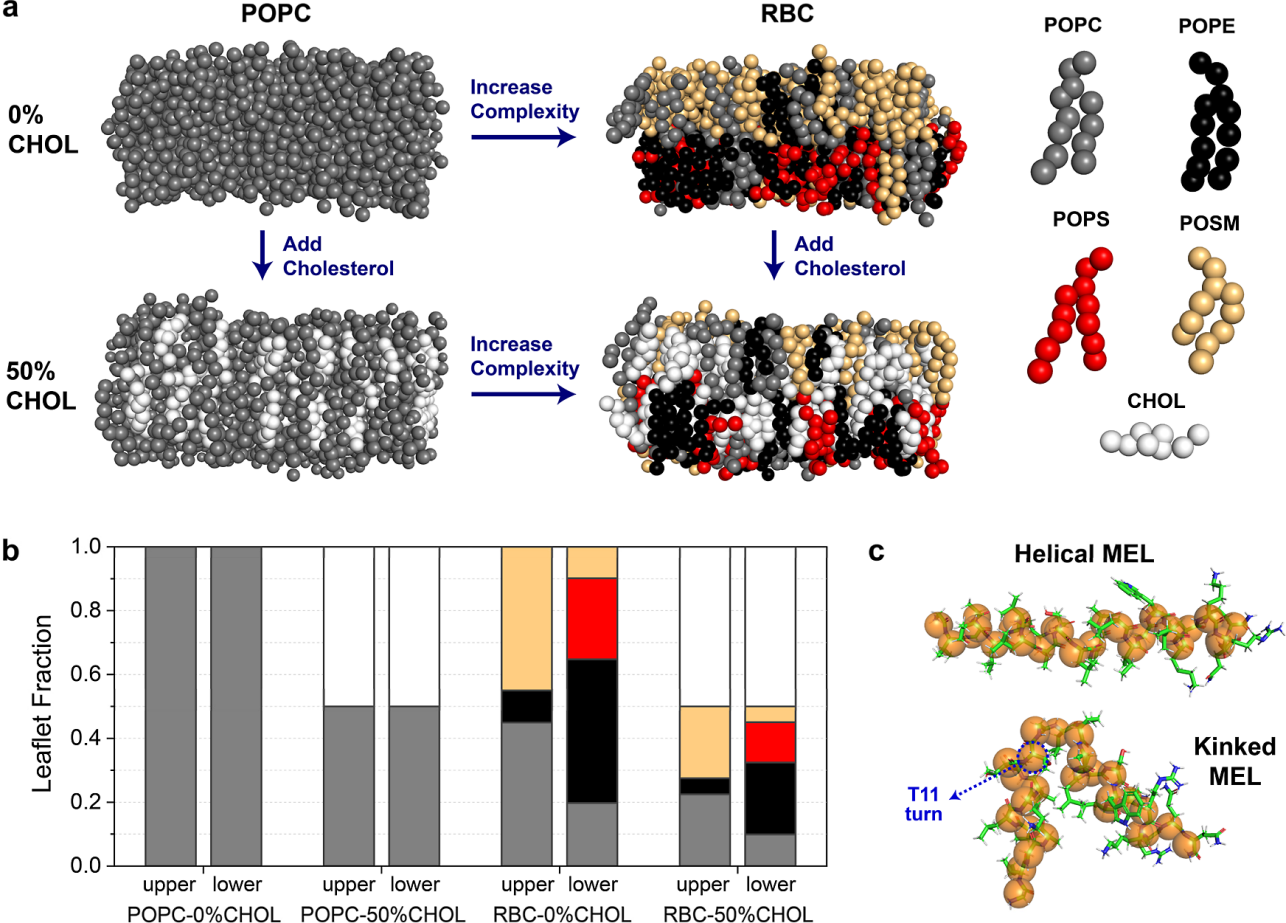
System preparation with the MARTINI 2.2 force field. (a) Four membrane compositions were considered with differing complexity and CHOL mol %: pure POPC (POPC-0%CHOL), POPC with 50 mol % CHOL in both leaflets (POPC-50%CHOL), a model RBC membrane with asymmetric distributions of POPC, POPE, POPS, and POSM (RBC-0%CHOL), and RBC with 50 mol % CHOL in both leaflets (RBC-50%CHOL). Membrane components are displayed as MARTINI beads. (b) Lipid leaflet mole fractions for all four membranes are plotted with the same color scheme as the membrane components in (a). (c) Snapshots showing the two MEL conformations. Atomistic representations are shown as sticks, and MARTINI beads for each amino acid backbone are overlaid as orange spheres.

Figure 1c shows the two conformations of MEL (helical and kinked) considered in this study. Atomistic structures were created using Avogadro,^51^ and mapped MARTINI structures were created with the *martinize* tool. Helical MEL parameters include 60° proper dihedrals with 400 kJ mol^–1^ force constants^49,52^ for all backbone beads to maintain an α-helical structure. Because the MARTINI protein secondary structure elements are fixed during MD simulations, helical MEL was chosen to match experimental evidence that MEL peptides fold into helices after binding to plasma membranes in their pore-forming mode of action, as explored in our past study.^29^ Kinked MEL was additionally included based on previous mechanistic studies that observed “U-shaped” pore lining by MEL during pore formation^38,39,53^ due to structural flexibility in the middle of the MEL sequence. In the crystal structure for tetrameric MEL (PDB ID: 2MLT), the *dssp* algorithm^54^ calculates that for chain A, there are two continuous α-helix segments (residues 2–10 and 12–25) linked by a flexible π-turn at T11 (for chain B, there is a single continuous α-helix from residues 2–25), where the G1 and Q26 terminal residues are random coils.^38,40^ Therefore, in parameterizing kinked MEL, proper dihedrals for G1, T11, and Q26 are removed compared to helical MEL, which is consistent with previous MD simulations that used the MARTINI 2.2 force field.^38^

### System Setup

As reported in our previous work,^29^ we utilized a four-step approach for preparing equilibrated MEL-membrane systems. For all steps, we used Gromacs 2021.5 patched with PLUMED 2.8.^55^ First, each membrane was assembled to span the *xy* area of the simulation box with a normal in the *z* direction, and a 2 × 4 grid of MEL peptides was initialized 2.5 nm above the *z*-component of the center of mass (COM) of the membrane (Figure S4a). Systems were solvated with at least 10 W beads (equivalent to 40 water molecules) per membrane component (lipids and CHOL) and a salt concentration of 150 mM NaCl using the *insane* tool.^48^ Next, energy minimization was performed using the steepest descent algorithm with a maximum step size of 0.01 nm and 100 kJ mol^–1^ nm^–1^ tolerance (Figure S4b). After energy minimization, two equilibration steps were followed. First, a bias was applied between the COM of each MEL peptide and the membrane COM that acts when the *z*-component of the COM of each MEL and the membrane COM exceeds 2 nm to promote MEL-membrane adsorption to the membrane upper leaflet. This bias was applied at *z* = 2 nm using the PLUMED “upper walls” command with 1000 kJ mol^–1^ harmonic restraints additionally applied to restrict motion in the *xy*-plane for MEL terminal backbone beads, thus preserving the initial grid arrangement (Figure S4c). Second, all biases from the first equilibration step were removed to allow for natural clustering of the MEL in the membrane (Figure S4d). Both equilibration steps were performed for 50 ns with a 0.02 ps time step. A temperature of 323 K was chosen to prevent freezing of MARTINI water P4 beads, and the dielectric constant was set to 15 as recommended for MARTINI 2.2.^56^ The temperature was controlled with a velocity-rescale thermostat^57^ with time constant of 1 ps, and the pressure was controlled at 1 bar with the Berendsen barostat^58^ with semi-isotropic pressure coupling with a 3 × 10^–4^ bar^–1^ compressibility and a 5 ps time constant. The Verlet cutoff scheme with a buffer tolerance of 0.005 kJ mol^–1^ ps^–1^ was implemented for neighbor searching. Lennard-Jones interactions were cut off at 1.1 nm, and the particle mesh Ewald (PME) method was used for electrostatic interactions with a short-range cutoff of 1.1 nm.

To standardize the membrane surface area for MEL-containing systems across the 4 membrane compositions (Figure 1a), we targeted a surface area of 100 nm^2^ (Figure S5) through adjustments to the total number of lipids in the two membrane leaflets while accounting for the differences in upper and lower leaflet APL due to lipid asymmetry in the RBC-0%CHOL and RBC-50%CHOL membranes (Figures S2 and S3). The total number of lipids, water beads, and ions for all MARTINI systems is listed in Table S1. To induce the formation of pores in lipid membranes, we utilized the nucleation collective variable (ξ) proposed by Hub and Awasthi^34^ to bias water (W) and lipid phosphate (PO4) beads^29^ in a sliced transmembrane cylinder to create a continuous polar defect. A value of ξ = 0.2 corresponds to a flat, unperturbed membrane, while a value of ξ = 1.0 corresponds to a fully nucleated transmembrane pore. The number of cylinder slices of a thickness of 0.2 nm that was used for the calculation of ξ was selected based on unbiased simulations of the four bare membranes to match ξ = 0.2 most closely (see Figure S6). Nineteen slices were used for 0% CHOL membranes, and 22 slices were used for 50% CHOL membranes. ξ was implemented as a custom collective variable in PLUMED, as utilized in our previous study.^29^

### Long-Time-Scale Pore Equilibration

In our previous work, steered MD was used to increase the value of ξ (i.e., pull) from ξ = 0.2 to ξ = 1.0 for equilibrated MEL-containing DMPC membranes to induce pore nucleation, and then 23 windows were extracted at different values of ξ and simulated independently for 500 ns each to generate MEL-lined pores of increasing size.^29^ This method worked well in generating realistic pore structures given the fast lateral diffusion of MEL in DMPC membranes. However, MEL diffusion in the CHOL-containing membranes studied in this work is much slower due to CHOL significantly lowering lipid mobility at 50 mol %.^59^ Therefore, we added an additional “Long-time-scale Pulling” step to ensure equilibrated MEL pore lining for membranes in this study.

Figure 2a shows example results from one such step in which a harmonic potential of 30,000 kJ mol^–1^ is maintained across the simulation length. First, steered MD is used to pull from ξ = 0.2 to ξ_long_ over 50 ns (i → ii in Figure 2a), where ξ_long_ is initially set to 0.7. The system is then restrained at ξ_long_ for 5 μs (ii → iii) to reach a total simulation time of 5.05 μs, which is denoted as “*t*_cutoff_” (red vertical line in Figure 2a). If pore lining is not observed by *t*_cutoff_, then the simulation is restarted from ξ = 0.2 (state i) and ξ_long_ is increased by 0.025. This procedure is repeated until MEL pore lining is observed at the *t*_cutoff_. A series of alternating 50 ns pulling and 500 ns simulation steps are then conducted for ξ values that are multiples of 0.1 (e.g., ξ = 0.8, 0.9) until full pore nucleation at ξ = 1.0 (iii → iv, Figure S4e). For systems where 0.7 ≤ ξ_long_ ≤ 0.775, this corresponds to a total simulation time of 6.2 μs (Figure 2a), and for systems where 0.8 ≤ ξ_long_ ≤ 0.875, this corresponds to a total simulation time of 5.65 μs (Figure S7). Simulation parameters are identical to the equilibration steps of the system setup, except that the Parrinello–Rahman barostat was used to control the pressure with a 12 ps time constant. We then extracted 23 umbrella sampling windows (11 windows from ξ = 0.2 to ξ = 0.7 in a 0.05 increment with a 10,000 kJ mol^–1^ force constant and 12 windows from ξ = 0.725 to ξ = 1.0 in a 0.025 increment with a 20,000 kJ mol^–1^ force constant) and simulated each window for an additional 500 ns with an umbrella potential applied (Figure S4f). In general, we find that this method leads to reasonable and repeatable MEL pore-lining behavior across three independent replicas for each membrane type. For bare membrane systems lacking peptides, this long-time-scale equilibration process was performed using the same value of ξ_long_ as selected in corresponding helical MEL systems and 23 windows were extracted and simulated for an additional 500 ns each.

**Figure 2:**
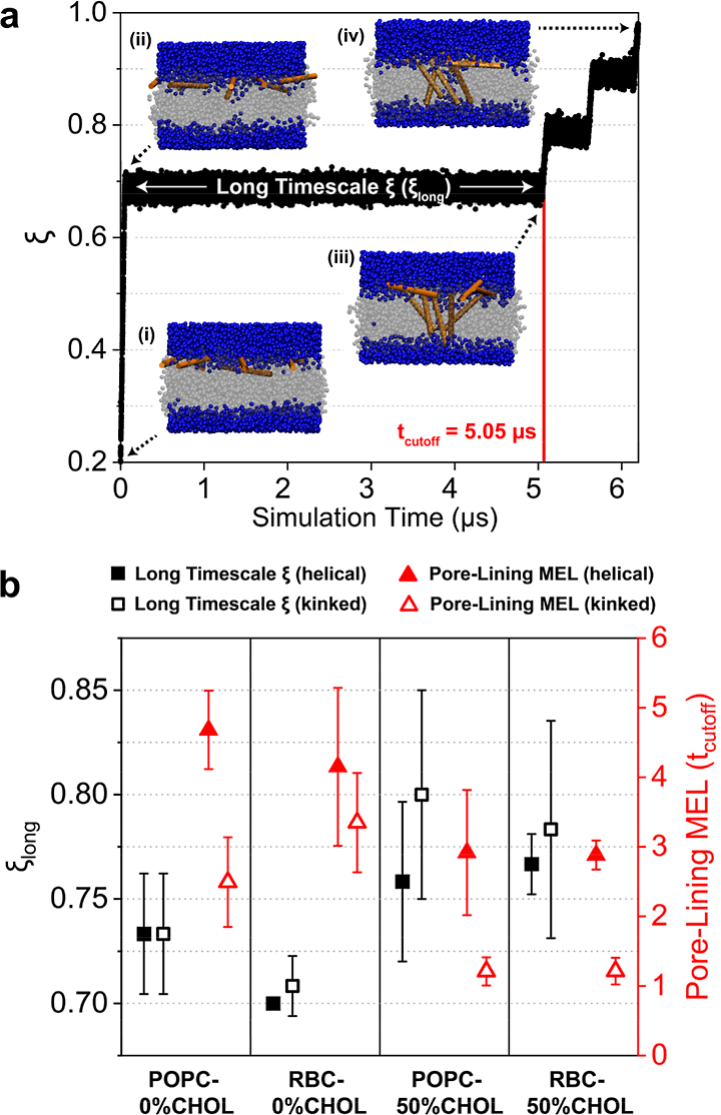
(a) Long-time-scale equilibration procedure. Simulation snapshots are displayed with the lipid membrane as gray spheres, water beads as blue spheres, and 8 MEL peptides as orange cartoons. Starting from a flat membrane state with MEL in the upper leaflet (i), ξ is increased to a pore size of ξ_long_ that is initially set as 0.7. The system is restrained at ξ_long_ for 5 μs (ii → iii). If MEL pore lining is not observed by *t*_cutoff_ = 5.05 μs, then the process is repeated from (i) and ξ_long_ is increased by 0.025. This process is repeated until at least one MEL lines the defect at the *t*_cutoff_. Lastly, ξ is increased by alternating 50 ns steered MD and 500 ns restrained equilibration steps until the full pore state with ξ = 1.0 is reached (iv). (b) Average ξ_long_ (black) and number of pore-lining MEL (red) from three independent replicas for the four membranes. Solid points indicate systems with helical MEL, and open points indicate systems with kinked MEL. Error bars indicate standard deviations across replicas.

### Backmapping from Coarse-Grained to All-Atom Resolution

To increase the resolution of MEL pore-lined configurations for free energy and additional trajectory analysis, we converted equilibrated MARTINI configurations to the atomistic CHARMM36 force field using an adaptation of the *backward* tool,^35^ which estimates atomic positions from MARTINI bead positions through local geometry reconstruction. MARTINI configurations were backmapped to AA systems compatible with the CHARMM36 force field through projection estimation of atom positions for all membrane, MEL, and solvent components (step 1 in Table S2).^35^ These initial positions are defined in mapping files, which also include entries for additional reconstruction of local geometry for specific chemical groups that may not be resolved correctly on the initial projection (e.g., cis vs trans isomerism for double bonds, chiral centers). Mapping files for all 20 amino acids are readily available for MEL and were used for backmapping in our previous study.^29^ The same atomistic force field parameters were used to describe systems backmapped from both helical and kinked MEL CG models (Figure 1c) because in the AA simulations, MEL can fluctuate between perfect α-helix and U-shaped conformations, as well as conformations corresponding to bent α-helical rods,^60^ and as such, different force field parameters were not needed to model different MEL conformations. For POPC, POPE, and POPS lipids, mapping files were updated for use with the MARTINI 2.2 force field because currently available mapping files rely on a 13-bead mapping strategy for these lipids^35^ instead of the updated 12-bead models used in MARTINI 2.2. The mapping file for POSM has been newly created for this study. All geometric considerations for these 4 lipids during the backmapping process are visualized in Figure S8. Membrane properties for single-lipid membrane simulations, APL (Figure S9a), membrane thickness (Figure S9b), and deuterium order parameter (Figure S10), are in good agreement when comparing atomistic to back-mapped atomistic systems. The backmapping file for CHOL was adjusted slightly from its original implementation^35^ to better recreate the 8 chiral centers of cholesterol (Figure S11). After backmapping, systems were energy-minimized and briefly equilibrated in a 10-step procedure detailed in the Supporting Information (Table S2). All five updated or newly created backmapping files are included as part of the Supporting Information.

### Atomistic Umbrella Sampling and Free Energy Profiles

Atomistic systems backmapped from the final MARTINI configurations obtained after the long-time-scale equilibration procedure were simulated for 50 ns per umbrella sampling window (Figure S4h; 11 windows from ξ = 0.2 to ξ = 0.7 with 0.05 increment and 5000 kJ mol^–1^ force constant and 12 windows from ξ = 0.725 to ξ = 1.0 with 0.025 increment and 10,000 kJ mol^–1^ force constant). Because the slice thickness used in the calculation of ξ is 0.1 nm for atomistic systems^29,34^ compared to the 0.2 nm slice thickness used for CG systems (Figure S6), we recalculated the number of slices used in the calculation of ξ using unbiased simulations of each bare membrane (Figure S12). For all simulations, the temperature was set to 310 K to match the average physiological temperature and was controlled with a velocity-rescale thermostat with a time constant of 1 ps. The Verlet cutoff scheme with a buffer tolerance of 0.005 kJ mol^–1^ ps^–1^ was implemented for neighbor searching. Lennard-Jones interactions were smoothly switched to zero from 1.0 to 1.2 nm, and the PME method was used for electrostatic interactions with a short-range cutoff of 1.2 nm. Pressure was controlled at 1 bar with the Parrinello–Rahman barostat with semi-isotropic pressure coupling with a 4.5 × 10^–5^ bar^–1^ compressibility and a 5 ps time constant. The first 10 ns of each simulation were discarded for equilibration, and simulation configurations were saved every 0.1 ns for analysis for the remaining 40 ns per window. Potentials of mean force (PMFs) for each system were then computed as a function of ξ using Grossfield’s implementation of the weighted histogram analysis method (WHAM).^61^ All simulations in this study were repeated to obtain a total of three independent replicas, each initiated with different starting configurations from the first step of the workflow.

### Membrane Area per Lipid and Thickness Calculations for Atomistic Systems

To calculate the area per lipid (APL), we first determined the average membrane area (which spans the *xy*-plane in the simulation box as shown in Figure S9a) by multiplying the time-averaged box lengths in the *x*-direction and *y*-direction. Average box lengths were calculated by using the *gmx energy* tool. The APL was then approximated by normalizing this average membrane area by the average number of lipids and CHOL in the upper leaflet (*N*_upper_) during the simulation. To compute the bilayer thickness for each system, we calculated the average density profile (in kg m^–3^) of lipid head phosphorus atoms with the *gmx density* tool using 200 slices in the *z*-direction and found the distance between the upper and lower membrane leaflet head phosphorus density peaks (Figure S9b).

## Results and Discussion

### The Pore-Lining Affinity of MEL in RBC-Mimetic Membranes Is Significantly Increased in the Absence of Cholesterol

As demonstrated in our previous work,^29^ the MARTINI force field is successful in increasing the lateral diffusion and dynamics of system species (both lipids and AMPs) to better access pore-nucleation time scales compared to atomistic simulations. Consequently, we found that 500 ns per window was sufficient to sample consistent pore-lining behavior for a variety of AMPs (including MEL) in DMPC membranes. In initial simulations of MEL in the four membranes considered in this study (Figure 1a), however, we found that 500 ns per window was insufficient for system preparation due to both the increase in membrane thickness resulting from lipids with longer tail lengths (POPC, POPE, POPS, POSM) compared to DMPC and the inclusion of CHOL, which has been shown to significantly decrease the lateral diffusion of lipids^59,62–64^ and embedded membrane proteins^64^ in previous MD studies.

Given these considerations, we implemented a long-time-scale equilibration step (Figure 2a) to incrementally sample MEL pore lining as a function of ξ before umbrella sampling. Starting from a flat membrane state with eight embedded MEL (state i in Figure 2a), we induced the formation of a membrane defect corresponding to the start of the transition state for pore nucleation (denoted as ξ_long_ in Figure 2a).^34^ This defect was then maintained for 5 μs to allow sufficient time for MEL diffusion and membrane insertion, given the stochastic nature of MEL pore lining, which requires several MEL peptides to collectively align their more hydrophobic N-termini (compared to their highly charged C-termini) toward a polar lipid membrane defect before achieving favorable MEL N-terminus–lipid tail interactions in the transmembrane pore-lining state.^27–29^ Additional equilibration steps were then implemented to allow for unbiased equilibration of MEL pore-lined configurations at larger pore sizes (states iii → iv in Figure 2a). Figure 2b shows the results of the equilibration procedure for each of the four membrane compositions (Figure 1a) and two MEL conformations (Figure 1c). Larger values of ξ_long_ indicate a larger defect size is required for pore lining. The average number of MEL lining a transmembrane defect (pore-lining MEL) at ξ_long_ was calculated based on the total MEL density in the central 1 nm of the pore at *t*_cutoff_ normalized by the density for 1 pore-lining MEL (see Section S5).^29^ Average values and standard deviations for both quantities are based on values computed for three independent replicas of the entire equilibration procedure.

When comparing MEL defect size in membranes with 0% CHOL, the average size of the defect required for pore lining in RBC membranes decreases compared to that in POPC. In RBC membranes, helical MEL rapidly and consistently line the defect at ξ_long_ = 0.7 for all three replicas (Table S3 and Figure S13), whereas POPC membranes require slightly larger defects on average (0.7 ≤ ξ_long_ ≤ 0.75) for similar pore-lining behavior (Table S3). Similar trends are observed for kinked MEL. We attribute the smaller ξ_long_ values in RBC membranes to electrostatic interactions between +6-charged MEL and anionic POPS lipids in the lower leaflet of the RBC membrane that favor defect formation. Similarly, previous CG simulations with the MARTINI force field have found a significant free energy decrease during translocation of a cationic polyarginine peptide from the zwitterionic upper leaflet to anionic lower leaflet of an asymmetric DMPC/DMPS bilayer.^65^ In our simulations, an increased preference of MEL for the anionic lower leaflet of RBC is evident from the presence of helical MEL in the lower leaflet of RBC membranes at the end of pore equilibration (ξ = 1.0) in two of the three replicas, whereas no MEL translocated to the lower leaflet in POPC membranes or in DMPC membranes in our previous work.^29^ For membranes with 50% CHOL, larger ξ_long_ values are required on average to observe pore lining compared to 0% CHOL for both helical and kinked MEL, although the effect is substantial only for RBC membranes (Figure 2b). We attribute the increase in ξ_long_ to the increased membrane thickness caused by CHOL (Figure S6). In the RBC-50%CHOL membrane, this increased thickness leads to diminished electrostatic interactions between MEL in the upper leaflet and POPS in the lower leaflet, which contributes to the large increase in ξ_long_ compared to RBC-0%CHOL. Supporting this argument, no MEL were observed to translocate to the lower leaflet for RBC-50%CHOL simulations across three replicas.

When the number of pore-lining peptides is compared for systems where more than one MEL lines the pore by *t*_cutoff_, pore lining by one MEL leads to rapid, collective lining by several MEL peptides (Figure S13). This behavior is consistent with previous MD studies that found that the N-terminus of MEL acts as a “defect sensor” which can stabilize a membrane defect caused by thermal fluctuations to allow for diffusion of several MEL peptides into the defect, provided a large enough concentration of bound MEL.^66^ We observe that there is consistently a higher number of pore-lining helical MEL compared to kinked MEL for all membranes. The number of MEL that line the pore at ξ_long_ is comparable for both RBC and POPC membranes with 0% CHOL, with 3–5 helical MEL and 2–4 kinked MEL lining the pore (Figure S13) by *t*_cutoff_. The number of pore-lining MEL decreases, on average, from 0% to 50% CHOL systems. The effect of CHOL is not due to a diffusional limitation of MEL associated with the effect of CHOL on decreasing lipid dynamics^59,62^ since pore-lining trends appeared to be consistent for both POPC-50%CHOL and RBC-50%CHOL (Figure S13). Instead, we note that, in general, the most favorable pore-lining configurations involve MEL fully spanning the transmembrane pore.^29^ In thicker membranes, such as those with CHOL, there is a conformational entropy penalty for MEL pore lining^38^ that constrains membrane-spanning MEL structures toward more ideal α-helices and inhibits pore lining. We also expect the same conformational entropy penalty to promote pore lining by helical MEL since they already adopt structures ideal for spanning the membrane compared to more flexible kinked MEL, which explains the increased number of helical vs kinked pore-lining MEL in both membranes. Additionally, we observe that over long μs time scales, initially kinked U-lining MEL can transition to transmembrane pore-lining configurations (Figure S14), suggesting that U-lining MEL could act as a precursor for eventual transmembrane toroidal-type MEL pores. We explore this behavior in more detail in the following sections.

### POPS Flip-Flop and Pore Enrichment Are Coupled with MEL Pore Lining

We next aimed to elucidate the relationship between the POPS dynamics and MEL pore lining in RBC membranes. As noted above, we hypothesized that MEL pore lining is coupled to strong electrostatic interactions with anionic POPS lipids in the lower leaflet of the asymmetric RBC membranes, and this effect is diminished by the membrane-thickening effect of CHOL. Figure 3 shows two metrics calculated during the long-time-scale equilibration simulations to quantify the flip-flop of POPS from the lower to upper leaflet due to MEL pore lining (“POPS Fraction in Upper Leaflet”, Figure 3a) and the enrichment of POPS in the local pore environment (“POPS within 2 nm of pore”, Figure 3b). Both metrics were calculated every 50 ns based on the positions of POPS PO4 beads. The fraction of POPS lipids in the upper leaflet was calculated by dividing the total number of POPS PO4 beads with *z*-coordinates larger than the membrane midplane (approximated by the average *z*-position of all lipid tail end C4A and C4B beads) by the total number of POPS lipids. Figure 3a shows the time step where the first POPS flips to the upper leaflet of a bare (i.e., no MEL) RBC-0%CHOL membrane, which corresponds to an upper leaflet POPS fraction of around 0.03. POPS lateral enrichment was calculated by counting the total number of POPS PO4 beads within 2 nm of the pore center (set as *x* = 5 nm, *y* = 5 nm in our calculation of ξ). Figure 3b visualizes the radial region used in this calculation, where five POPS lipids are within 2 nm of the pore center for a bare RBC-0%CHOL membrane.

**Figure 3:**
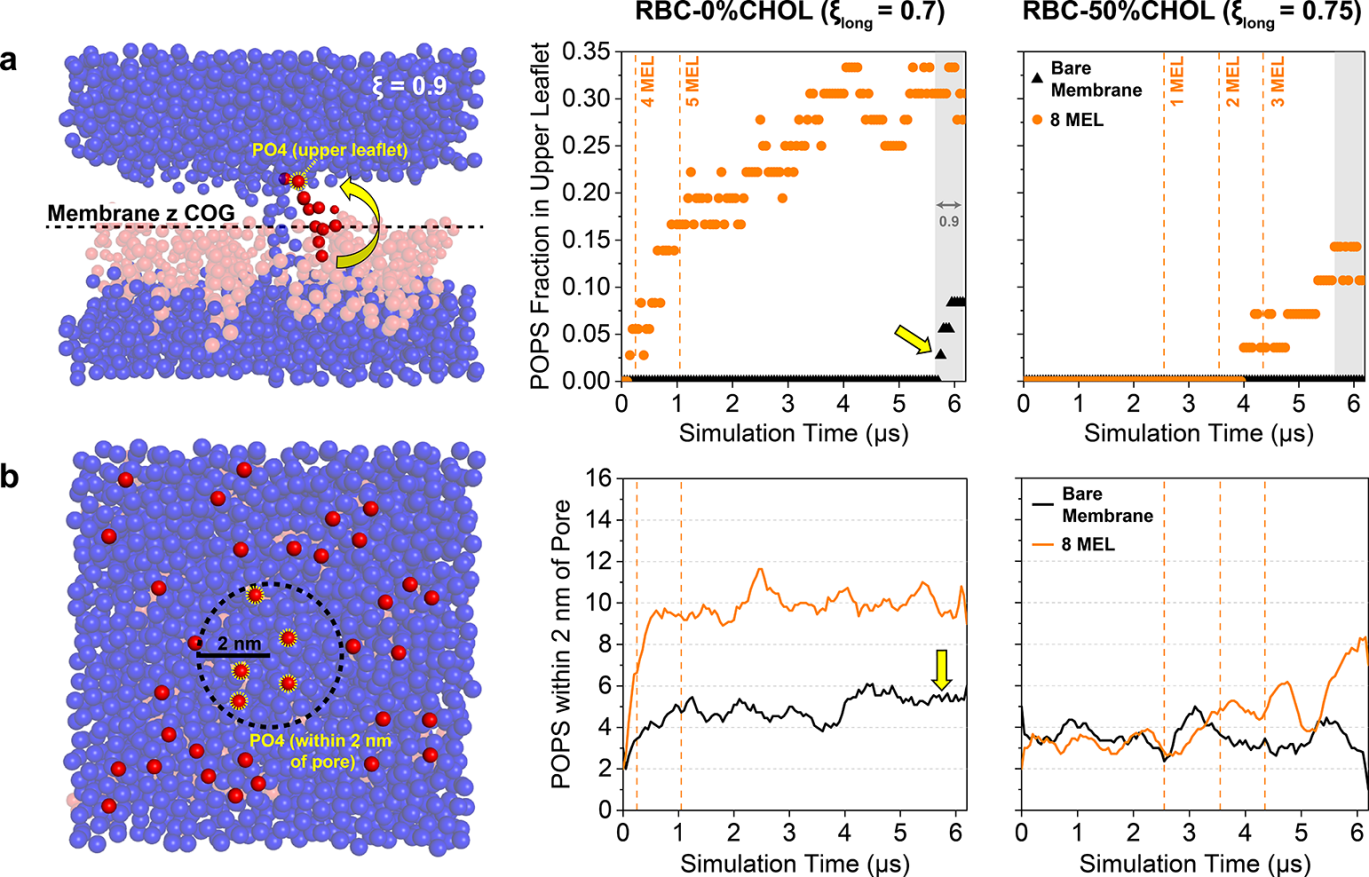
Quantification of POPS flip-flop and enrichment near the pore. (a) Calculation of the POPS fraction in the upper leaflet to quantify flip-flop in RBC membranes. The snapshot visualizes the first POPS that flips to the upper leaflet by crossing the *z* center of geometry (COG) as opaque red beads and labels the membrane *z* COG. Other POPS are shown in the lower leaflet as transparent red beads. Water beads (W) are shown as blue spheres, and the pore size corresponds to ξ = 0.9. Plots show calculated POPS fractions in the upper leaflet for RBC-0%CHOL with ξ_long_ = 0.7 and RBC-50%CHOL with ξ_long_ = 0.75, with the helical MEL system plotted in orange and the corresponding bare membrane system in black. Vertical orange dashed lines denote the lining of MEL peptides as calculated in Figure S13. The gray regions indicate the onset of flip-flop in the bare membranes at a larger value of ξ = 0.9. (b) Calculation of the number of POPS within 2 nm of the pore. The snapshot visualizes the region within 2 nm of the pore (black dashed circle) with POPS lipids within this region circled in yellow and all POPS PO4 beads shown in red. Plots show the number of POPS lipids within 2 nm of the pore with the same color coding as (a). Values were smoothed using adjacent averaging with 10 points. Yellow arrows in (a,b) correspond to the same time step in the RBC-0%CHOL system, which was used to generate the snapshots at left.

To directly compare the effect of both MEL and CHOL on POPS flip-flop and lateral enrichment, Figure 3 shows the POPS fraction in the upper leaflet and the number of POPS within 2 nm of the pore center for one of the three replicas of an RBC-0%CHOL membrane with ξ_long_ = 0.7 and an RBC-50%CHOL membrane with ξ_long_ = 0.75. Results for membranes with helical MEL are compared to corresponding bare membranes, and all three replicas are plotted in Figures S15 and S16. In general, MEL substantially increases the flip-flop and lateral enrichment of POPS in RBC membranes without CHOL (RBC-0%CHOL) compared to bare RBC membranes. The start of the POPS flip-flop is coupled with five MEL cooperatively lining the transmembrane defect, and the helical MEL system equilibrates to an upper POPS fraction of approximately 0.3 at ξ_long_ = 0.7. Additionally, the lining of several MEL doubles the number of POPS lipids within 2 nm of the pore, indicating preferential MEL-POPS interactions. Without MEL, a significantly increased pore size (ξ_long_ ≥ 0.9) is required to observe POPS flip-flop in bare RBC membranes (Figure 3a). Although POPS lipids present in the lower leaflet of mammalian membranes do not typically result in significant attraction to cationic AMPs in solution or in membrane-bound states,^43,67,68^ these results suggest that MEL-POPS interactions promote the ability of MEL to induce membrane defects,^29,66,69^ particularly in thin phospholipid membranes.^60,70^

For RBC membranes with 50% CHOL, the ability of MEL to induce POPS flip-flop and enrichment around membrane pores is substantially reduced compared to 0% CHOL. No POPS flip-flop is observed in the absence of MEL, and 2–3 pore-lining MEL are required to initiate POPS flip-flop at an increased pore size of ξ_long_ = 0.75 compared to ξ_long_ = 0.7 for the RBC-0%CHOL system (Figure 3a). The number of POPS within 2 nm of the pore is comparable between the bare membrane and membranes with MEL, although POPS may become slightly enriched at larger pore sizes in the presence of MEL (Figure 3b). These results are consistent with the inhibitory effect of CHOL on the ability of membrane-spanning peptides to promote lipid flip-flop, which is well documented with experimental flip assays utilizing C6-NBD fluorescent lipid tags.^71–73^ We attribute the decreased POPS flip-flop rate in RBC-50%CHOL membranes to the increased distance between leaflets that each POPS PO4 bead has to travel through the defect and the increased membrane mechanical rigidity resulting from greater lipid tail order in the presence of CHOL,^59,64,73^ thereby decreasing the thermodynamic favorability of POPS flip-flop (even in pores lined by up to 3 MEL, Figure S13).

### Helical MEL Preferentially Lines Membrane Pores

We next sought to compute potentials of mean force (PMFs) for pore nucleation as a function of membrane composition and MEL secondary structure to relate the observed differences in MEL pore lining and POPS dynamics. For these calculations, we leveraged the backmapping procedure developed in our prior work^29^ to convert the CG simulation configurations obtained from the long-time-scale equilibration procedure to atomistic resolution in order to calculate more reliable PMFs because the MARTINI force field tends to overestimate pore nucleation free energies as a result of higher bending moduli and line tensions compared to atomistic representations.^29,74^ Additionally, we find that MARTINI is unable to resolve differences in pore nucleation free energies as a function of lipid headgroup and tails (Figure S17). PMF profiles were computed as a function of the cumulative simulation time per umbrella sampling window to assess PMF convergence. Figure S18 demonstrates that only 10 ns of sampling per window is typically sufficient for bare membrane systems, and all MEL-containing systems converge within 40 ns of sampling per window (Figures S19 and S20). These results support the acceleration in PMF convergence achieved by our long-time-scale CG equilibration methodology to pre-position MEL pore-lining configurations prior to backmapping, which allows for higher umbrella sampling simulation throughput than is attainable with atomistic-only workflows.^28,75,76^

To establish the impact of MEL secondary structure, Figure 4a shows average PMFs for the POPC-0%CHOL membrane for systems with no MEL (bare membrane), 8 helical MEL, and 8 kinked MEL. The bare membrane PMF has a minimum around ξ = 0.2 (corresponding to the value of ξ for a flat, unperturbed membrane^29,34^ ), indicating correct selection of the number of transmembrane cylinder slices for the POPC membrane without CHOL (Figure S12). Relative to this state, pore nucleation (ξ = 1.0) in the bare membrane requires a free energy of 88.9 ± 1.8 kJ/mol, which is consistent with previous MD studies implementing ξ with the CHARMM36 force field.^74,77,78^ In membranes with MEL, values of ξ corresponding to PMF minima are increased slightly from ξ = 0.2, presumably due to the membrane-thinning effect of MEL adsorbed to the membrane (vide infra). The “transition point” labeled in both the helical and kinked MEL PMFs indicates the beginning of MEL pore lining at an average value of ξ = 0.73 for both MEL-containing systems (Figure 2b), and we observed similar behavior for MEL in DMPC membranes in our previous study.^29^ This transition point and the subsequent decrease in the PMF slope for higher values of ξ are indicative of pore-lining MEL lowering the pore line tension to favor pore nucleation.^26,27,29,79^

**Figure 4:**
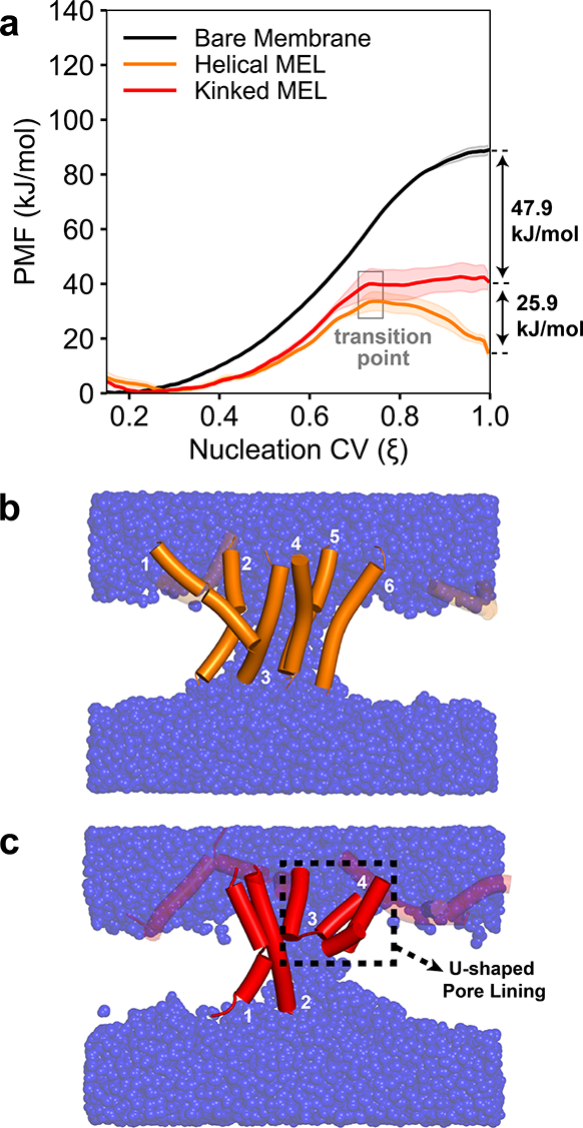
Free energies and pore-lining behavior of MEL in the POPC-0%CHOL membrane. (a) Potentials of mean force (PMFs) for the bare membrane, helical MEL, and kinked MEL systems with standard errors across three independent replicas indicated by shaded regions. The transition points that denote the beginning of MEL pore lining are boxed in light gray. PMF differences at ξ = 1.0 are shown with arrows. Analysis of PMF convergence is shown in Figures S18–S20. (b,c) Representative final pore structures at ξ = 1.0 for the POPC-0%CHOL membrane with (b) helical and (c) kinked MEL. Water molecules are shown as transparent blue spheres with helical MEL as orange cartoons and kinked MEL as red cartoons. Lipids are not shown for visual clarity. Pore-lining MEL are captioned in white. The two U-shaped kinked MEL are boxed in black in (c).

Interestingly, as shown in Figure 4a, the fully nucleated pore (ξ = 1.0) corresponds to a metastable free energy minimum for membranes with helical but not kinked MEL, suggesting increased stability of pore structures lined by MEL with fully α-helical secondary structures. Both kinked and helical MEL substantially decrease the free energy of the fully nucleated pore compared to the bare membrane: kinked MEL decreases the free energy by 47.9 ± 3.7 kJ/mol, and helical MEL decreases the free energy by a further 25.9 ± 3.8 kJ/mol (an additional 54% decrease relative to the bare membrane). This decrease is consistent across the three other membrane compositions considered in this study (Figure S21), with helical MEL consistently decreasing the free energy for pore nucleation by 27–39% more than kinked MEL compared to bare membranes.

Figure 4b,c shows simulation snapshots illustrating that helical MEL adopt fully transmembrane pore-lining conformations (Figure 4b), whereas kinked MEL adopt a mixture of transmembrane and U-shaped pore-lining conformations (Figure 4c). Previous MD simulations have likewise found that MEL can preferentially insert into membranes in a U-shaped conformation when modeled with a flexible kink, which is attributed to the larger conformational entropy of U-shaped versus transmembrane conformations.^38,39^ U-shaped MEL may then act as a “wedge” to facilitate further insertion by transmembrane MEL,^53^ which appears to be consistent across all membranes with kinked MEL in this study, where fully nucleated pores are lined by both U-shaped and transmembrane conformations (Figure 4c and Table S4). Nonetheless, the U-shaped conformation of MEL has not been observed experimentally.^80^ Instead, experimental results support the toroidal pore formation mechanism for MEL based on oriented circular dichroism and neutron scattering experiments,^26,81^ where several α-helical MEL collectively insert and line a transmembrane pore. The decreased free energy for pore nucleation by helical MEL compared to that by kinked MEL agrees with these experimental observations, which suggests that the U-shaped pore-lining conformation may be metastable and relaxes to a transmembrane conformation over longer times. Analysis of long-time-scale MARTINI equilibration simulations for kinked MEL systems also indicates that initially U-shaped pore-lining MEL transition to transmembrane configurations over μs time scales (Figure S14). We therefore chose to focus our remaining analysis of atomistic free energies and pore structures on membranes with helical MEL.

### MEL Significantly Reduces Barriers for Pore Nucleation in All Membranes

Figure 5 shows average PMFs for all bare membrane and helical MEL systems based on their CHOL composition. None of the bare membranes considered exhibit a free energy minimum for the fully nucleated pore at ξ = 1.0 (which would suggest the potential for long-lived metastable pores). The increase in free energy at full nucleation for POPC membranes compared to DMPC membranes^29,34^ is expected, given the membrane-thickening effect of longer POPC lipid tails,^82^ and the lack of metastability at ξ = 1.0 is consistent with previous literature utilizing the CHARMM36 force field.^74,77,78^ The addition of POPE, POPS, and POSM lipids to the RBC membranes further increases their thickness and decreases their APL (Figure S9), thereby increasing the free energy for fully nucleated pores compared with POPC by 34.3 ± 4.4 kJ/mol. The addition of 50% CHOL to POPC and RBC membranes roughly doubles the PMF at ξ = 1.0 compared to equivalent membranes in the absence of CHOL by increasing lipid tail order.^64,83^ Through additional 500 ns unbiased simulations (Figure S22), we quantified the area compressibility modulus (*K*_A_), with higher *K*_A_ values indicating an increased ability of a bilayer to resist applied stress. Indeed, membranes with 50% CHOL have substantially higher calculated *K*_A_ (POPC = 1110 mN/m, RBC = 1233 mN/m) values than membranes with 0% CHOL (POPC = 214 mN/m, RBC = 322 mN/m). Additionally, we find a 50% increase in *K*_A_ from POPC-0%CHOL to RBC-0%CHOL membranes but only an 11% increase from POPC-50%CHOL to RBC-50%CHOL membranes. These rank orderings in *K*_A_ values are in good agreement with bare membrane free energies at ξ = 1.0 and indicate that added POPE, POPS, and POSM lipids more significantly increase the lipid membrane’s resistance to pore nucleation in the absence of CHOL.

**Figure 5:**
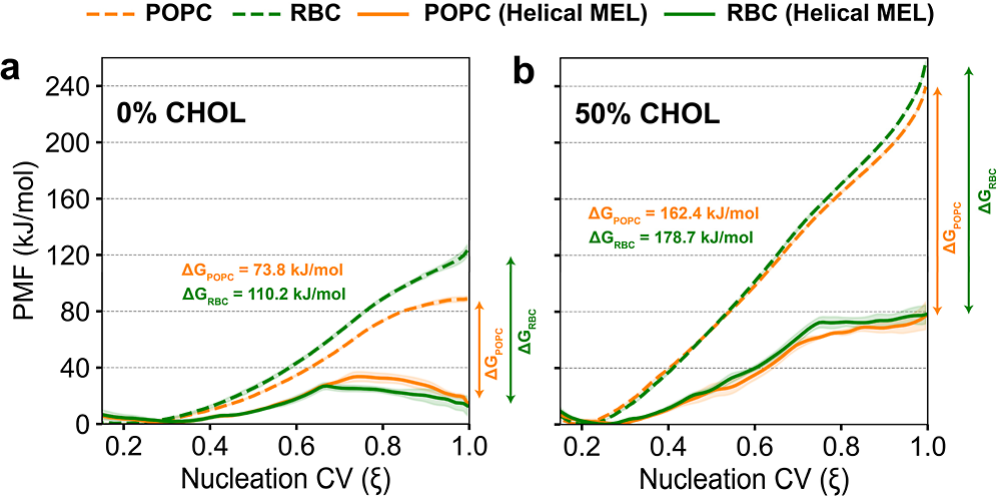
PMFs for (a) 0% CHOL vs (b) 50% CHOL systems. Results for POPC membranes are plotted in orange, and results for RBC membranes are plotted in green, with dashed lines for bare membranes and solid lines for systems with helical MEL. Shaded regions correspond to the standard error across three replicas. Differences in PMF values between bare membranes and helical MEL systems at ξ = 1.0 (Δ*G*) are shown as arrows with values inset on each plot.

Consistent with analysis in POPC-0%CHOL and our previous work in DMPC membranes,^29^ the PMFs in Figure 5 indicate that helical MEL significantly decreases the free energy for the fully nucleated pore state compared to all bare membranes. As ξ biases only water molecules and lipid head phosphates toward the transmembrane pore, decreases in free energies for MEL-containing systems compared to their bare membrane equivalents are a direct consequence of MEL pore lining, which alleviates lipid bending penalties through favorable peptide–water interactions. Binding of MEL and subsequent pore nucleation also decreases *K*_A_ (Figure S23), suggesting that MEL has a compounding effect on lowering *K*_A_ during aggregation and pore lining to favor pore nucleation compared to bare membranes (Figure 5). Transition points in these PMFs are observed at values of ξ equal to the ξ_long_ values calculated for each helical MEL system, which denote the smallest membrane defect size that could accommodate MEL during the long-time-scale equilibration. We note two key features of these PMFs: (1) MEL in 0% CHOL membranes causes a larger decrease in the PMF at ξ = 1.0 compared to bare membranes for RBC (Δ*G*_RBC_ = 110.2 ± 8.0 kJ/mol) vs POPC (Δ*G*_POPC_ = 73.8 ± 2.7 kJ/mol) despite a similar number of pore-lining MEL (Table S4), and (2) MEL-lined pores have higher free energies and lack metastability in membranes with 50% CHOL compared to membranes with 0% CHOL. These observations motivate additional analysis of the umbrella sampling trajectories in the following sections.

### MEL More Easily Disrupts RBC Membranes Compared to POPC Membranes

In classical pore formation theory in lipid membranes, cationic AMPs bound to the upper leaflet create a charge imbalance that induces an electric field through the hydrophobic core of the bilayer.^70,84,85^ This charge imbalance coupled with an area imbalance between leaflets (due to AMPs increasing the APL of the upper leaflet^60,70^ ) drives the formation of AMP-lined pores in lipid membranes at a sufficient peptide/lipid ratio. Therefore, to relate PMF trends to the membrane-disrupting effects of bound MEL prior to pore formation (i.e., for ξ = 0.2), we calculated the APL and bilayer thickness in both the absence and presence of MEL as shown in Figure 6. Both metrics were averaged across three replicas of the 40 ns umbrella sampling trajectory for the ξ = 0.2 window.

**Figure 6:**
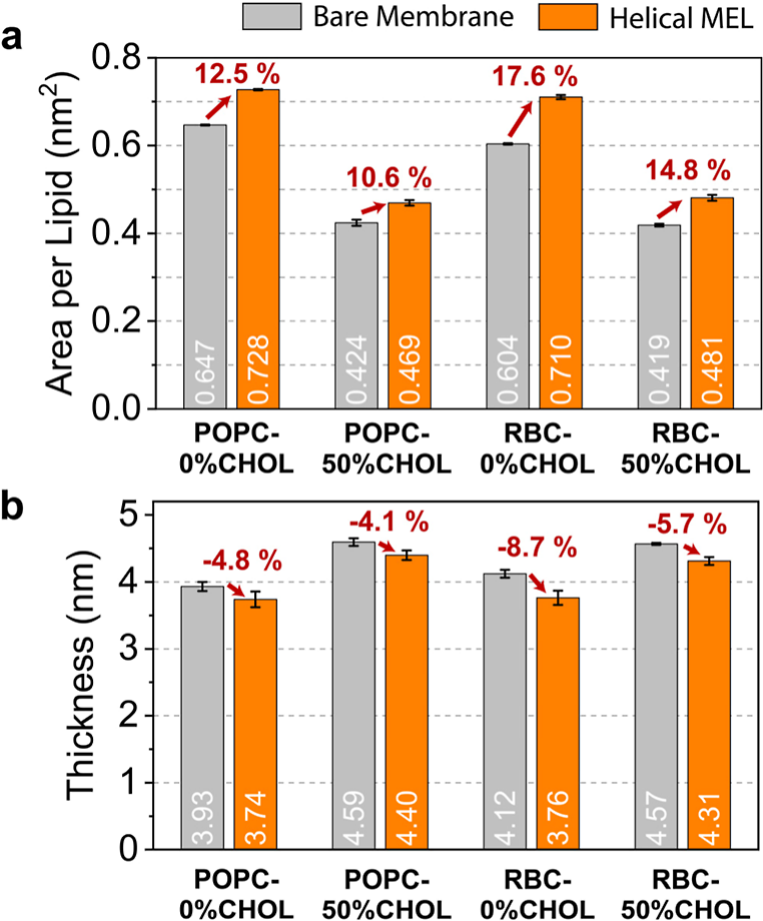
Average (a) APL and (b) bilayer thickness for each system restrained at ξ = 0.2 are plotted for membranes in the absence (gray) and presence (orange) of helical MEL. Red arrows indicate the (a) percent increase in average APL and (b) percent decrease in average thickness when MEL are present. Schematics visualizing the calculation of the APL and bilayer thickness are shown in Figure S9. Error bars indicate standard deviations across replicas.

In a comparison of APLs for bare membranes, there is a 6.6% decrease in the APL from POPC to RBC for 0% CHOL membranes but only a 1.2% difference from POPC to RBC for 50% CHOL membranes. These observations are consistent with differences in *K*_A_ values of these membranes (Figure S22) and PMF slopes starting from the flat membrane state (ξ = 0.2) in Figure 5, further supporting the increased importance of CHOL to resist membrane deformation compared to the addition of lipids with inherently lower APL than POPC (POPE, POPS, POSM in Figure S9a). Surprisingly, despite this decreased APL for RBC-0%CHOL vs POPC-0%CHOL membranes, the addition of membrane-bound MEL more substantially increases the APL relative to bare membrane simulations for RBC compared to POPC membranes for both the 0% CHOL (17.6% vs 12.5%) and 50% CHOL (14.8% vs 10.6%) cases. These results explain the increased value of Δ*G*_RBC_ compared to Δ*G*_POPC_ in Figure 5, suggesting that more favorable pore nucleation by MEL in RBC vs POPC membranes can partially be explained by the increased area imbalance in RBC membranes prior to pore nucleation. This behavior is because MEL effectively destabilizes tight packing of POSM lipids (Figure S9a)—due to an additional 4,5-trans double bond and an increased number of hydrogen bonding sites compared to POPC^86,87^ —which are enriched in the upper leaflet of RBC membranes.

To relate lateral expansion induced by MEL to bilayer thinning,^27,60^ we next computed the bilayer thickness for each system (Figure 6b) for each umbrella sampling trajectory restrained at ξ = 0.2. Like our observations of APL, there is a substantial difference in the bilayer thickness of bare 0% CHOL membranes (POPC = 3.93 ± 0.07 nm, RBC = 4.12 ± 0.06 nm) but not for 50% CHOL membranes (POPC = 4.59 ± 0.06 nm, RBC = 4.57 ± 0.02 nm), suggesting that CHOL has a similar ordering effect on lipid tails regardless of the lipid type. All bilayers see a decrease in average thickness in the presence of MEL, and rank orderings of this decrease (red arrows in Figure 6b) are equivalent to increases in APL (red arrows in Figure 6a). Additionally, MEL more readily thins RBC vs POPC membranes irrespective of CHOL content, suggesting that an increased electrostatic potential across the membrane^60,70^ due to the presence of anionic POPS lipids in the lower leaflet may contribute to more favorable bilayer perturbations and MEL insertion compared to POPC (Δ*G* values in Figure 5).

### CHOL Restricts MEL Conformations Lining Membrane Pores

Results in the prior sections show that MEL consistently disrupts lipid membranes in a manner that is favorable for pore nucleation, regardless of lipid and CHOL composition. Although lipid asymmetry and CHOL content both affected minimum pore sizes required for MEL pore lining (ξ_long_ in Figure 2b), the average number of pore-lining MEL at full nucleation is affected primarily by CHOL content in both RBC and POPC membranes. We next sought to better understand MEL pore lining to relate to CHOL content through the calculation of tilt angles for pore-lining MEL. Following our previous study on MEL in DMPC membranes,^29^ the tilt angle for each MEL was calculated as the average angle between the vector connecting the N-terminus backbone nitrogen and C-terminus backbone carbon in MEL and the *xy*-plane of the membrane. Tilt angle values range from 0° (parallel to the flat membrane and not pore-lining) to 90° (fully pore-lining).

Figure 7 shows average tilt angles of each pore-lining MEL for the ξ = 1.0 umbrella sampling window along with visualizations of MEL-lined pores for one replica of each system. Tilt angles demonstrate excellent convergence for all systems and replicas (Figure S24) and for varying pore sizes (Figure S25), further indicating that umbrella sampling windows with MEL pore-lining configurations are well equilibrated (Figures S19 and S20). The inclusion of 50% CHOL leads to a statistically significant (*p* < 0.05; see Table S5) increase in the average tilt angle of pore-lining MEL (63.0° to 70.7° in POPC membranes and 63.9° to 73.5° in RBC membranes). We attribute this behavior to the membrane-thickening effect of CHOL (Figure 6b), which restricts the conformational freedom of MEL and thereby increases the entropic penalty for MEL^38^ to line pores spanning the upper to lower leaflet. Indeed, we find decreased MEL pore lining for 50% CHOL membranes both in long-time-scale CG simulations (Figure 2b) and for AA umbrella sampling simulations at ξ = 1.0 (Table S4). These observations support the increased free energy at full nucleation for 50% CHOL versus 0% CHOL membranes (Figure 5). Interestingly, tilt angle averages are similar when comparing 0% CHOL (POPC = 63.0°, RBC = 63.9°) and 50% CHOL (POPC = 70.7°, RBC = 73.5°) membranes, which is consistent with comparable free energies at ξ = 1.0 for membranes containing MEL. However, if one partially lined MEL for replicas two and three for both 0% CHOL membranes (MEL with tilt angles <50° in Figure 7a, see Figure S26) is disregarded, it appears that MEL has an increased range of tilt angles in RBC (55–80°) versus POPC (60–76°) membranes. This suggests that there is a lower entropic penalty and higher conformational flexibility for MEL to line RBC pores compared to POPC in the absence of CHOL, which could contribute to the larger decrease in free energy at ξ = 1.0 for MEL-containing RBC vs POPC membranes (Δ*G*_RBC_ = 110.2 kJ/mol vs Δ*G*_POPC_ = 73.8 kJ/mol in Figure 5) despite thicker RBC membranes. We attribute this behavior to POPS lipids in both the upper and lower leaflet in RBC-0%CHOL membranes (due to POPS flip-flop in the presence of MEL, see Figure 3a), which help to stabilize the positively charged N- and C-termini of MEL. This increased affinity of cationic MEL to anionic POPS lipids enriched in both leaflets for RBC-0%CHOL membranes is consistent with previous MD free energy studies exploring the increased favorability of charged polyarginines for anionic compared to zwitterionic lipids.^65^

**Figure 7:**
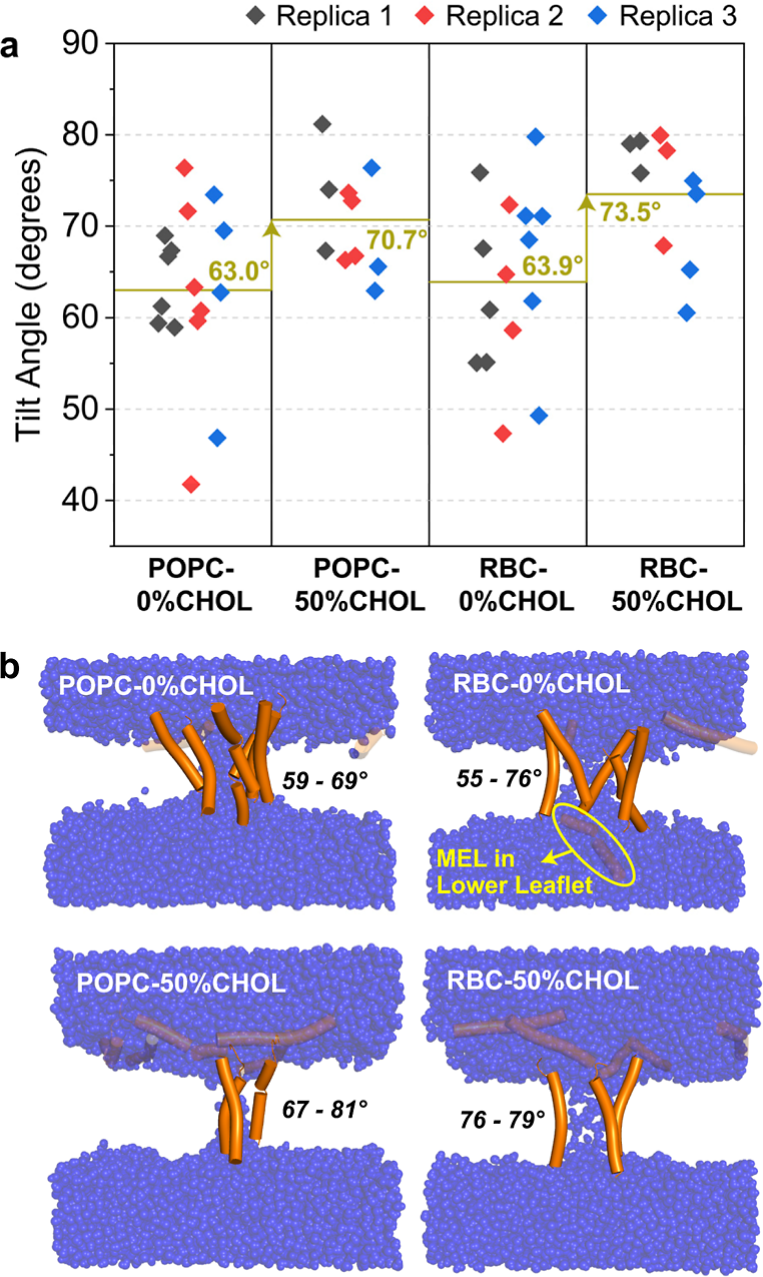
Analysis of pore-lining MEL tilt angles. (a) Tilt angle distributions for pore-lining MEL for three replicas for each of the four membranes. Average tilt angles for each membrane are plotted as horizontal gold bars. Arrows signify a statistically significant (Table S5) increase in average MEL tilt angle in the presence of 50% CHOL. (b) Representative simulation snapshots of pore-lining MEL (following the color scheme in Figure 4b). MEL that are not lining the pore are transparent. Snapshots correspond to the final configuration of the ξ = 1.0 umbrella sampling window. Tilt angle ranges for pore-lining MEL are labeled in black. A MEL that flipped to the lower leaflet of the RBC-0%CHOL system is circled in yellow.

Figure 7b shows the final MEL-lined configurations for replica 1 of the ξ = 1.0 umbrella sampling window for each of the four membranes considered (replicas 2 and 3 are depicted in Figure S26). These visualizations illustrate the increase in the average tilt angle and the decrease in the number of pore-lining MEL for 50% CHOL membranes compared to 0% CHOL membranes. The presence of MEL in the lower leaflet suggests that the electrostatic effect of lower leaflet POPS is strong enough to overcome MEL C-terminus anchoring in the upper leaflet^27,88^ at the early stages of pore nucleation in the RBC-0%CHOL membrane, which is not observed for the other three membranes. We also calculated the average number of water molecules in the middle 1 nm of the pore, as shown in Figure S27. Based on the definition of ξ with 0.1 nm thick transmembrane slices (Figure S12),^34^ a value of ξ = 1 is roughly equal to 30 water molecules in the middle 1 nm of the transmembrane pore. We found that MEL creates notably larger pores in the POPC membrane (74 waters, see Figure S27) compared to the RBC membrane (56 waters), which is consistent with the decreased free energy of nucleated pores. From visual observation, the presence of MEL in the lower leaflet both decreases the number of pore-lining MEL and pore size at ξ = 1, suggesting that RBC asymmetry can also work to inhibit the creation of classically toroidal MEL-lined pores compared to fully zwitterionic membranes.^26,28,29,60^ Conversely, similar numbers of pore-lining MEL (3–4 peptides, Figures 7b and S26), pore sizes (31–32 water molecules, Figure S27), MEL tilt angle ranges (Figure 7a), and lack of MEL in the lower leaflet for POPC and RBC membranes with 50% CHOL further support that the electrostatic effect of POPS is significantly diminished in membranes containing large amounts of CHOL, therefore leading to similar pore nucleation of MEL in POPC-50%CHOL and RBC-50%CHOL membranes (Figure 5b).

### RBC Pores Enrich POPS and Deplete POSM in the Presence of MEL

To better understand the change in the local lipid environment of RBC membranes due to pore nucleation, we next calculated the average fraction of POPC, POPE, POPS, and POSM lipids within a radius *r* of the pore (computed as the lateral distance from the pore center in the *xy*-plane of the simulation box) from the atomistic umbrella sampling simulations. The lipid fraction is calculated by normalizing the average number of head phosphate P atoms for each lipid by the average total number of lipid head phosphate P atoms within *r* for each system. The value of *r* was set for each system such that an average of 30 total lipids were within *r* for the fully nucleated pore state (ξ = 1), leading to *r* = 1.6 nm for the RBC-0%CHOL bare membrane, *r* = 2 nm for the RBC-0%CHOL membrane with 8 MEL, *r* = 2 nm for the RBC-50%CHOL bare membrane, and *r* = 2.2 nm for the RBC-50%CHOL membrane with 8 MEL. For both RBC-0%CHOL and RBC-50%CHOL membranes, we considered bare membranes without a pore (ξ = 0.2), bare membranes with a fully nucleated pore (ξ = 1.0), and MEL-containing membranes with a fully nucleated pore.

Figure 8 shows that for membrane pores containing MEL, there is a large enrichment of POPS (108–311% increase around the pore) and depletion of POSM (21–31% decrease) for MEL-containing vs bare membranes. The increased POPS enrichment near the pore for MEL-containing RBC-0%CHOL membranes (311%) compared to that for RBC-50%CHOL membranes (108%) is consistent with the initial CG simulations (Figure 3b) and further supports stronger MEL-POPS electrostatic effects when CHOL is absent to decrease the free energy in fully nucleated RBC pores (Figure 5a). This mechanism appears to be unique to asymmetric membranes like RBC, as anionic lipids in the upper leaflet of membranes have been shown to inhibit MEL translocation compared to fully zwitterionic membranes.^89–91^ In bare RBC membranes, POSM is enriched near the pore during pore nucleation (regardless of CHOL content) due to the strong hydrogen bonding capability of sphingomyelin lipids such as POSM resulting from the presence of both hydrogen-bond acceptors and donors in the sphingosine backbone that can interact favorably with pore water molecules.^86,87,92^ The depletion of POSM near the pore for MEL-containing membranes compared to bare membranes suggests that pore-lining MEL adequately interface with pore water with sufficient polar side chain–water interactions,^27,29,60^ thereby decreasing the bending penalty for POSM pore lining. This result is notable because the presence of an additional lipid-stiffening trans double bond in POSM opposes bending^92^ more strongly than the other 3 lipids in this study, indicating that the displacement of POSM increases the thermodynamic favorability of MEL-lined pores in RBC membranes compared to their bare membrane equivalents (Figure 5).

**Figure 8:**
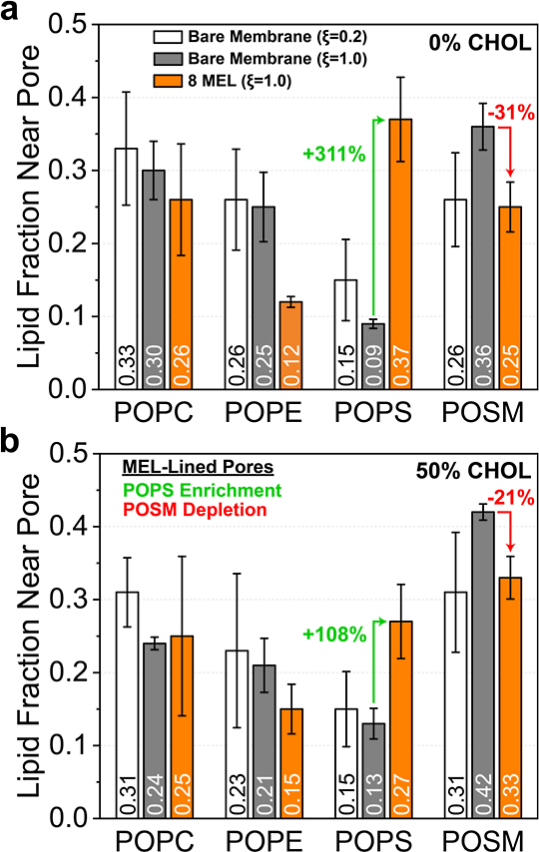
Fractional enrichment of each lipid present in (a) RBC-0%CHOL and (b) RBC-50%CHOL membranes. The lipid fraction near the pore indicates the average number of head phosphate P atoms for each lipid normalized by the average total number of lipid head phosphate P around the pore center in the *xy*-plane of the simulation box. Error bars are standard deviations across 3 independent replicas.

## Conclusions

In this work, we investigate mechanisms of MEL-driven pore nucleation in membranes with compositions that include components of biologically relevant membranes, such as human RBCs. Adapting our previously developed MD simulation methodology,^29^ we modeled four membranes of increasing compositional complexity and two MEL secondary structures to assess the interplay of membrane composition and MEL conformational flexibility on pore nucleation thermodynamics. This was achieved by first initializing MEL-lined structures with MARTINI representations of system species at the minimum membrane defect size required for MEL insertion for each membrane composition and then backmapping finalized equilibrated MARTINI pore structures to the CHARMM36 atomistic force field to resolve free energy profiles with umbrella sampling. We address challenges posed by previous MD studies to model membrane-active species in mammalian-mimetic membranes due to the high computational cost and diffusional limitations imposed by CHOL^59,93^ by utilizing long-time-scale coarse-grained MARTINI simulations at a range of pore sizes created with the nucleation collective variable ξ. To accurately resolve the energetics of MEL-lined membrane configurations (since the MARTINI force field tends to overestimate membrane line tensions and bending moduli during pore formation^29,74^ ), we additionally present newly developed coarse-grained to atomistic backmapping parameters for several lipids utilized in RBC.

We find the inclusion of a high mol % CHOL in RBC membranes (50% CHOL) strongly modulates several membrane properties compared to RBC membranes where CHOL is absent (0% CHOL). With 0% CHOL, MEL rapidly lines small defects with up to five pore-lining MEL peptides, which we attribute to stronger MEL-POPS electrostatic interactions between membrane leaflets that encourage pore nucleation compared to thicker 50% CHOL membranes. Indeed, MEL encourages POPS flip-flop to the upper leaflet and lateral enrichment around the pores more strongly when CHOL is absent. When assessing free energies of atomistic pore configurations, we find that helical MEL reduces the free energy for pore nucleation to a greater degree than kinked MEL, suggesting that the fully α-helical secondary structure for MEL more accurately describes its membrane-bound state prepore nucleation.^25,26,28,80,90,94^ We attribute the low free energy and metastability for MEL-lining pores in RBC membranes without CHOL to the ability of MEL to significantly disrupt sphingomyelin packing and general membrane structural integrity and the increased conformational flexibility of pore-lining MEL when POPS enriches the transmembrane pore. The addition of 50% CHOL substantially increases the free energy of pore nucleation for both POPC and RBC membranes, which is broadly consistent with experimental observations that a high % CHOL inhibits MEL hemolytic activity^41^ and may lead to alternative mechanisms of membrane disruption.^95,96^ These insights will serve as a basis for future MD studies to model mechanisms of biological activity of therapeutically relevant synthetic AMPs^17,97,98^ in compositionally complex lipid membranes.

## Supplementary Material





## Data Availability

Raw simulation data and trajectory analysis scripts have been uploaded to a Dryad repository associated with this manuscript (10.5061/dryad.ttdz08m92) to facilitate reproducibility of the results in this work.

## Notes

Additional information on membrane parameterization, backmapping procedure, umbrella sampling, and simulation trajectory analysis to support the results and figures in the manuscript (PDF)
Map files utilized for backmapping coarse-grained to atomistic membrane components (POPC, POPE, POPS, POSM, and CHOL) (ZIP)
