# Supplementary material for: Elucidating the Impact of Red Blood Cell Membrane Components on Melittin-Induced Pore Formation with Molecular Dynamics Simulations
Source: J Phys Chem B. 2025 Sep 22;129(39):9983–97. doi: 10.1021/acs.jpcb.5c04289 (PMC12591385; doi:10.1021/acs.jpcb.5c04289)
Supplement: Supplementary file 1 [file jp5c04289_si_001.pdf]

Supporting Information for

Elucidating the Impact of Red Blood Cell Membrane Components on Melittin-Induced Pore Formation with Molecular Dynamics Simulations

Joshua D. Richardson[1] and Reid C. Van Lehn[1,2,*]

[1]Department of Chemical and Biological Engineering, University of Wisconsin – Madison, Madison, WI, 53706, USA.

[2]Department of Chemistry, University of Wisconsin – Madison, Madison, WI, 53706, USA.

*address correspondence to: vanlehn@wisc.edu

S1: MARTINI System Preparation and Membrane Asymmetry Considerations

As discussed in the '**Overview of Membranes and Melittin Conformations**' section in the main text, bead types and parameters for all phospholipids (POPC, POPE, POPS, POSM) and CHOL were taken from the MARTINI 2.2 force field.[1] **Figure S1** shows the atomistic to coarse-grained MARTINI mapping strategy for each membrane element as ovals with captions for bead names. Green and red ovals denote the 2 head group beads that constitute each lipid, where the negatively charged PO4 bead (red), which maps the head phosphate group of phospholipids, is shared across all 4 lipids. The green bead visualized in **Figure S1** is lipid-specific: NC3 (positively charged choline group) for POPC and POSM lipids, NH3 (positively charged ethanolamine) for POPE lipids, and CNO (neutral serine) for POPS lipids. Blue beads denote the phospholipid linker and tails, which are identical for POPC, POPE, and POPS lipids. GL1 and GL2 beads capture the glycerol linker region whereas the AM1 and AM2 beads capture the hydroxyl (AM1) and amide (AM2) chemical groups unique to the sphingosine backbone[2] of POSM in this study. For the 8 tail beads in POPC, POPE, and POPS and the 7 tail beads in POSM, the first letter of the bead name captures the underlying saturation of the carbon chain section: 'C' = single bond, 'D' = cis double bond, 'T' = trans double bond.

Cholesterol (CHOL) is mapped to the MARTINI 2.2 force field with 8 beads (purple ovals in **Figure S1**) that capture both the rigid steroid ring (ROH – R5) and flexible hydrocarbon side chain (C1, C2). We adapted parameters provided by Melo et. al for use with the MARTINI force field, which utilize an additional virtual site construction to increase the stability and packing of CHOL in simulations of lipid bilayers.[3]

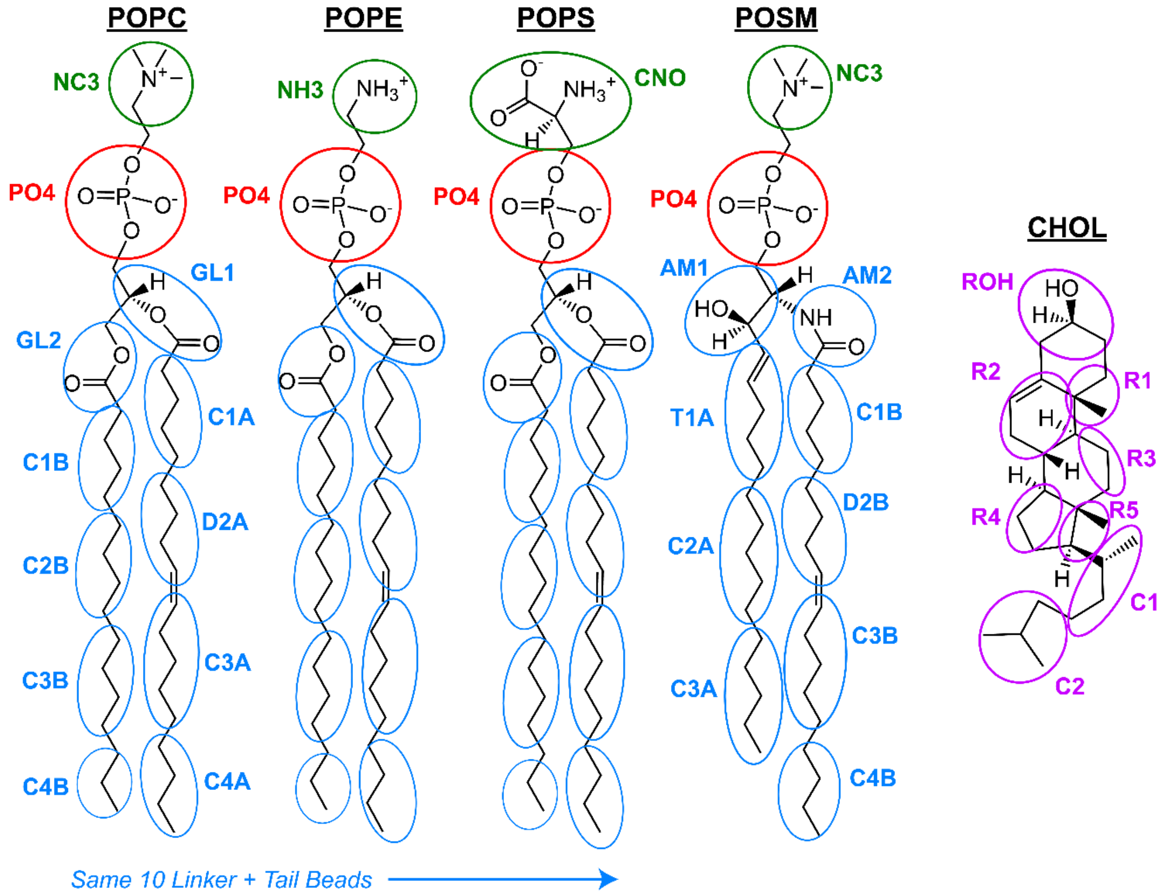

Figure S1: Mapping strategy for all lipids (POPC, POPE, POPS, POSM) and CHOL used in MARTINI membranes in this study. Mapped MARTINI beads are shown as captioned ovals and are colored as follows: Phospholipid tail and linker beads = blue, phospholipid head groups = red (PO4 phosphate bead) or green (lipid-specific NC3, NH3, CNO bead), and CHOL beads = pink.

To set up MARTINI RBC bilayers in our study (RBC-0%CHOL and RBC-50%CHOL – see **Figure 1a**), we first ran 50 ns simulations of symmetric membranes of both the upper and lower leaflet (with equal number of phospholipids and CHOL in each leaflet) to ensure similar leaflet areas and minimize membrane lateral tension in flat bilayers as recommended for building complex asymmetric membranes with the MARTINI force field.[4] Initial leaflet compositions for each membrane are provided with bilayer visualizations in **Figures S2-S3**. These compositions are selected to target human RBC phospholipid leaflet compositions (provided in the '**Overview of Membranes and Melittin Conformations**' section of the main text). Given the slightly larger area per lipid (APL) for the upper leaflet (**Figures S2b, S3b**), we adjusted the number of phospholipids and CHOL accordingly to better match the average APL between leaflets for both RBC membranes (shown in **Table S1** below).

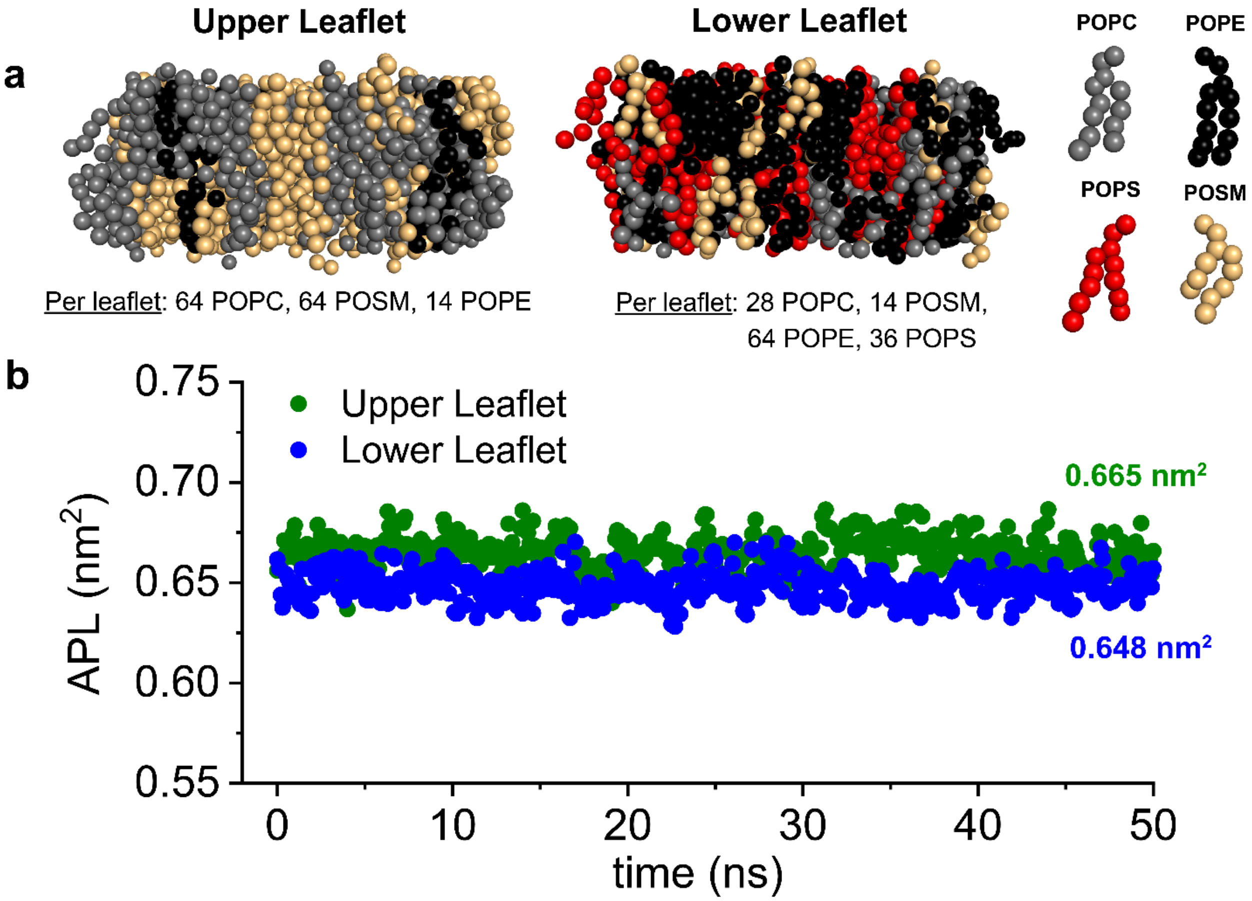

Figure S2: Symmetric MARTINI simulations to match upper and lower leaflet area per lipid (APL) parameters for the RBC-0%CHOL system (**Figure 1a**). (a) Per-leaflet compositions are shown for the upper (left) and lower (right) leaflet with the total number of lipids per leaflet written below each MARTINI membrane representation. Lipids are shown as beads and colored as shown in the key to the right. (b) APL results from 50 ns unbiased simulations for the upper (green) and lower (blue) leaflet symmetric membranes shown in (a). Given an average APL=0.665 nm^2 for the upper leaflet symmetric membrane and APL=0.648 nm^2 for the lower leaflet symmetric membrane, we removed 2 POPC and 2 POSM lipids from the upper leaflet when preparing the asymmetric RBC-0%CHOL membrane to better match the APL between leaflets as shown in **Table S1**.

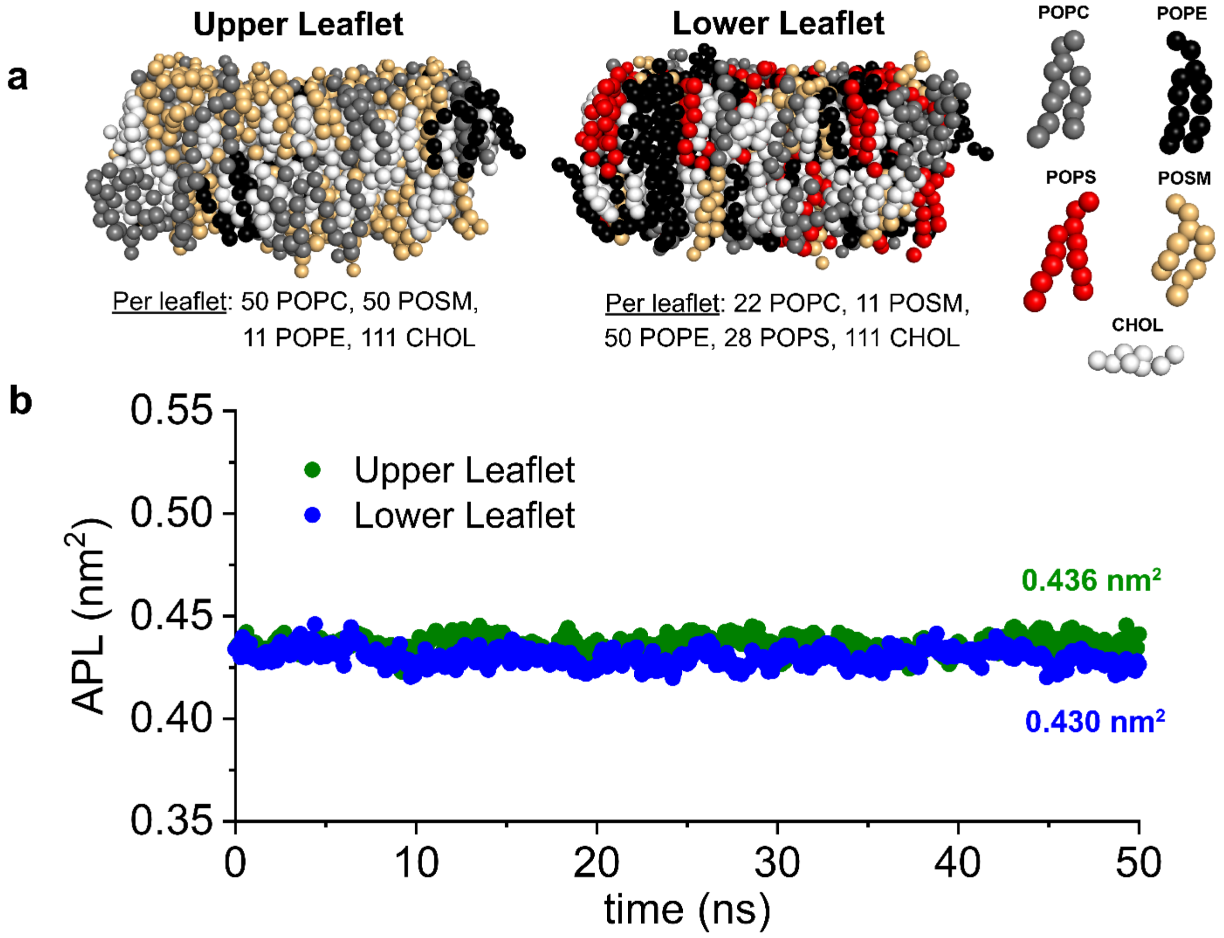

Figure S3: Symmetric MARTINI simulations to match upper and lower leaflet area per lipid (APL) parameters for the RBC-50%CHOL system (**Figure 1a**). (a) Per-leaflet compositions are shown for the upper (left) and lower (right) leaflet with the total number of lipids per leaflet written below each MARTINI membrane representation. Lipids are shown as beads and colored as shown in the key to the right. (b) APL results from 50 ns unbiased simulations for the upper (green) and lower (blue) leaflet symmetric membranes shown in (a). Given an average APL=0.436 nm^2 for the upper leaflet symmetric membrane and APL=0.430 nm^2 for the lower leaflet membrane, we removed 1 POPC, 1 POSM, and 2 CHOL from the upper leaflet when preparing the asymmetric RBC-50%CHOL membrane to better match the APL between leaflets as shown in **Table S1**.

S2: Full Simulation Workflow Example

Figure S4 shows a full example workflow from initial MARTINI simulation setup (**Figure S4a**) to atomistic umbrella sampling simulations with CHARMM36 (**Figure S4h**) for a POPC-0%CHOL system with eight helical MEL. Representative top and side views are shown for each step and referenced throughout the '**Methods**' in the main text.

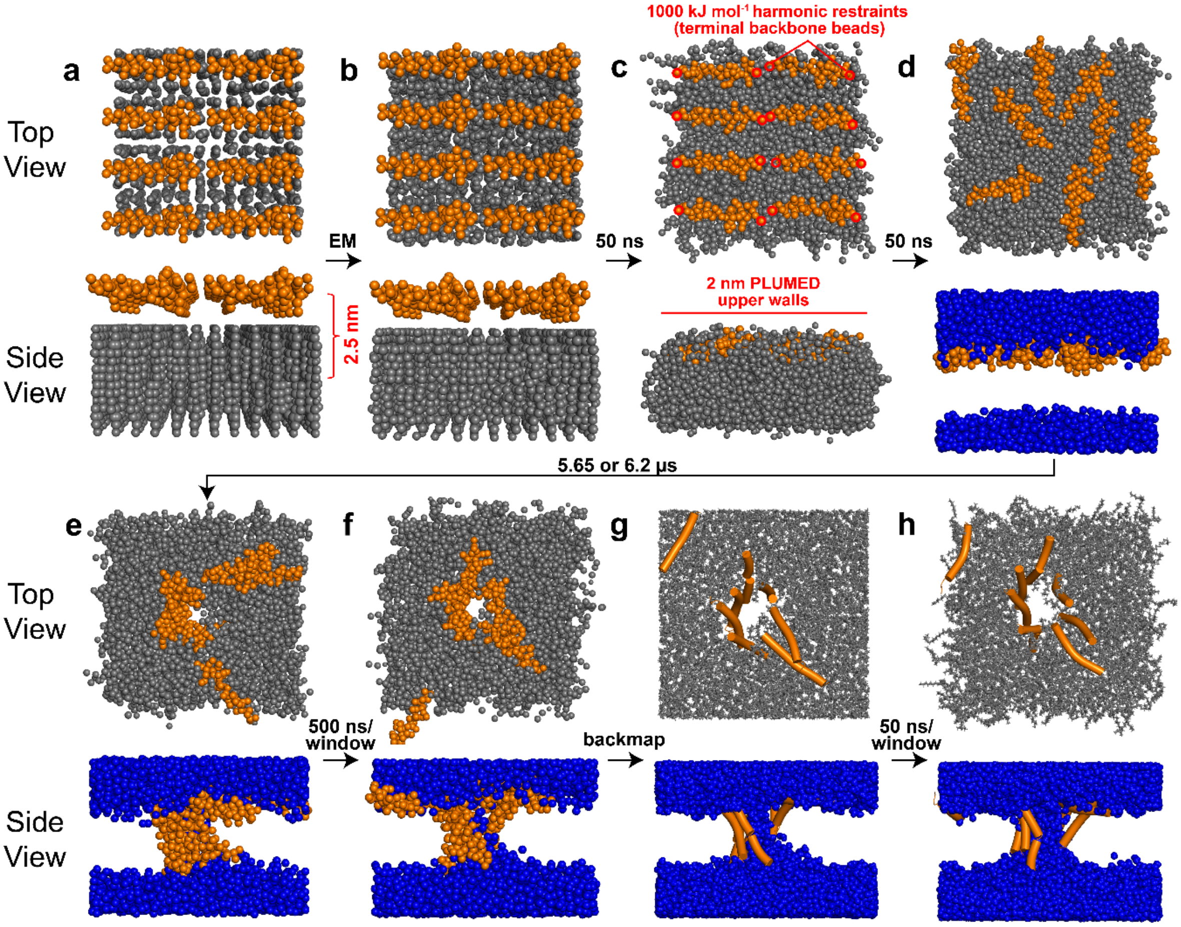

Figure S4: Full simulation workflow for a POPC-0%CHOL membrane system with eight helical MEL. (a) First, a grid of eight MEL (orange beads) is initialized 2.5 nm above the *z*-component of the COM of the membrane (grey beads) and solvated with W beads (blue) and 150 mM NaCl (omitted for clarity) using the *insane* tool. This system is then (b) energy minimized and (c-d) equilibrated to initialize MEL bound to the membrane upper leaflet. This equilibrated membrane (ξ=0.2) is then pulled across either 5.65 (**Figure S7**) or 6.2 μs (**Figure 2a**) of simulation time depending on ξ_{long} to (e) the fully nucleated pore state. 23 umbrella sampling windows are then extracted from this pulling simulation (d→e) and simulated for an additional 500 ns per window (ξ=1.0 window shown in f). These 23 windows are then (g) backmapped to the CHARMM36 force field and (h) simulated for an additional 50 ns per window. Water is not shown in panels (a)-(c) and lipids are not shown in the side view snapshots of panels (d)-(h) for visual clarity.

S3: Long Timescale Pulling and MEL Pore Lining

As discussed in the '**System Setup**' section in the main text, MEL-containing membranes were initialized using our previous methodology for MEL in DMPC bilayers.[5] To ensure that analysis of MEL pore-lining potential during coarse-grained simulations utilizing the nucleation collective variable (ξ) was solely affected by differences in bilayer properties (e.g., thickness, charge) as a result of changes in phospholipid and CHOL content and not diffusional limitations of MEL towards the pore due to mismatches in membrane area, we targeted a membrane area of approximately 100 nm^2 for all MEL-containing systems. **Figure S5** shows the average membrane area across 3 independent 50 ns replicas for each system using the final selected lipid compositions (**Table S1**), which demonstrate good agreement in membrane area around 100 nm^2 (at most a ~5% difference in average area between membranes).

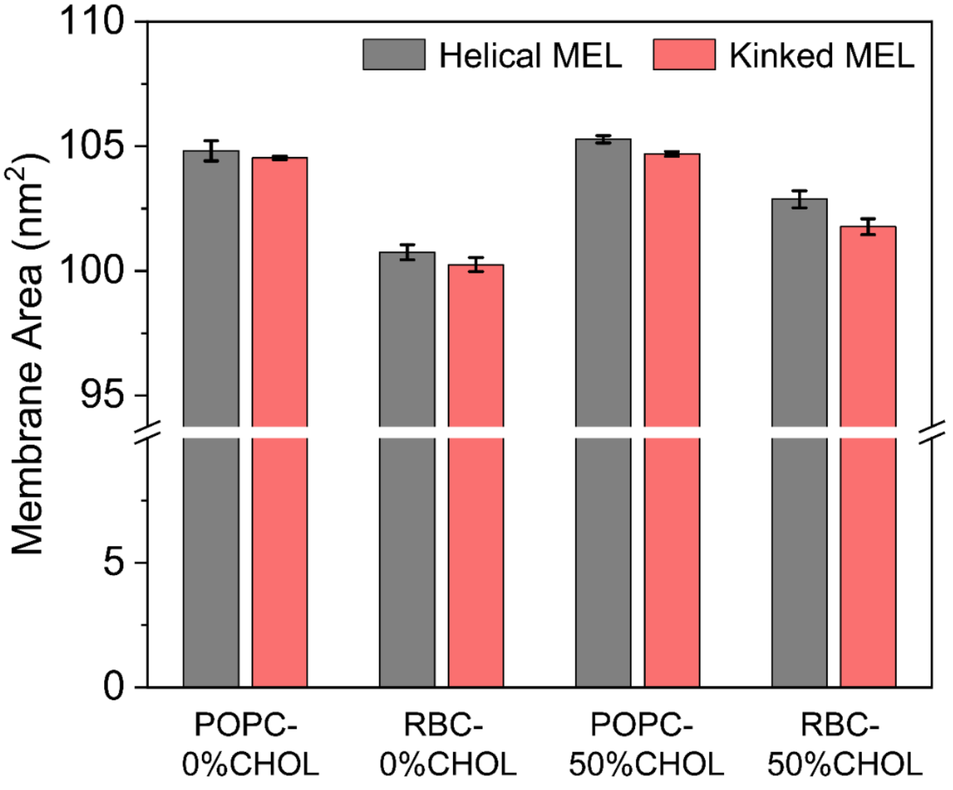

Figure S5: Average membrane area results from 50 ns unbiased MARTINI simulations for membranes (**Figure 1a**) containing both helical (black) and kinked (red) MEL. Error bars indicate standard deviations across 3 independent replicas for each system. These results are based on lipid compositions shown in **Table S1** to target an average membrane area of 100 nm^2 for each system.

Table S1 summarizes the components of all systems modeled with the MARTINI force field. System compositions are provided as molecule counts for the number of lipids (POPC, POPE, POPS, POSM) and CHOL per leaflet (upper leaflet and lower leaflet) as well as the solvent composition breakdown by the number of water W beads and ions to target a physiological salt concentration of 150 mM. The number of W beads was chosen for each system to target a minimum 10:1 ratio of W beads to membrane components (Lipids + CHOL) to minimize interbilayer interactions across periodic boundaries in the *z*-direction.[5] We used the same membrane lipid and CHOL compositions for membranes without MEL and membranes with either MEL. For membranes with MEL, values in parenthesis denote the updated number of W, NA+, and CL- beads to neutralize the +48 charge imposed by eight +6-charged MEL peptides.

Table S1: Membrane and solution compositions for all MARTINI simulations. Each membrane type (**Figure 1a**) is listed in the top row. The number of lipid molecules (POPC, POPE, POPS, POSM) and CHOL are shown per leaflet (upper leaflet and lower leaflet). Solution components include water beads (W) and sodium (NA+) and chloride (CL-) ions. Values in parenthesis denote adjustments to the solvent for systems containing eight +6-charged helical or kinked MEL.

		**POPC-0%CHOL**	**RBC-0%CHOL**	**POPC-50%CHOL**	**RBC-50%CHOL**
**Upper Leaflet**	**POPC**	144	62	112	49
	**POPE**	---	14	---	11
	**POPS**	---	---	---	---
	**POSM**	---	62	---	49
	**CHOL**	---	---	112	109
**Lower Leaflet**	**POPC**	144	28	112	22
	**POPE**	---	64	---	50
	**POPS**	---	36	---	28
	**POSM**	---	14	---	11
	**CHOL**	---	---	112	111
	**W**	2893 (2973)	2904 (2976)	4518 (4495)	4521 (4443)
	**NA+**	32 (9)	50 (27)	50 (25)	64 (39)
	**CL-**	32 (57)	14 (39)	50 (73)	36 (59)

To create transmembrane pores in MARTINI membranes, the nucleation collective variable (ξ) initially proposed by Hub et. al[6] and adapted in our previous study to coarse-grained lipid membranes[5] was implemented. Equation S1 defines ξ as:

$$\xi = N_s^{-1} \sum_{s=0}^{N_s-1} \delta_s(N_s^{(p)}) \tag{S1}$$

where $N_s^{(p)}$ is the number of lipid phosphate PO4 and water W beads in a slice s of a transmembrane cylinder of radius R = 0.8 nm and N_s slices of thickness d_s = 0.2 nm each (**Figure S6a**). A switching function δ_s is applied to $N_s^{(p)}$ to smoothly increase $\delta_s(N_s^{(p)})$ from 0 to 1 for each slice.[6] In past studies, ξ ≈ 0.2 for flat membranes with no defect (increasing to ξ = 1.0 for full nucleation),[6] so we determined the N_s value for each membrane that most closely matches ξ = 0.2 (**Figures S6b-e**) during 50 ns unbiased simulations. For all MARTINI simulations that utilize ξ, we set N_s = 19 slices for membranes without CHOL (both POPC and RBC, **Figures S6b-c**) and N_s = 22 slices for membranes with 50% CHOL (both POPC and RBC, **Figures S6d-e**).

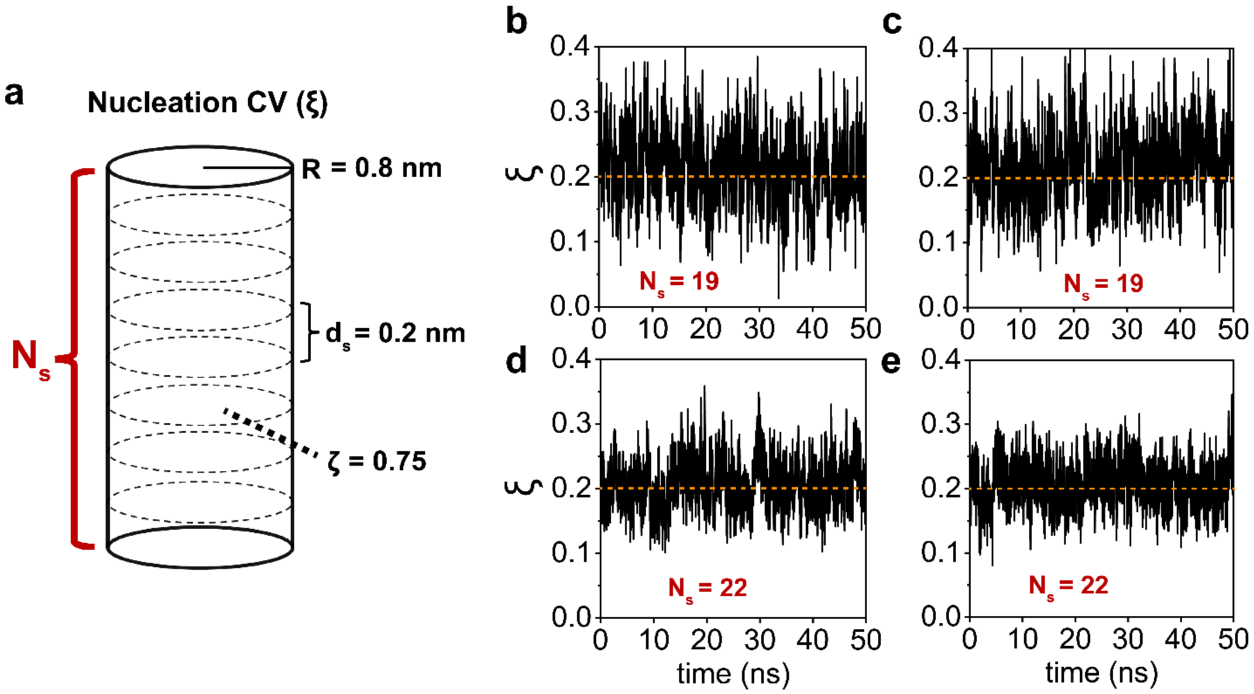

Figure S6: (a) Schematic demonstrating the parameters used to calculate the nucleation collective variable (ξ) in coarse-grained MARTINI bilayers. ξ biases polar lipid PO4 and water W beads towards a transmembrane cylinder defect of radius R with N_s slices of thickness d_s and occupancy factor ζ (value for addition of first polar bead to a slice). For all membranes, we set R = 0.8 nm, d_s = 0.2 nm, and ζ = 0.75 for consistency with our previous study in DMPC bilayers. The number of transmembrane cylinder slices (N_s) was chosen independently for each membrane with a 50 ns simulation to target a value of ξ = 0.2 (orange dashed line) for flat unbiased membranes. To confirm that the choice of N_s is reasonable, fluctuations of ξ as a function of simulation time from unbiased simulations are shown for (b) N_s = 19 for POPC-0%CHOL, (c) N_s = 19 for RBC-0%CHOL, (d) N_s = 22 for POPC-50%CHOL, and (e) N_s = 22 for RBC-50%CHOL.

Figure S7 is provided to support **Figure 2a** in the main text. During the long-timescale equilibration simulations, each replica is independently equilibrated and ξ is increased over 50 ns from the flat membrane state (ξ=0.2) to the beginning of the transition state for pore nucleation (ξ_{long}=0.7), which is then simulated for 5 μs restrained at ξ_{long}=0.7 to observe if there is any MEL pore lining by 5.05 μs. This methodology is iteratively restarted, increasing ξ_{long} by 0.025 each time until at least 1 MEL lines the pore by 5.05 μs (red 't_{cutoff}' line in **Figures 2a, S7**). **Figure 2a** demonstrates the full 6.2 μs simulation starting at ξ_{long} = 0.7 and increasing to ξ_{long} = 0.775 if needed, whereas **Figure S7** demonstrates continued ξ_{long} iterations if required starting at ξ_{long} = 0.8 and simulated for 5.65 μs each (1 fewer 50 ns pulling and 500 ns restrained equilibration step at ξ = 0.8 compared to simulations with $0.7 \leq \xi_{long} \leq 0.775$ as shown in **Figure 2a**).

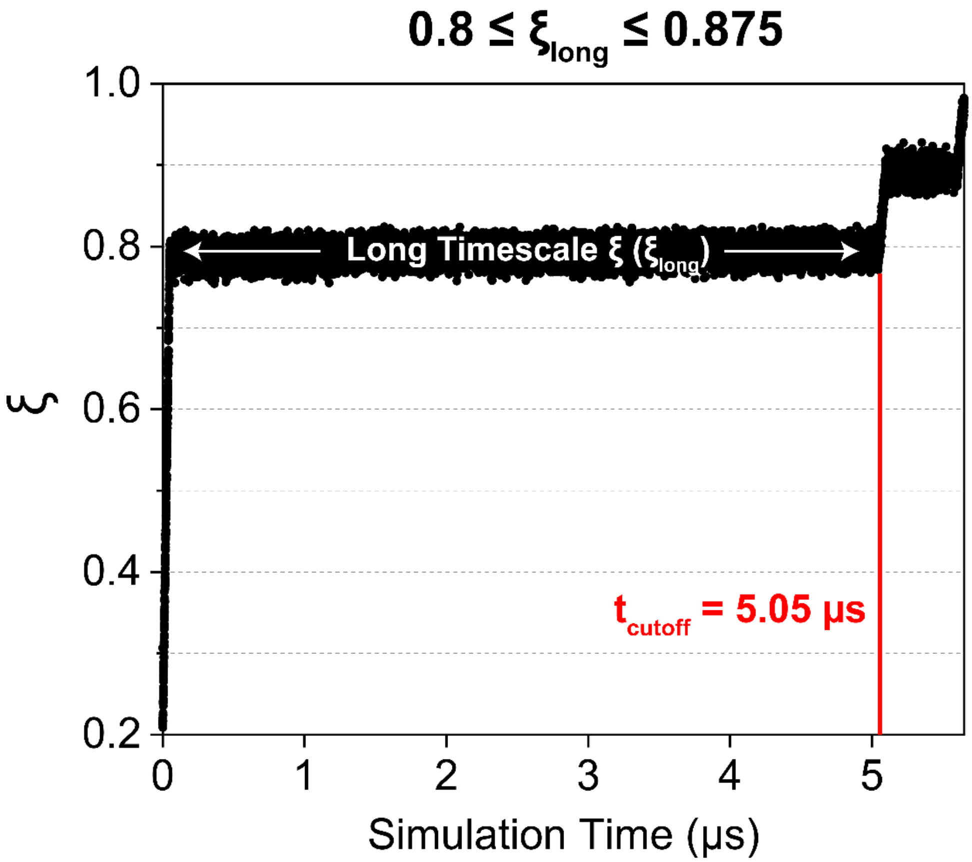

Figure S7: Equilibration procedure for $0.8 \leq \xi_{long} \leq 0.875$ to demonstrate the steered MD procedure if no MEL line the defect for $0.7 \leq \xi_{long} \leq 0.775$. The equilibrated coarse-grained system is first pulled from ξ = 0.2 to ξ_{long} (initially set as 0.8) for 50 ns and then restrained at ξ_{long} for 5 μs. If no MEL lines by t_{cutoff} = 5.05 μs (shown in red), then the system is reset iteratively increasing ξ_{long} by 0.025 each time until MEL pore-lining is observed by the t_{cutoff}. The system is then taken through alternating 50 ns steered MD and 500 ns restrained equilibration steps at ξ values in multiples of 0.1 until the full pore state (ξ = 1.0) is reached at 5.65 μs.

S4: Coarse-Grained to Atomistic Backmapping

This section details the methodology and backmapping parameterization for converting MARTINI coarse-grained configurations to atomistic configurations. We built upon work by Wassenaar et. al, which established the backmapping methodology[7], and our previous work which adapted this backmapping workflow to AMPs and DMPC lipids.[5] All 10 steps required to backmap a coarse-grained MARTINI membrane system to an atomistic CHARMM36 system are provided in **Table S2**. **Figure S8** demonstrates the backmapping procedure (Step 1 in **Table S2**) and energy minimized structures (after Step 3) of all 4 lipids utilized in this study. After estimating atomic positions through projection estimation within MARTINI beads (transparent spheres in **Figure S8**), all additional local geometry reconstruction considerations are indicated by blue text. Some examples of these reconstructions include ensuring the R stereoisometry of a hydrogen in the linker region of each lipid, head group chirality of some lipids (e.g., choline and serine group), and the presence of cis or trans double bonds in lipids tails.

After backmapping, systems were energy minimized in 2 steps (Steps 2-3 in **Table S2**) for a maximum of 1000 steps each. First, nonbonded MEL-MEL and Membrane-Membrane interactions were turned off to prevent high energies due to atomic overlap (Step 2), and then all nonbonded interactions were turned back on (Step 3). For both EM steps, select dihedral restraints on lipid chemical groups (e.g., cis double bond in oleoyl tail) were applied, and the steepest descent algorithm was implemented with a maximum step size of 0.1 nm and tolerance of 100 kJ mol^{-1} nm^{-1}. Lastly, each system was subject to 4 position restrained *NVT* simulations (Steps 4-7 in **Table S2**), 2 position restrained *NPT* simulations (Steps 8-9), and 1 *NPT* simulation with no restraints (Step 10). For all MD simulation steps (Steps 4-10), the temperature was set to 310 K to match the average physiological temperature and was controlled with a velocity-rescale thermostat with time constant of 1 ps. The Verlet cutoff scheme with a buffer tolerance of 0.005 kJ mol^{-1} ps^{-1} was implemented for neighbor searching. Lennard-Jones interactions were smoothly switched to zero from 1.0 nm to 1.2 nm and Coulombic interactions were cut off at 1.2 nm. For *NPT* simulations, the pressure was controlled at 1 bar with the Berendsen barostat with semi-isotropic pressure coupling with 4.5×10^{-5} bar^{-1} compressibility and a 5 ps time constant.

Table S2: Backmapping and equilibration steps for converting MARTINI systems to CHARMM36 configurations. The ‘Δt’ column refers to the simulation time step in ps where applicable. The ‘Steps’ column defines the maximum (for Steps 2-3) or total (for Steps 4-10) number of simulation steps. The ‘Restraints’ column denotes if dihedral or position restraints are enabled. Additional considerations for each step are presented in the ‘Notes’ column.

Step	Name	Δt (ps)	Steps	Restraints	Notes
1	Backmap	-----	-----	-----	Backmap with .map files
2	EM1	-----	1000	Dihedral	MEL-MEL & Memb.-Memb. nonbonded interactions turned off
3	EM2	-----			
4	NVT1	0.0002	500	Position	NVT Equilibration
5	NVT2	0.0005			
6	NVT3	0.001			
7	NVT4	0.002			
8	NPT1	0.001	500	Position	NPT Equilibration
9	NPT2	0.002			
10	NPT3	0.002		-----	

To validate the backmapping accuracy of each lipid (POPC, POPE, POPS, POSM), we evaluated 3 different membrane metrics in 64-lipid single-lipid membranes: APL (**Figure S9a**), membrane thickness (**Figure S9b**), and deuterium order parameters (S_{CD}, **Figure S10**). For atomistic systems, we initialized single lipid membranes with the CHARMM-GUI server[8] and used the same simulation parameters compatible with the CHARMM36 force field as atomistic umbrella sampling for 3 independent 50 ns simulations. For backmapped atomistic systems, we first initialized equivalent MARTINI membrane compositions using the *insane* tool[9] and used the same simulation parameters compatible with the MARTINI force field as coarse-grained simulations for 3 independent 50 ns simulations. Each of these systems were then fully backmapped (**Table S2**) and simulated for an additional 50 ns using the same parameters as the atomistic systems for direct comparison of membrane properties.

As shown in **Figure S9**, the same methodology was used to evaluate APL and membrane thickness as for POPC and RBC membranes in the main text. These metrics demonstrate excellent agreement within error between atomistic (grey) and backmapped atomistic (red) systems. The deuterium order parameter (S_{CD}), which captures the lipid tail order relative to the membrane normal[10], is also in good agreement between atomistic (black) and backmapped atomistic (red) systems (**Figure S10**).

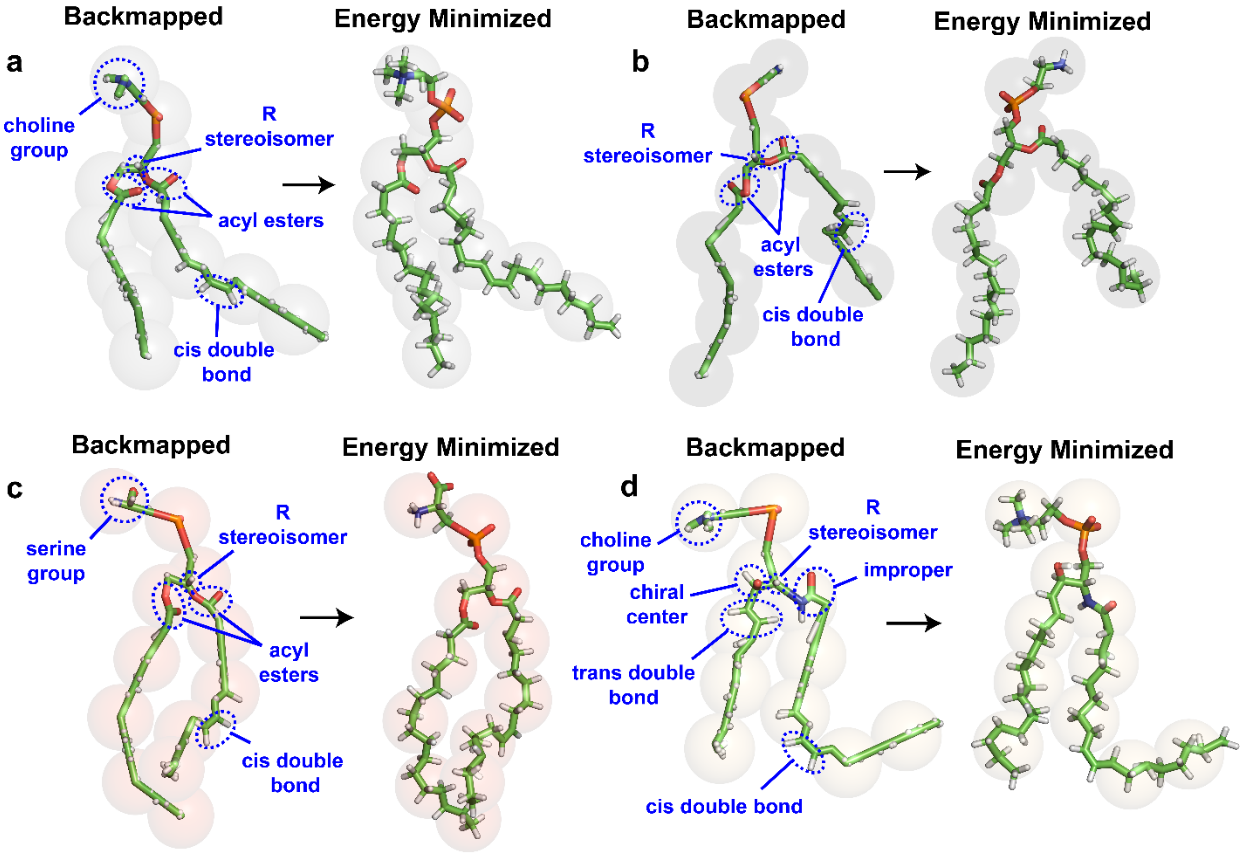

Figure S8: MARTINI to CHARMM36 backmapping (left, Step 1 in **Table S2**) and energy minimization (right, Steps 2-3 in **Table S2**) for the 4 lipids considered in this study: (a) POPC, (b) POPE, (c) POPS, and (d) POSM. Coarse-grained lipid beads modelled with the MARTINI force field are shown as transprent spheres and backmapped atomistic positions modelled with the CHARMM36 force field are shown as opaque sticks. Blue captions denote all additional geometric corrections that are accounted for in backmapping .map files.

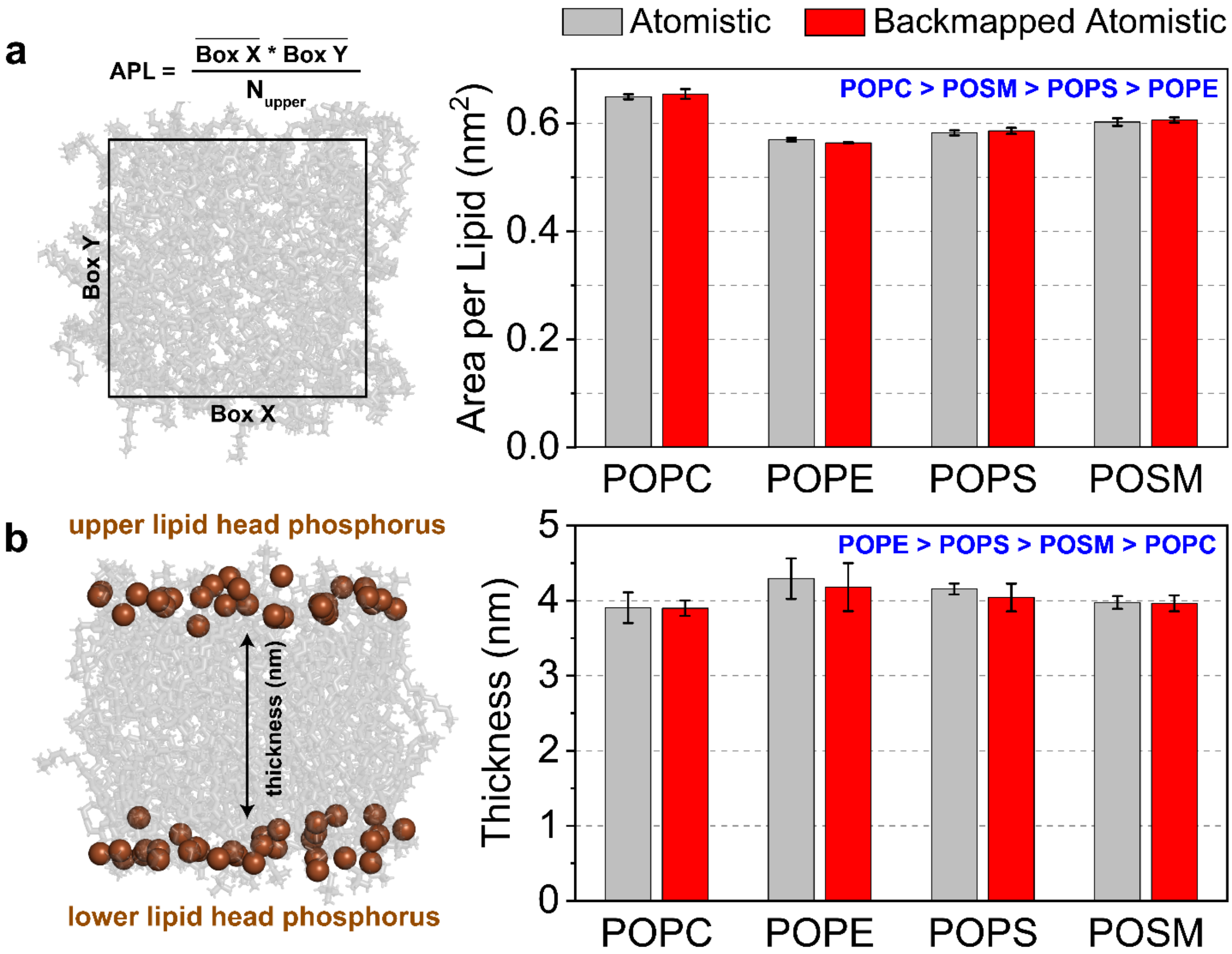

Figure S9: Validating the (a) area per lipid (APL) and (b) membrane thickness for POPC, POPE, POPS, and POSM membranes containing 64 lipids (32 per leaflet). Atomistic simulation data is light grey and backmapped atomistic data is red. Simulation shapshots show all lipids as grey sticks and head group phosphorus atoms as brown spheres and illustrate the calculation of the APL and thickness. Error bars indicate standard deviations across 3 independent replicas.

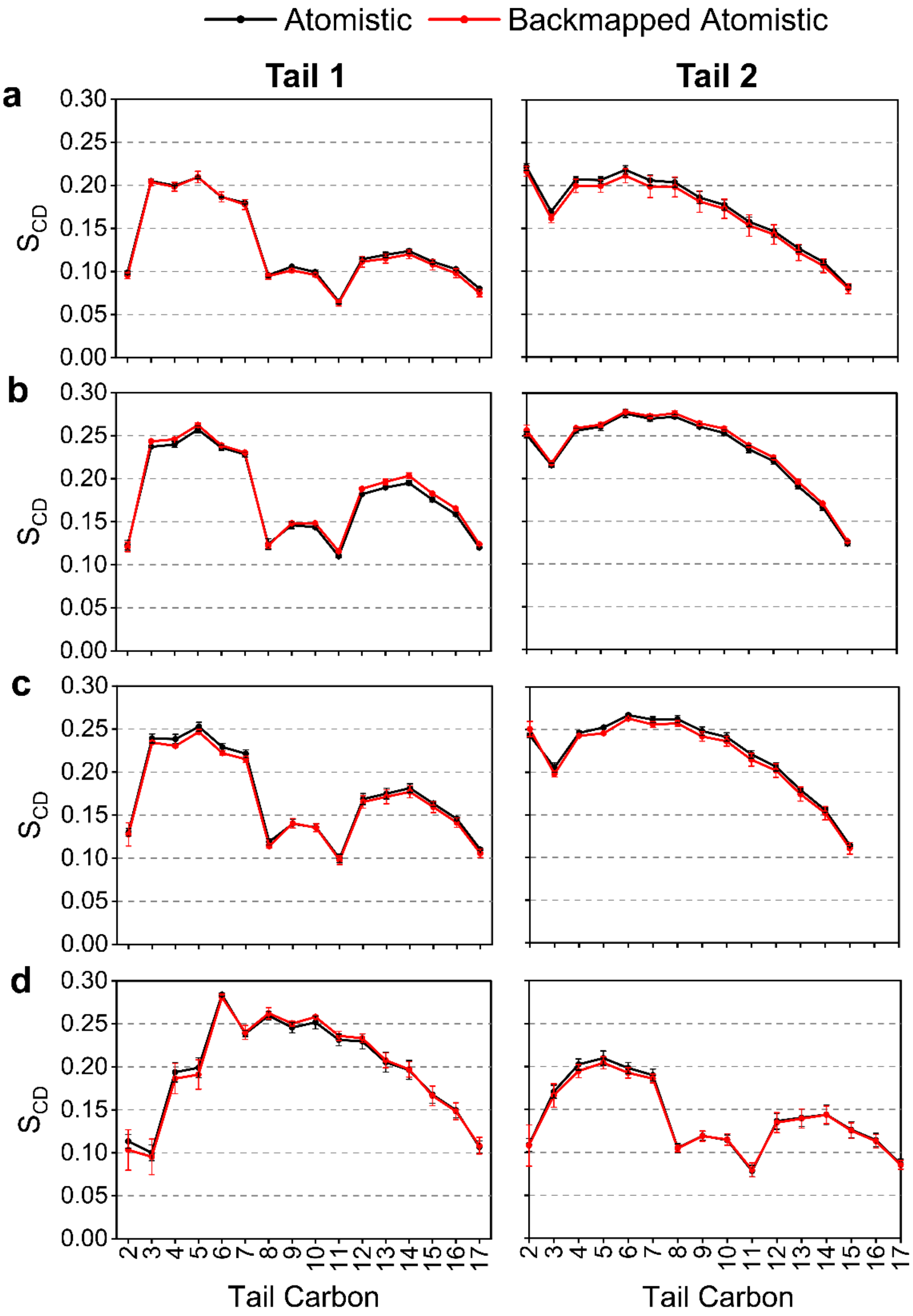

Figure S10: Atomistic (black) vs backmapped atomistic (red) S_{CD} curves for the following 64-lipid membranes: (a) POPC, (b) POPE, (c) POPS, and (d) POSM. S_{CD} values are plotted as the average across 3 replicas for each tail carbon with standard deviations visualized as error bars.

Additionally, the MARTINI to CHARMM36 backmapping parameters for CHOL were slightly adjusted based on the original mapping file[7] to better recreate the 8 chiral centers (circled in blue in **Figure S11**) present in the atomistic structure of CHOL (**Figure S1**).

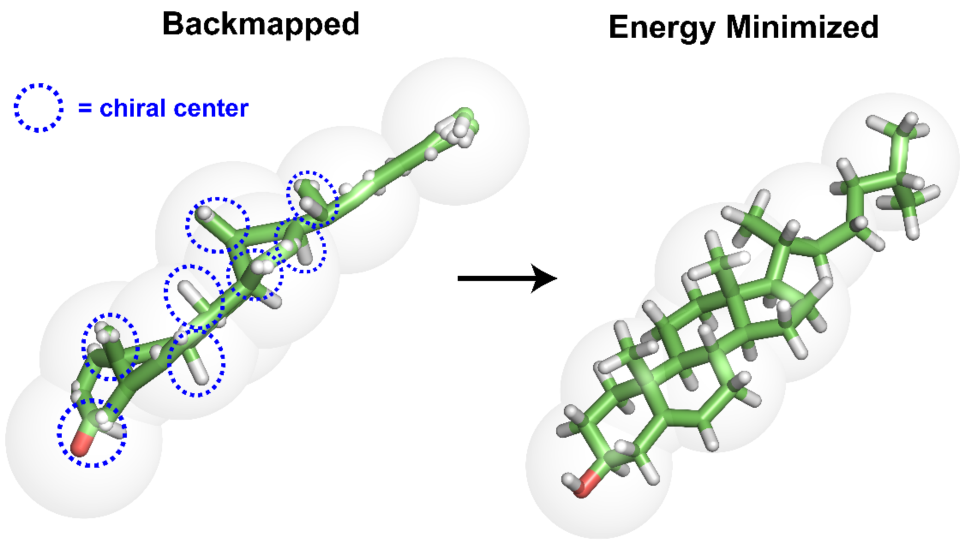

Figure S11: CHOL MARTINI to CHARMM36 backmapping (left, Step 1 in **Table S2**) and energy minimization (right, Steps 2-3 in **Table S2**). CHOL coarse-grained beads modelled with the MARTINI force field are shown as transparent spheres and backmapped atomistic positions modelled with the CHARMM36 force field are shown as opaque sticks. Dashed blue circles denote the eight chiral geometric corrections accounted for in the CHOL backmapping .map file.

To implement the nucleation collective variable (ξ) for backmapped atomistic systems, we used the same R = 0.8 nm and ζ = 0.75 parameters for the transmembrane cylinder as the coarse-grained ξ definition (**Figure S6**). We decreased the slice thickness from d_s = 0.2 nm to d_s = 0.1 nm (**Figure S12**) to reflect the 1:4 mapping of MARTINI beads to water molecules[11] for consistency with our previous study in DMPC membranes[5] and the original implementation of ξ.[6] Additionally, ξ biases water O atoms and lipid head P atoms towards the transmembrane cylinder defect compared to lipid PO4 beads and water W beads for MARTINI systems. To determine the number of slices N_s (captioned in red in **Figure S12a**) of d_s = 0.1 nm thickness in the cylinder defect, we ran a 100 ns simulation for each of the 4 bare atomistic membranes (**Figure S12b-e**, same lipid and CHOL composition as MARTINI systems shown in **Table S1**) and calculated ξ at a range of N_s, targeting the N_s that best matched ξ = 0.2 for each membrane. These were N_s = 35 slices for POPC-0%CHOL, N_s = 38 slices for RBC-0%CHOL, N_s = 44 slices for POPC-50%CHOL, and N_s = 45 slices for RBC-50%CHOL. These parameters were utilized for the calculation of ξ in all atomistic umbrella sampling simulations.

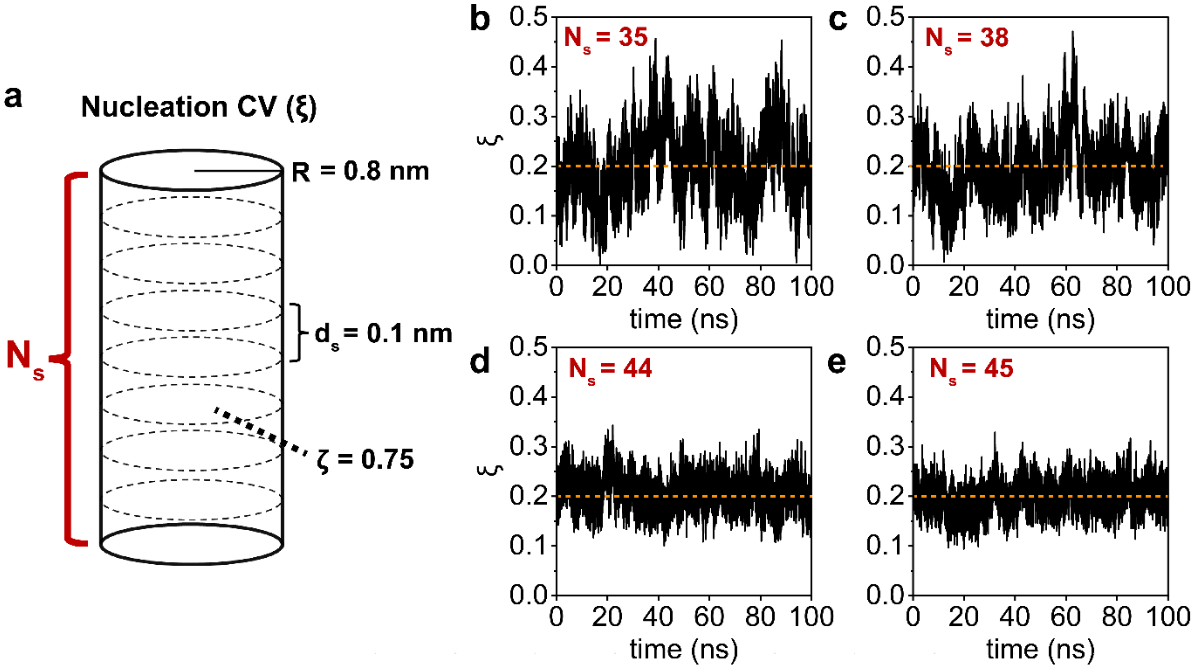

f

$N_s \rightarrow$	35	36	37	38	39	40	41	42	43	44	45
POPC-0%CHOL	0.201	0.221	0.241	0.260	0.279	0.296	---	---	---	---	---
RBC-0%CHOL	0.136	0.156	0.175	0.195	0.214	0.233	---	---	---	---	---
POPC-50%CHOL	---	---	---	---	---	0.131	0.151	0.171	0.190	0.208	0.226
RBC-50%CHOL	---	---	---	---	---	0.103	0.123	0.143	0.162	0.181	0.199

Figure S12: (a) Schematic demonstrating the parameters used to calculate the nucleation collective variable (ξ) in atomistic CHARMM36 bilayers. ξ biases polar lipid head P atoms and water O atoms towards a transmembrane cylinder defect of radius R with N_s slices of thickness d_s and occupancy factor ζ (value for addition of first polar atom to a slice). For all membranes, we set R = 0.8 nm, d_s = 0.1 nm, ζ = 0.75 – for consistency with our previous study in DMPC bilayers. The number of transmembrane cylinder slices (N_s) was chosen independently for each membrane with a 100 ns simulation to target a value of $\xi = 0.2$ (orange dashed line) for flat unbiased membranes. To confirm that the choice of N_s is reasonable, fluctuations of ξ as a function of simulation time from unbiased simulations are shown for (b) $N_s = 35$ for POPC-0%CHOL, (c) $N_s = 38$ for RBC-0%CHOL, (d) $N_s = 44$ for POPC-50%CHOL, and (e) $N_s = 45$ for RBC-50%CHOL. (f) ξ values calculated at a range of N_s ($35 \leq N_s \leq 45$) demonstrating that N_s was chosen for each of the 4 membranes (b-e) to match $\xi = 0.2$ most closely (ξ at selected N_s for each membrane is colored orange).

S5: Lipid Flip Flop and Lateral Enrichment during Long-Timescale Pore Equilibration

This section provides results for all MEL pore-lining (**Figure 2b**) and POPS behavior (**Figure 3**) observed during the long-timescale MARTINI equilibration simulations.

Table S3 details the selected ξ_{long} for each independent replica (R1, R2, R3) as a highlighted 'X'. The average ξ_{long} value and standard deviation across three replicas for each system is shown as black squares with error bars in **Figure 2b**. For instance, the POPC-0%CHOL with helical MEL has ξ_{long} = 0.75, 0.7, 0.75 for the three replicas and is therefore plotted as ξ_{long} = 0.733 with standard deviation 0.029 (solid black square in POPC-0%CHOL section of **Figure 2b**). The vertical orange line in **Table S3** denotes the division between 6.2 μs (**Figure 2a**) versus 5.65 μs (**Figure S7**) long timescale MEL-lining equilibration simulations depending on the selected ξ_{long} during each iteration.

For each selected ξ_{long}, the evolution of the estimated number of pore-lining MEL during each simulation is shown in **Figure S13**. We calculated the number of MEL lining the pore using the same methodology as our previous study[5]:

1) The average density profile (in kg m^{-3}) of MEL is calculated in 50 ns simulation intervals using the *gmx density* tool for a simulation box divided into 50 slices in the *z*-direction.
2) The density profile is integrated from z = -0.5 nm to z = 0.5 nm (where z = 0 nm is the center of the membrane) to resolve the pore peptide density in kg m^{-2}.
3) The total pore peptide density is normalized by the pore peptide density attributed to the lining of 1 pore-lining MEL on a per-system basis based on visual observation of simulation trajectories to calculate the number of pore-lining MEL.

All systems have at least 1 pore-lining MEL by t_{cutoff} = 5.05 μs and the average number of pore-lining MEL calculated across 3 replicas at t_{cutoff} is plotted as a red triangle for each system in **Figure 2b**.

Table S3: Selected ξ_{long} value (highlighted cell with 'X') for each MEL-containing system and replica according to the methodology presented in **Figure 2a** for $0.7 \leq \xi_{long} \leq 0.775$ and **Figure S7** for $0.8 \leq \xi_{long} \leq 0.875$. R1, R2, and R3 correspond to the three replicates of each system. The average ξ_{long} and standard deviation across each set of 3 independent replicas is plotted in black in **Figure 2b**.

Increasing ξ_{long} →

			0.7	0.725	0.75	0.775	0.8	0.825	0.85
Helical MEL	POPC-0%CHOL	R1			X				
		R2	X						
		R3			X				
	RBC-0%CHOL	R1	X						
		R2	X						
		R3	X						
	POPC-50%CHOL	R1		X					
		R2			X				
		R3					X		
	RBC-50%CHOL	R1				X			
		R2				X			
		R3			X				
Kinked MEL	POPC-0%CHOL	R1			X				
		R2	X						
		R3			X				
	RBC-0%CHOL	R1	X						
		R2		X					
		R3	X						
	POPC-50%CHOL	R1					X		
		R2							X
		R3			X				
	RBC-50%CHOL	R1					X		
		R2		X					
		R3						X	

← 6.2 µs (Fig. 2a) → ← 5.65 µs (Fig. S7) →

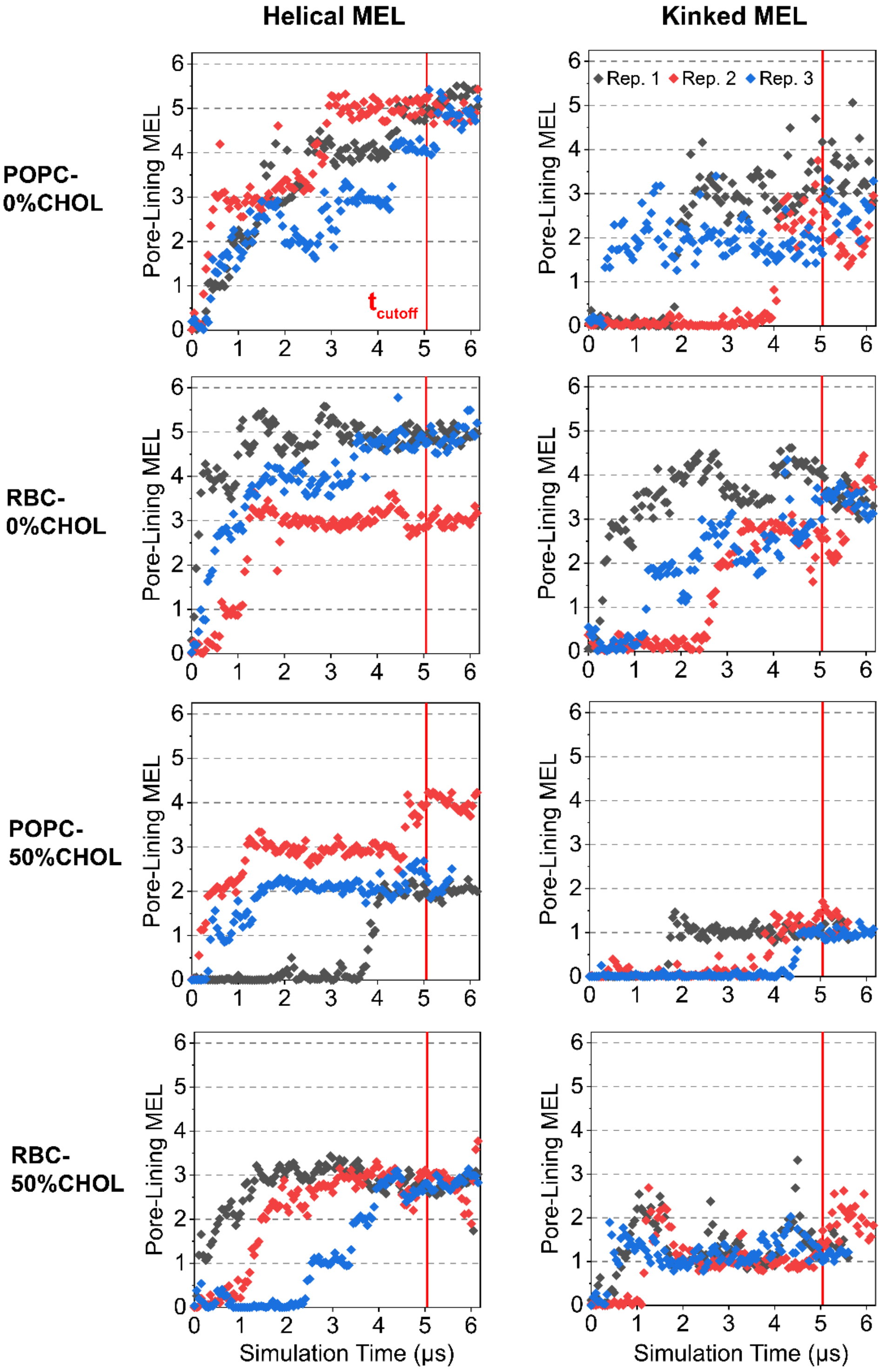

Figure S13: Number of pore-lining MEL vs. simulation time for each selected ξ_{long} in **Table S3**. Each point is calculated as the average density of MEL in the middle 1 nm of the pore in the *z*-direction across a 50 ns block of simulation time normalized by the system-dependent density of 1 pore-lining MEL (POPC-0%CHOL = 11.5 kg m^{-2}; RBC-0%CHOL = 13 kg m^{-2}; POPC-50%CHOL = 14 kg m^{-2}; RBC-50%CHOL = 15 kg m^{-2}). All systems have at least 1 pore-lining MEL by the cutoff time (t_{cutoff} = 5.05 μs, vertical red line).

Figure S14 demonstrates that initially MEL initially in U-lining pore conformations can transition to transmembrane pore configurations over μs timescales during the long-timescale equilibration MARTINI simulations. We compare two metrics from Replica 1 of the RBC-0%CHOL system with kinked MEL for one peptide: Peptide tilt angle and P14 depth. The tilt angle refers to the angle of the vector connecting the N- and C-terminus backbone beads to the *xy*-plane of the membrane and ranges from 0° to 90°. A value of 90° denotes MEL in the fully transmembrane pore-lining configuration. The P14 depth refers to the distance of the Proline-14 sidechain bead to the membrane midplane (defined as the average *z*-coordinate of lipid tail end C4A and C4B beads), which is consistent with a previous methodology for evaluating the penetration depth of P14 for U-shaped MEL in lipid membranes.[12] During 6.2 μs of simulation (following the methodology in **Figure 2a** of the main text), 1 MEL transitions from membrane-bound (0 μs) to U-lining with helical kink (0.4 μs as shown in **Figure S14b**), to a transmembrane conformation (after ~3 μs).

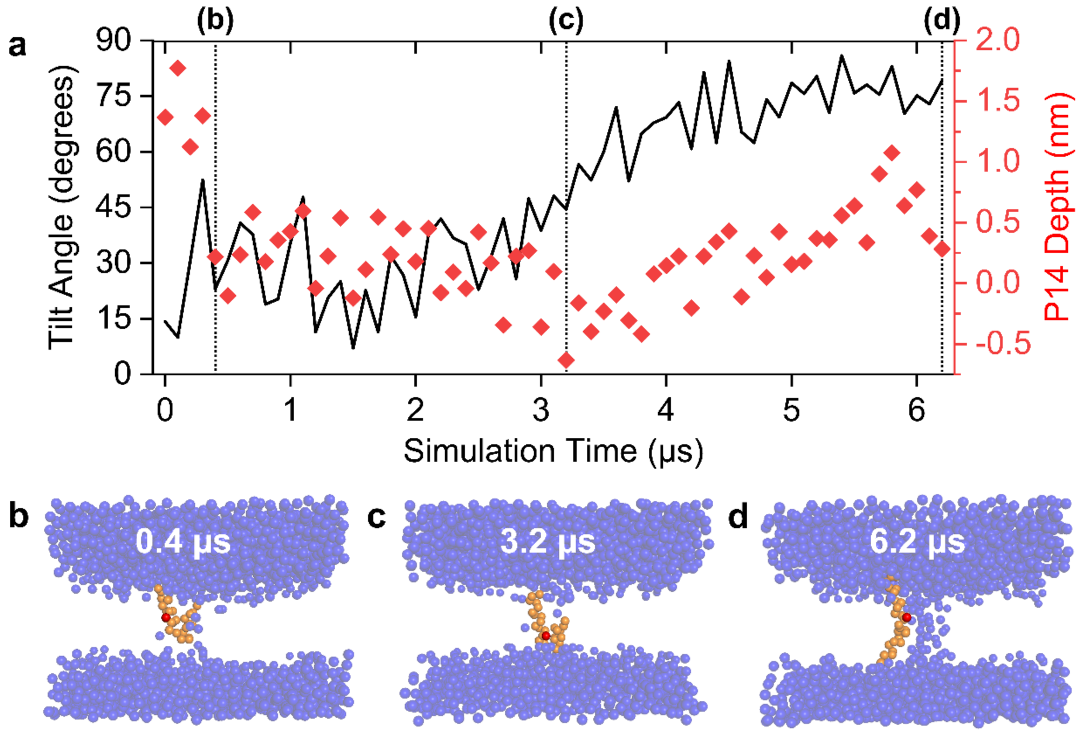

Figure S14: Evolution of 1 'kinked MEL' peptide in the Replica 1 long-timescale MARTINI equilibration simulation for the RBC-0%CHOL membrane system. (a) Comparison of tilt angle (black line) vs. P14 depth (red diamonds) over the simulation trajectory. (b-d) Corresponding configurations are provided to demonstrate the evolution of an initially membrane-bound kinked MEL to a (b) U-shaped and then a (d) fully transmembrane configuration by the end of the trajectory, visualizing (c) the transition between the two states that starts around 3.2 μs.

Below, we provide replicate POPS flip-flop (**Figure S15**) and enrichment (**Figure S16**) results for long timescale MARTINI simulations (**Figures 2a, S7**) in RBC membranes to demonstrate that POPS trends are similar across replicas.

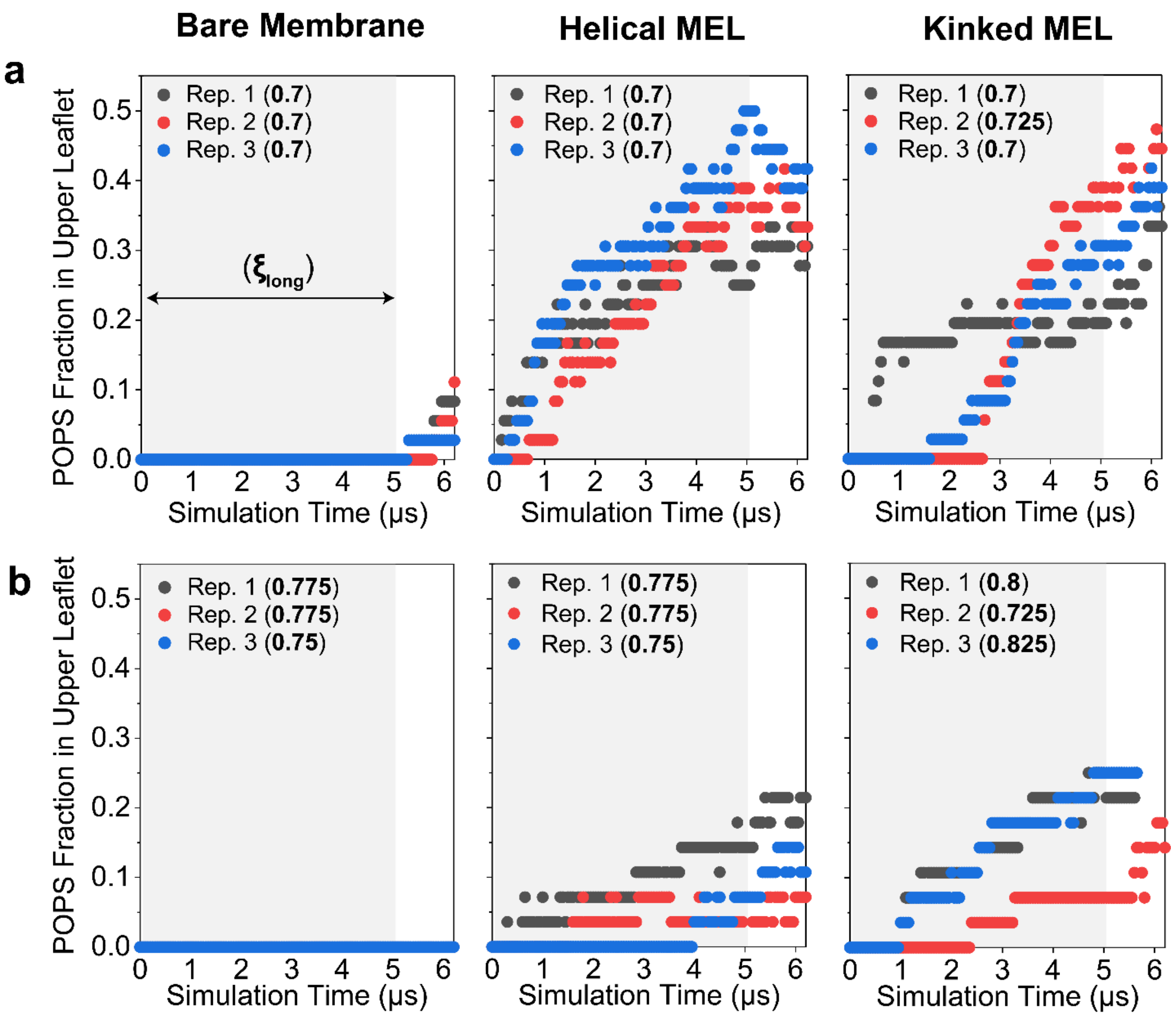

Figure S15: POPS fraction in the upper leaflet calculated for all replicas in support of **Figure 3a** for systems containing (a) 0% CHOL and (b) 50% CHOL. Helical MEL and kinked MEL results (rightmost 2 columns) are plotted for the selected ξ_{long} for each replica (**Table S3**), with ξ_{long} values included in parentheses in the legend. Bare membrane results use the same ξ_{long} as the helical MEL systems. The light grey region corresponds to the 5 μs of simulation restrained at the ξ_{long} selected for each system from t = 50 ns to t = 5.05 μs.

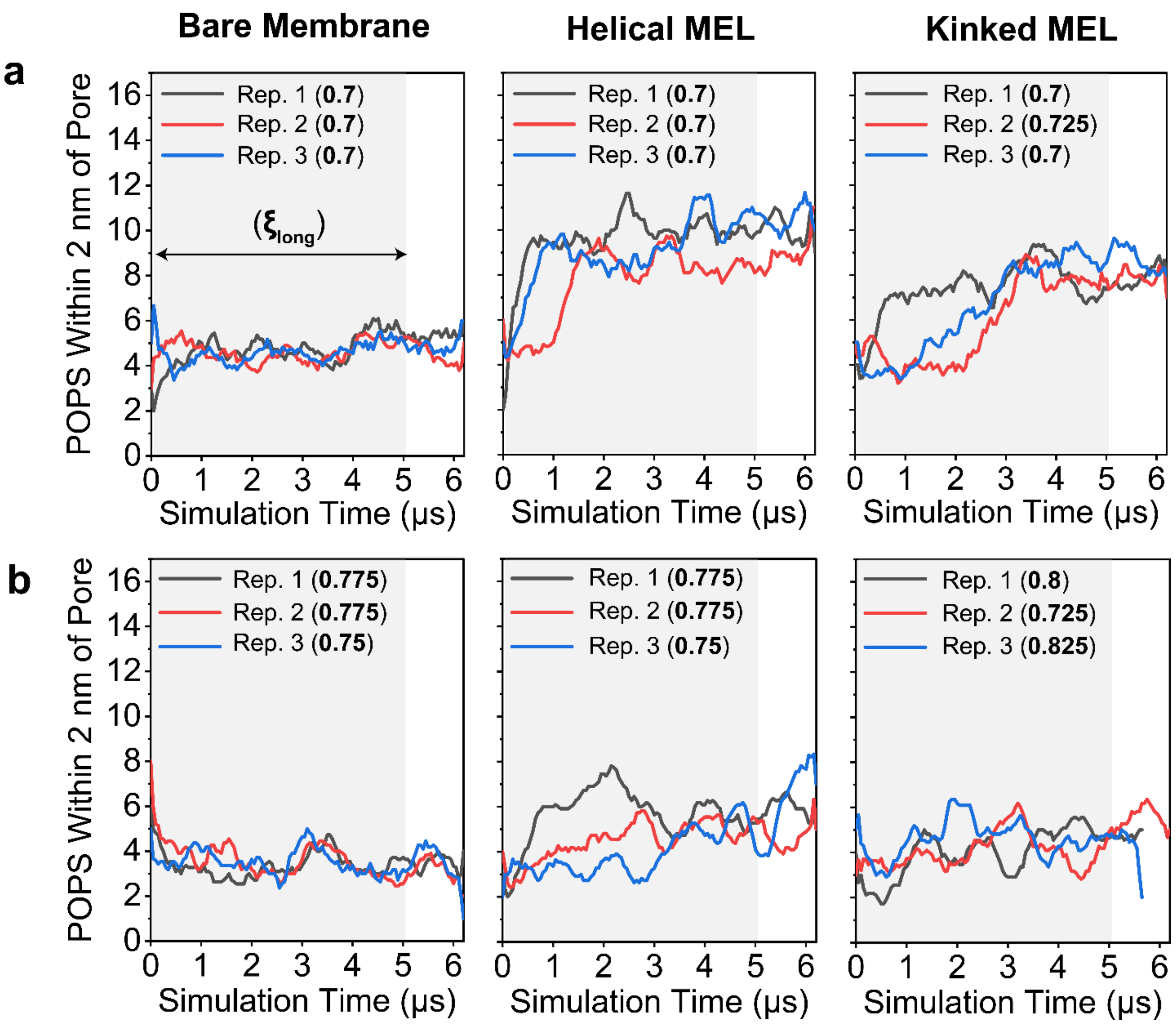

Figure S16: Number of POPS within 2 nm of the pore center for all replicas in support of **Figure 3b** for systems containing (a) 0% CHOL and (b) 50% CHOL. Results are plotted after smoothing with adjecent averaging with 10 points. Helical MEL and kinked MEL results (rightmost 2 columns) are plotted for the selected ξ_{long} for each replica (**Table S3**), with ξ_{long} values included in parentheses in the legend. Bare membrane results use the same ξ_{long} as the helical MEL systems. The light grey region corresponds to the 5 μs of simulation restrained at the ξ_{long} selected for each system from t = 50 ns to t = 5.05 μs.

S6: Additional PMFs and Convergence

Figure S17 shows MARTINI PMF profiles for the 4 bare membrane systems considered in this work. PMFs are obtained from 100 ns of sampling per umbrella sampling window for consistency with our previous work.[5] These PMFs demonstrate both the overestimation of pore formation free energies calculated with MARTINI compared to atomistic representations (**Figures 4-5** of the main text) and the inability of MARTINI to differentiate between POPC and RBC membranes.

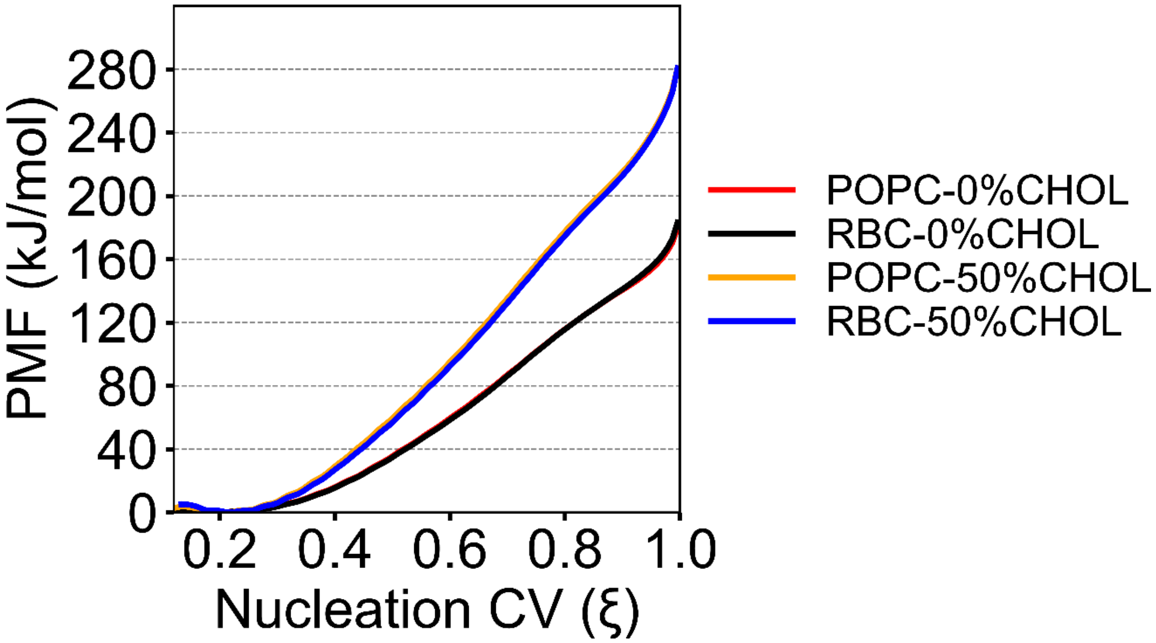

Figure S17: MARTINI potential of mean force (PMF) profiles for bare membrane POPC-0%CHOL (red), RBC-0%CHOL (black), POPC-50%CHOL (orange), and RBC-50%CHOL (blue) systems. PMFs are plotted as the average across 3 replicas with standard errors across the replicas indicated by shaded regions.

Additionally, we provide PMF convergence analysis for all bare membrane (**Figure S18**), helical MEL (**Figure S19**), and kinked MEL (**Figure S20**) systems to demonstrate that 40 ns of sampling per window is sufficient for atomistic umbrella sampling simulations.

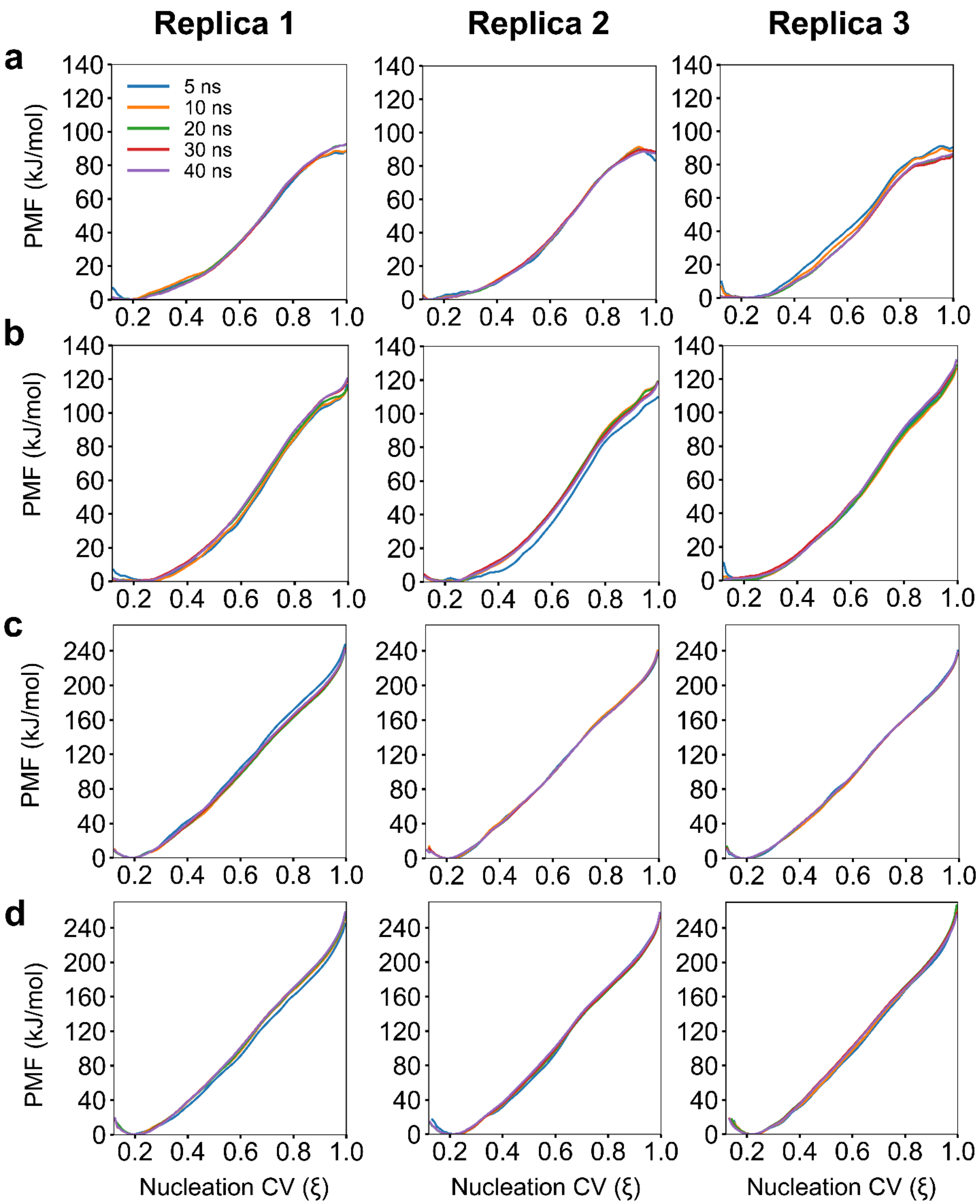

Figure S18: Bare membrane PMF convergence for (a) POPC-0%CHOL, (b) RBC-0%CHOL, (c) POPC-50%CHOL, (d) RBC-50%CHOL systems across 3 replicas. Each PMF is the result of a WHAM calculation using the first 'n' ns of sampling (shown in legend).

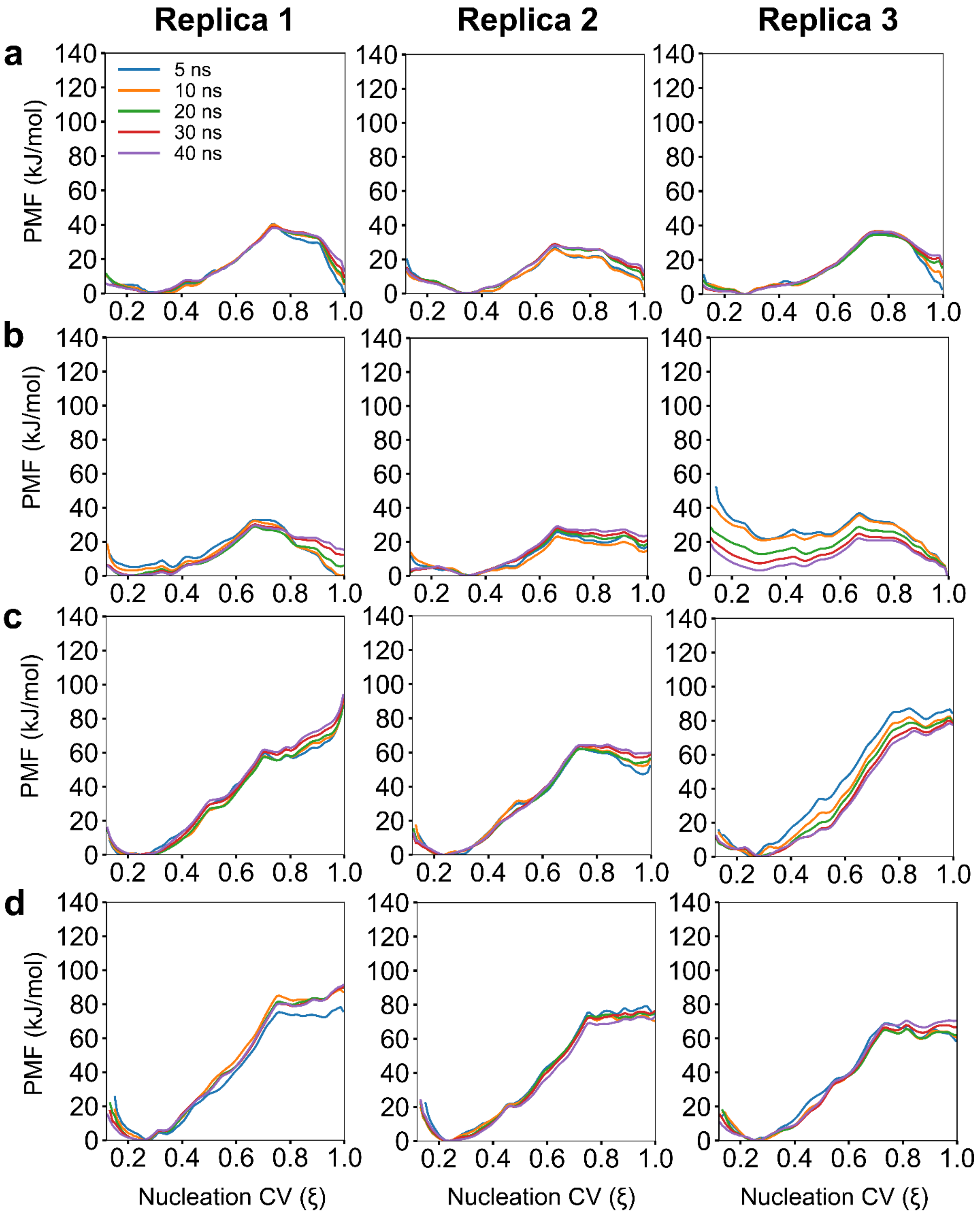

Figure S19: Helical MEL PMF convergence for (a) POPC-0%CHOL, (b) RBC-0%CHOL, (c) POPC-50%CHOL, (d) RBC-50%CHOL systems across 3 replicas. Each PMF is the result of a WHAM calculation using the first ‘n’ ns of sampling (shown in legend).

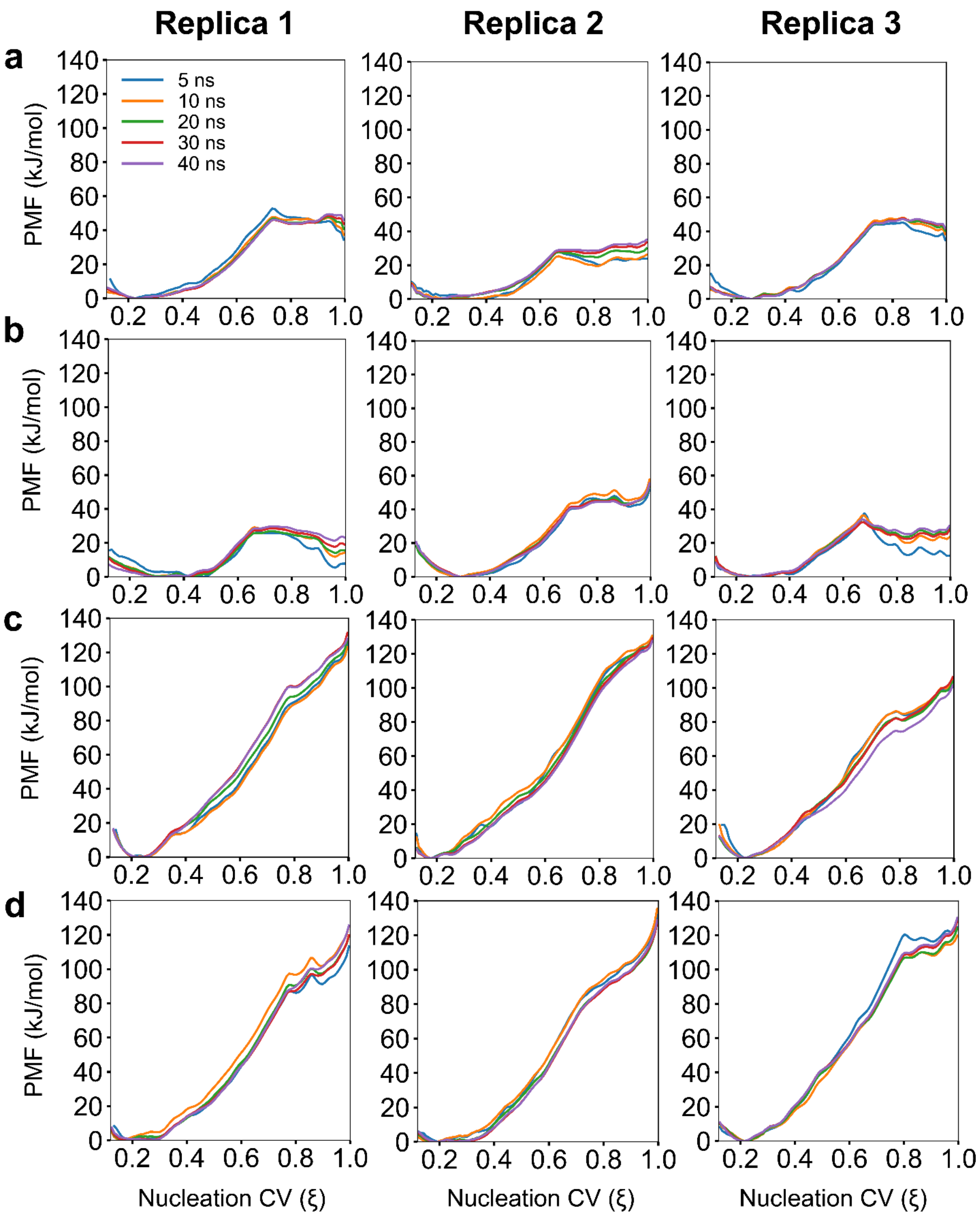

Figure S20: Kinked MEL PMF convergence for (a) POPC-0%CHOL, (b) RBC-0%CHOL, (c) POPC-50%CHOL, (d) RBC-50%CHOL systems across 3 replicas. Each PMF is the result of a WHAM calculation using the first ‘n’ ns of sampling (shown in legend).

Figure S21 is provided to support **Figure 4a** in the main text, where we observed that helical MEL form metastable, lower energy pores compared to kinked MEL in POPC-0%CHOL membranes at full nucleation (ξ = 1.0). **Figure S21a** demonstrates a similar trend for MEL in RBC-0%CHOL membranes. In 50% CHOL membranes, helical MEL-lined pores are more energetically favorable by 41.9 – 50.1 kJ/mol compared to kinked MEL-lined pores (**Figures S21b-c**). We used these observations as rationale for focusing on helical MEL-lined pores for umbrella sampling trajectory analysis in subsequent sections in the main text.

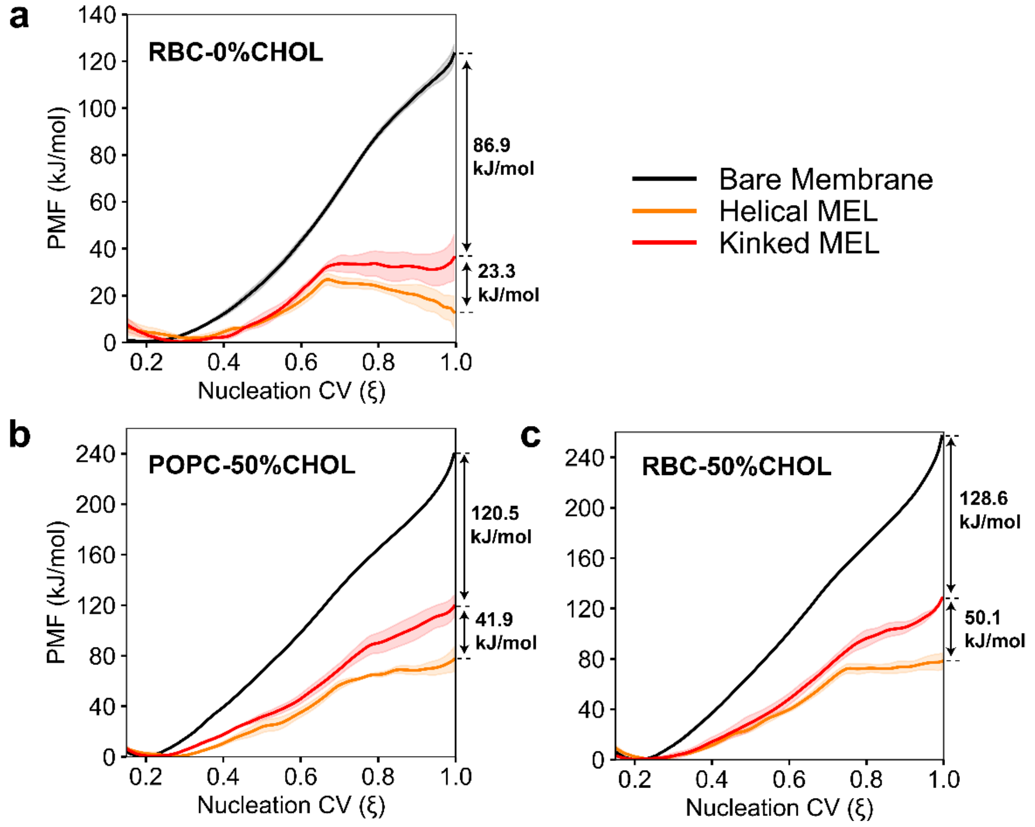

Figure S21: Potential of mean force (PMF) profiles for (a) RBC-0%CHOL, (b) POPC-50%CHOL, (c) RBC-50%CHOL systems. **Figure 4a** shows a corresponding PMF for the POPC-0%CHOL system. PMFs are plotted as the average across 3 replicas with standard errors across the replicas indicated by shaded regions. PMF differences at ξ = 1.0 are shown with arrows and corresponding captions.

S7: Supporting Data for Atomistic Umbrella Sampling Trajectories

This section contains additional information to support atomistic umbrella sampling trajectory analysis (**Figures 6–8**) of bare membrane vs MEL PMF profiles (**Figures 4–5**).

Table S4 documents the number of pore-lining MEL at the end of 50 ns of sampling for the umbrella sampling window corresponding to a fully nucleated transmembrane pore (ξ = 1.0). Results are tabulated for each of the 4 membrane types (POPC and RBC with and without 50% CHOL) with either 8 helical or kinked membrane MEL. The total number of pore-lining MEL for each system are provided in the rightmost column, which are divided into the total number of transmembrane (T-line) and U-shaped (U-line) MEL that line pores. Results indicate the average number of pore-lining MEL across 3 independent replicas with standard deviations.

A visualization of the pore-lining behavior of transmembrane (T-line) vs. U-shaped (U-line) MEL is shown in **Figures 4b-c**. T-lining MEL are characterized by strong α-helical secondary structure and anchoring of termini in opposing membrane leaflets, whereas U-lining MEL anchor both termini in the upper leaflet and partially line the pore with a mid-helix kink, which has been observed in previous MD[12] but not experimental studies. **Table S4** shows that there is no U-lining behavior when MEL is modeled with a rigid α-helical secondary structure during MARTINI simulations before backmapping (helical MEL), whereas on average 1 U-lining MEL is observed when MEL is modelled with a flexible mid-helix kink during MARTINI simulations (kinked MEL). Given that U-lining MEL obstruct the pore-lining ability of additional T-lining MEL (**Table S4**) and most likely result from an overestimation of the flexibility of the mid-helix kink during MARTINI simulations (since helical MEL lining is more energetically favorable, see **Figures 4a, S21**), we focused our PMF trajectory analysis on membrane and MEL-lining properties relating to bare membrane vs. 8 Helical MEL systems.

Table S4: Number of helical versus kinked MEL lining fully nucleated pores after 50 ns of simulation for ξ=1.0. Each row is divided into the average number of transmembrane (T-line), U-shaped (U-line), and total number of pore-lining MEL across 3 independent replicas with corresponding standard deviations.

			Pore-lining MEL		
		**System**	T-line	U-line	Total
**0% CHOL**	**POPC**	Helical MEL	5.3 ± 1.2	——	**5.3 ± 1.2**
		Kinked MEL	*2 ± 0*	*1 ± 1*	***3 ± 1***
	**RBC**	Helical MEL	5 ± 1	——	**5 ± 1**
		Kinked MEL	*2.7 ± 0.6*	*1 ± 0*	***3.7 ± 0.6***
**50% CHOL**	**POPC**	Helical MEL	3.3 ± 0.6	——	**3.3 ± 0.6**
		Kinked MEL	*1 ± 0*	*1 ± 1*	***2 ± 1***
	**RBC**	Helical MEL	3.3 ± 0.6	——	**3.3 ± 0.6**
		Kinked MEL	*0.7 ± 0.6*	*1.3 ± 0.6*	***2 ± 0***

To relate differences in bare membrane PMFs (dashed lines in **Figure 5**) to the ability of each membrane to resist membrane deformation, we calculated the area compressibility modulus (K_A) for each bare atomistic membrane with additional 500 ns unbiased simulations. Higher K_A values indicate stiffer membranes that are less prone to deformation.[13] K_A is calculated with the following equation:

$$K_A = \frac{A k_B T}{{\sigma_A}^2} \tag{S2}$$

where A is the average membrane area, k_B is Boltzmann's constant, T is temperature, and ${\sigma_A}^2$ is the mean-square fluctuation of the area. K_A calculated for POPC-0%CHOL (214 mN/m) is in good agreement with previous MD and experimental studies[14, 15], and differences in K_A (labelled in **Figure S22**) are consistent with bare membrane PMF slopes in **Figure 5**.

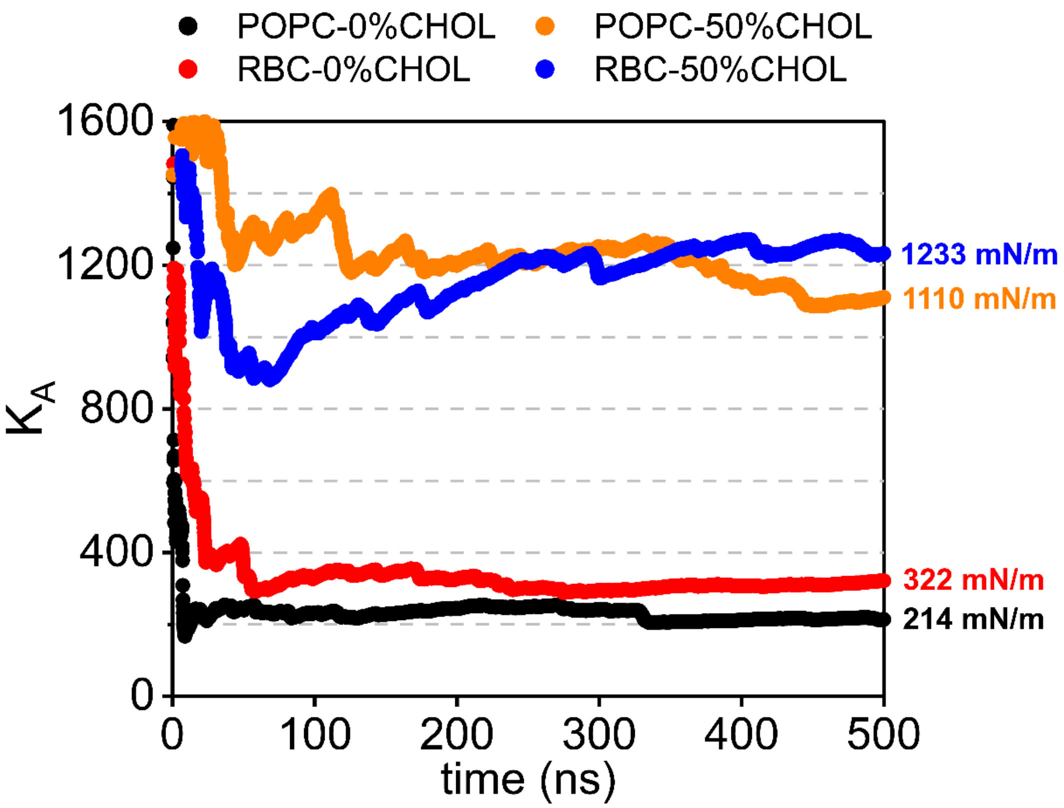

Figure S22: Area compressibility modulus (K_A) convergence during 500 ns atomistic simulations of bare POPC-0%CHOL (black), RBC-0%CHOL (red), POPC-50%CHOL (orange), and RBC-50%CHOL (blue) membranes. Final K_A values are labelled for each system in units of mN/m.

We additionally calculated K_A values for membranes restrained at different pore sizes in the presence of MEL to determine if MEL promotes pore nucleation by increasing membrane fluidity (decreasing K_A) compared to bare membranes. We ran four additional 350 ns atomistic simulations (restrained at ξ = 0.2, 0.65, 0.7, 1.0) starting from the final atomistic umbrella sampling configurations, and average simulation K_A results are visualized as red bars in **Figure S23a**. These data show that MEL membrane binding (**Figure S23c**) and subsequent pore nucleation (**Figure S23d**) decrease K_A by roughly 15-20% compared to the bare membrane case (**Figure S23b**).

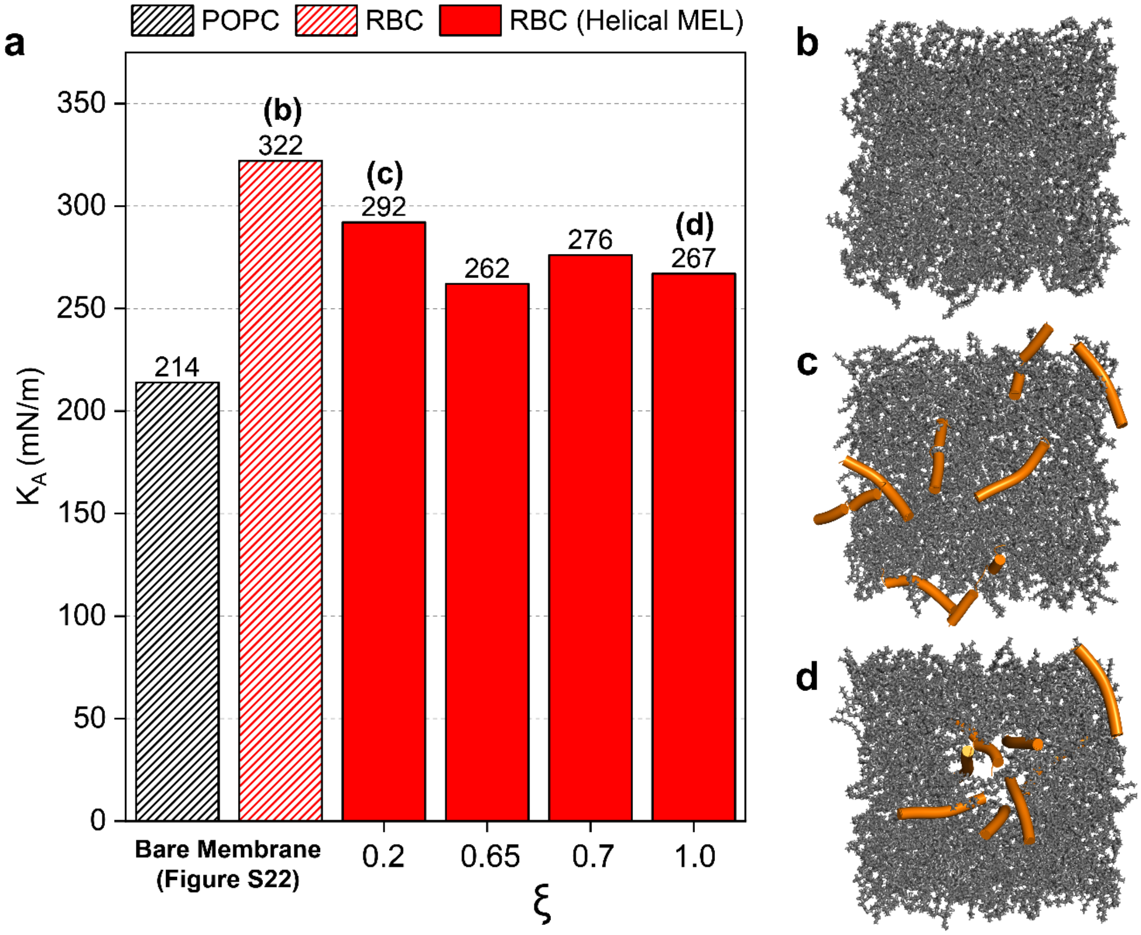

Figure S23: Area compressibility moduli (K_A) for RBC-0%CHOL membranes containing 8 helical MEL. (a) K_A values comparing bare POPC-0%CHOL (dashed black bar) and RBC-0%CHOL (dashed red bar) results from **Figure S22** to the RBC-0%CHOL membrane with 8 helical MEL (red bars) restrained at 4 different pore sizes (ξ = 0.2, 0.65, 0.7, 1.0). (b-d) Corresponding simulation snapshots for the RBC-0%CHOL membrane with (b) no MEL, (c) 8 helical MEL restrained at ξ = 0.2, and (d) 8 helical MEL restrained at ξ = 1.0. All lipids are colored as gray sticks and all MEL are colored as orange cartoons. Solvent is excluded from the visualization for clarity.

Figure S24 presents MEL tilt angle vs. time for all 8 peptides of each helical MEL ξ = 1 umbrella sampling window simulation (average tilt angles of pore-lining MEL are presented in **Figure 7a**).

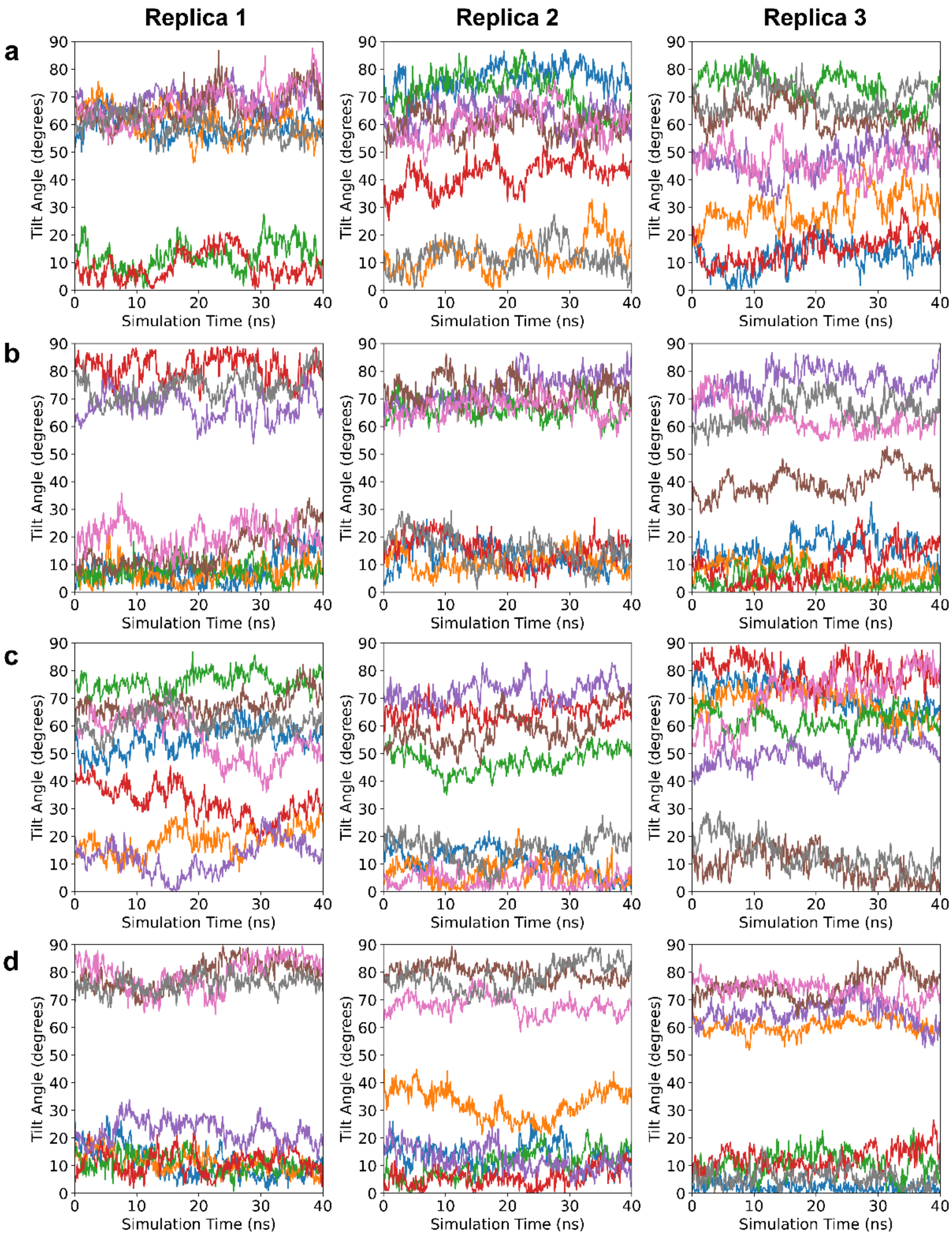

Figure S24: MEL tilt angle vs simulation time for the ξ=1 umbrella sampling window for all helical MEL systems: (a) POPC-0%CHOL, (b) POPC-50%CHOL, (c) RBC-0%CHOL, (d) RBC-50%CHOL.

Figure S25 is provided for Replica 1 of the RBC-0%CHOL system to demonstate that MEL tilt angles are well-converged for a number of pore sizes in the MEL pore-lining range ($0.7 \leq \xi \leq 1.0$) to further demonstrate the convergence of umbrella sampling for MEL-containing systems (**Figures S19-S20**).

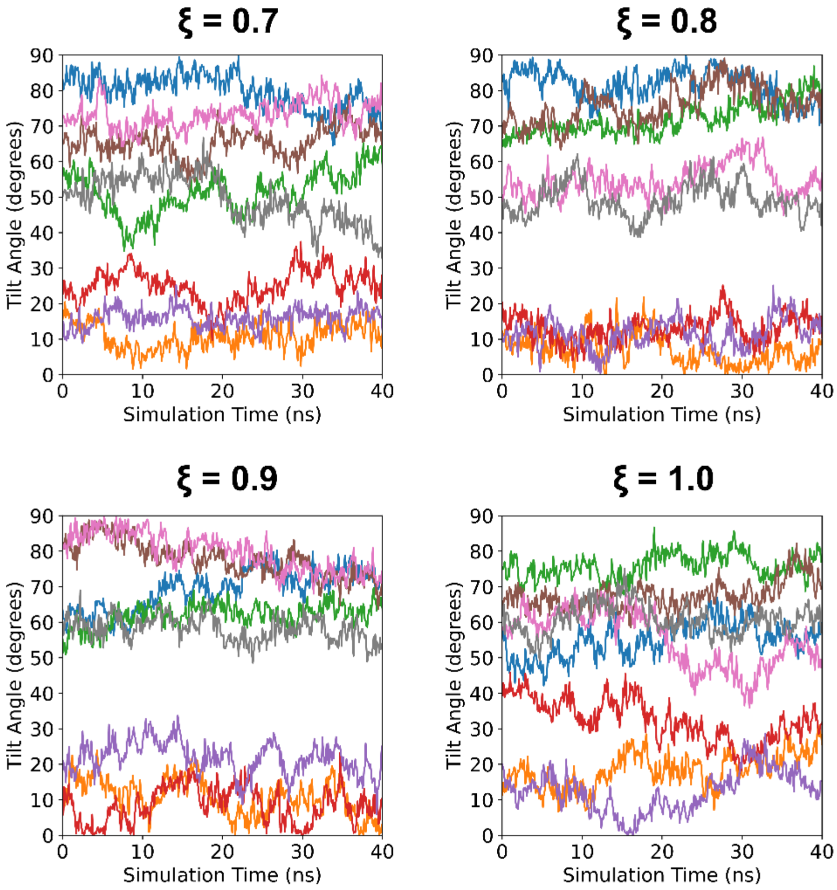

Figure S25: MEL tilt angle vs simulation time for several umbrella sampling windows for Replica 1 of the RBC-0%CHOL system. The plot for $\xi = 1.0$ is identical to the plot in the Replica 1 column of **Figure S24c**.

Figure S26 visualizes final MEL-lined pore structures for replicas 2 and 3 of the atomistic helical MEL umbrella sampling simulations restrained at ξ=1.0.

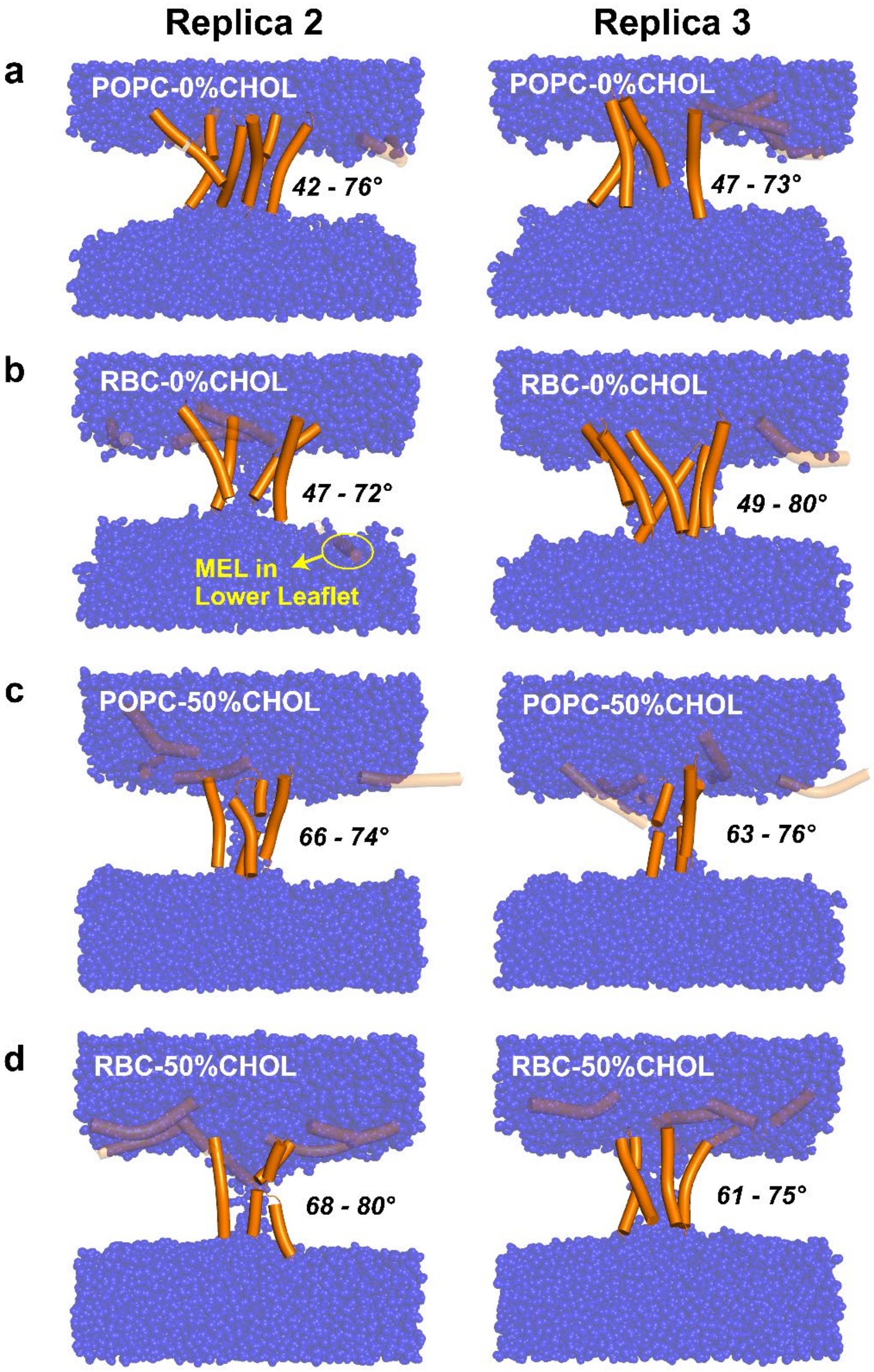

Figure S26: Representative simulation snapshots of pore-lining MEL (opaque orange cylinders) for replicas 2 and 3 for (a) POPC-0%CHOL, (b) RBC-0%CHOL, (c) POPC-50%CHOL, and (d) RBC-50%CHOL. Snapshots correspond to the final configuration of the $\xi = 1.0$ umbrella sampling window. Tilt angle ranges for pore-lining MEL are labelled in black (corresponding to tilt angles plotted in **Figure 7a** for Replicas 2 and 3). Water molecules are colored as transparent blue spheres and MEL that are not lining the pore are colored as transparent orange cartoons. (b) A MEL that flipped to the lower leaflet for Replica 2 of the RBC-0%CHOL system is circled in yellow.

To verify the statistical significance of MEL tilt angle mean differences presented in **Figure 7a**, we conducted a two-tailed t-test between all pairs of systems, with resulting p-values presented in **Table S5**. P-values < 0.05 for 0% vs. 50% systems for both POPC and RBC membranes (bolded) signify that increases in the average tilt angle for pore-lining MEL in the presence of 50% membrane CHOL (gold vertical arrows in **Figure 7a**) are statistically significant.

Table S5: P-values from a two-tailed t-test for the pore-lining MEL tilt angles plotted in **Figure 7a**. Bold values denote that differences in average tilt angles for membranes in the absence (0% CHOL) and presence (50 % CHOL) of CHOL are statistically significant for both POPC and RBC membranes.

System 1	System 2	p-value
**POPC-0%CHOL**	**POPC-50%CHOL**	**0.015**
POPC-0%CHOL	RBC-0%CHOL	0.790
POPC-0%CHOL	RBC-50%CHOL	0.003
POPC-50%CHOL	RBC-0%CHOL	0.039
POPC-50%CHOL	RBC-50%CHOL	0.333
**RBC-0%CHOL**	**RBC-50%CHOL**	**0.008**

To estimate differences in pore size for the atomistic umbrella simulations restrained at ξ=1.0, we estimated the average number of water molecules in the middle z = 1 nm of the pore as shown in **Figure S27**. For this calculation, we first computed the number density profile of water O atoms using the *gmx density* tool with 500 box slices in the *z* direction. We then multiplied the number density of water O atoms in each slice (nm^{-3}) by the slice volume during the trajectory, which was calculated by multipying the slice thickness by the average box *xy*-area determined with *gmx energy* and summed the resulting water O counts between z = -0.5 nm and z = 0.5 nm. Pore sizes are in good agreement with PMF metastability trends observed in **Figure 5**.

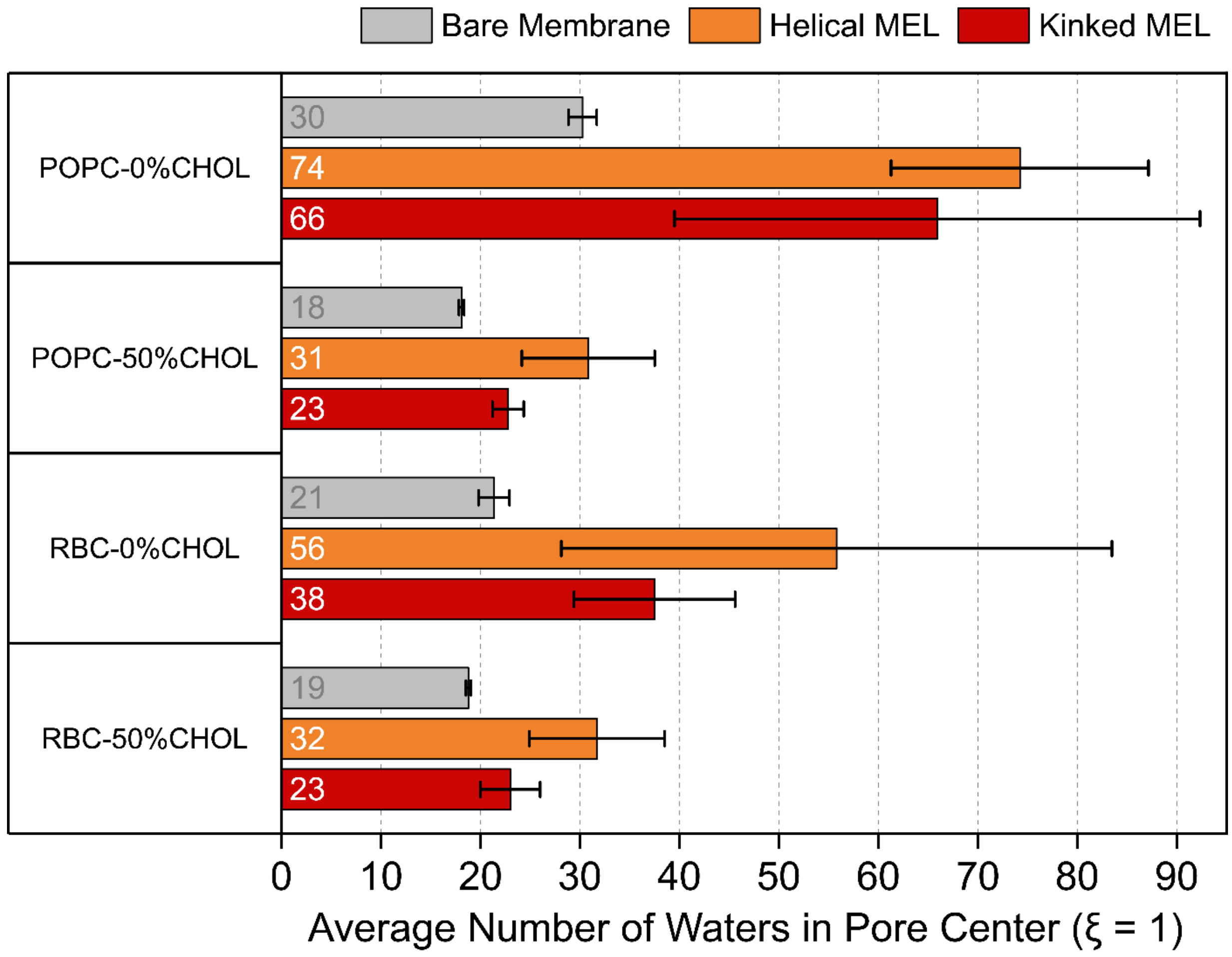

Figure S27: Water count in the middle z = 1 nm of the transmembrane pore at ξ = 1.0. Results are plotted as the average across 3 replicas for each membrane with standard deviations represented as error bars.

References

(1) de Jong, D. H.; Singh, G.; Bennett, W. F. D.; Arnarez, C.; Wassenaar, T. A.; Schäfer, L. V.; Periole, X.; Tieleman, D. P.; Marrink, S. J. Improved Parameters for the Martini Coarse-Grained Protein Force Field. *Journal of Chemical Theory and Computation* **2013**, *9* (1), 687-697. DOI: 10.1021/ct300646g.

(2) Roland, B. P.; Graham, T. R. Directed evolution of a sphingomyelin flippase reveals mechanism of substrate backbone discrimination by a P4-ATPase. *Proceedings of the National Academy of Sciences* **2016**, *113* (31), E4460-E4466. DOI: 10.1073/pnas.1525730113.

(3) Melo, M. N.; Ingólfsson, H. I.; Marrink, S. J. Parameters for Martini sterols and hopanoids based on a virtual-site description. *The Journal of Chemical Physics* **2015**, *143* (24), 243152. DOI: 10.1063/1.4937783.

(4) Ozturk, T. N.; König, M.; Carpenter, T. S.; Pedersen, K. B.; Wassenaar, T. A.; Ingólfsson, H. I.; Marrink, S. J. Building complex membranes with Martini 3. *Methods Enzymol* **2024**, *701*, 237-285. DOI: 10.1016/bs.mie.2024.03.010.

(5) Richardson, J. D.; Van Lehn, R. C. Free Energy Analysis of Peptide-Induced Pore Formation in Lipid Membranes by Bridging Atomistic and Coarse-Grained Simulations. *The Journal of Physical Chemistry B* **2024**, *128* (36), 8737-8752. DOI: 10.1021/acs.jpcb.4c03276.

(6) Hub, J. S.; Awasthi, N. Probing a Continuous Polar Defect: A Reaction Coordinate for Pore Formation in Lipid Membranes. *Journal of Chemical Theory and Computation* **2017**, *13* (5), 2352-2366. DOI: 10.1021/acs.jctc.7b00106.

(7) Wassenaar, T. A.; Pluhackova, K.; Böckmann, R. A.; Marrink, S. J.; Tieleman, D. P. Going Backward: A Flexible Geometric Approach to Reverse Transformation from Coarse Grained to Atomistic Models. *Journal of Chemical Theory and Computation* **2014**, *10* (2), 676-690. DOI: 10.1021/ct400617g.

(8) Jo, S.; Kim, T.; Iyer, V. G.; Im, W. CHARMM-GUI: A web-based graphical user interface for CHARMM. *Journal of Computational Chemistry* **2008**, *29* (11), 1859-1865. DOI: https://doi.org/10.1002/jcc.20945.

(9) Wassenaar, T. A.; Ingólfsson, H. I.; Böckmann, R. A.; Tieleman, D. P.; Marrink, S. J. Computational Lipidomics with insane: A Versatile Tool for Generating Custom Membranes for Molecular Simulations. *Journal of Chemical Theory and Computation* **2015**, *11* (5), 2144-2155. DOI: 10.1021/acs.jctc.5b00209.

(10) Piggot, T. J.; Allison, J. R.; Sessions, R. B.; Essex, J. W. On the Calculation of Acyl Chain Order Parameters from Lipid Simulations. *Journal of Chemical Theory and Computation* **2017**, *13* (11), 5683-5696. DOI: 10.1021/acs.jctc.7b00643.

(11) Marrink, S. J.; Risselada, H. J.; Yefimov, S.; Tieleman, D. P.; de Vries, A. H. The MARTINI Force Field: Coarse Grained Model for Biomolecular Simulations. *The Journal of Physical Chemistry B* **2007**, *111* (27), 7812-7824. DOI: 10.1021/jp071097f.

(12) Sun, L.; Wang, S.; Tian, F.; Zhu, H.; Dai, L. Organizations of melittin peptides after spontaneous penetration into cell membranes. *Biophysical Journal* **2022**, *121* (22), 4368-4381.

(13) Moradi, S.; Nowroozi, A.; Shahlaei, M. Shedding light on the structural properties of lipid bilayers using molecular dynamics simulation: a review study. *RSC Advances* **2019**, *9* (8), 4644-4658, 10.1039/C8RA08441F. DOI: 10.1039/C8RA08441F.

(14) Saeedimasine, M.; Montanino, A.; Kleiven, S.; Villa, A. Role of lipid composition on the structural and mechanical features of axonal membranes: a molecular simulation study. *Scientific Reports* **2019**, *9* (1), 8000. DOI: 10.1038/s41598-019-44318-9.

(15) Binder, H.; Gawrisch, K. Effect of Unsaturated Lipid Chains on Dimensions, Molecular Order and Hydration of Membranes. *The Journal of Physical Chemistry B* **2001**, *105* (49), 12378-12390. DOI: 10.1021/jp010118h.
